# Effects of downstream genes on synthetic genetic circuits

**DOI:** 10.1186/1752-0509-8-S4-S4

**Published:** 2014-12-08

**Authors:** Takefumi Moriya, Masayuki Yamamura, Daisuke Kiga

**Affiliations:** 1 Department of Computational Intelligence and Systems Science, Tokyo Institute of Technology, Kanagawa 226-8503, Japan; 2 Earth-Life Science Institute, Tokyo Institute of Technology, Meguro, Tokyo 152- 8551, Japan

**Keywords:** synthetic biology, genetic circuit, mathematical modeling, protein degradation, impedance, decoy site

## Abstract

**Background:**

In order to understand and regulate complex genetic networks in living cells, it is important to build simple and well-defined genetic circuits. We designed such circuits using a synthetic biology approach that included mathematical modeling and simulation, with a focus on the effects by which downstream reporter genes are involved in the regulation of synthetic genetic circuits.

**Results:**

Our results indicated that downstream genes exert two main effects on genes involved in the regulation of synthetic genetic circuits: (1) competition for regulatory proteins and (2) protein degradation in the cell.

**Conclusions:**

Our findings regarding the effects of downstream genes on regulatory genes and the role of impedance in driving large-scale and complex genetic circuits may facilitate the design of more accurate genetic circuits. This design will have wide applications in future studies of systems and synthetic biology.

## Background

**Figure 1:**
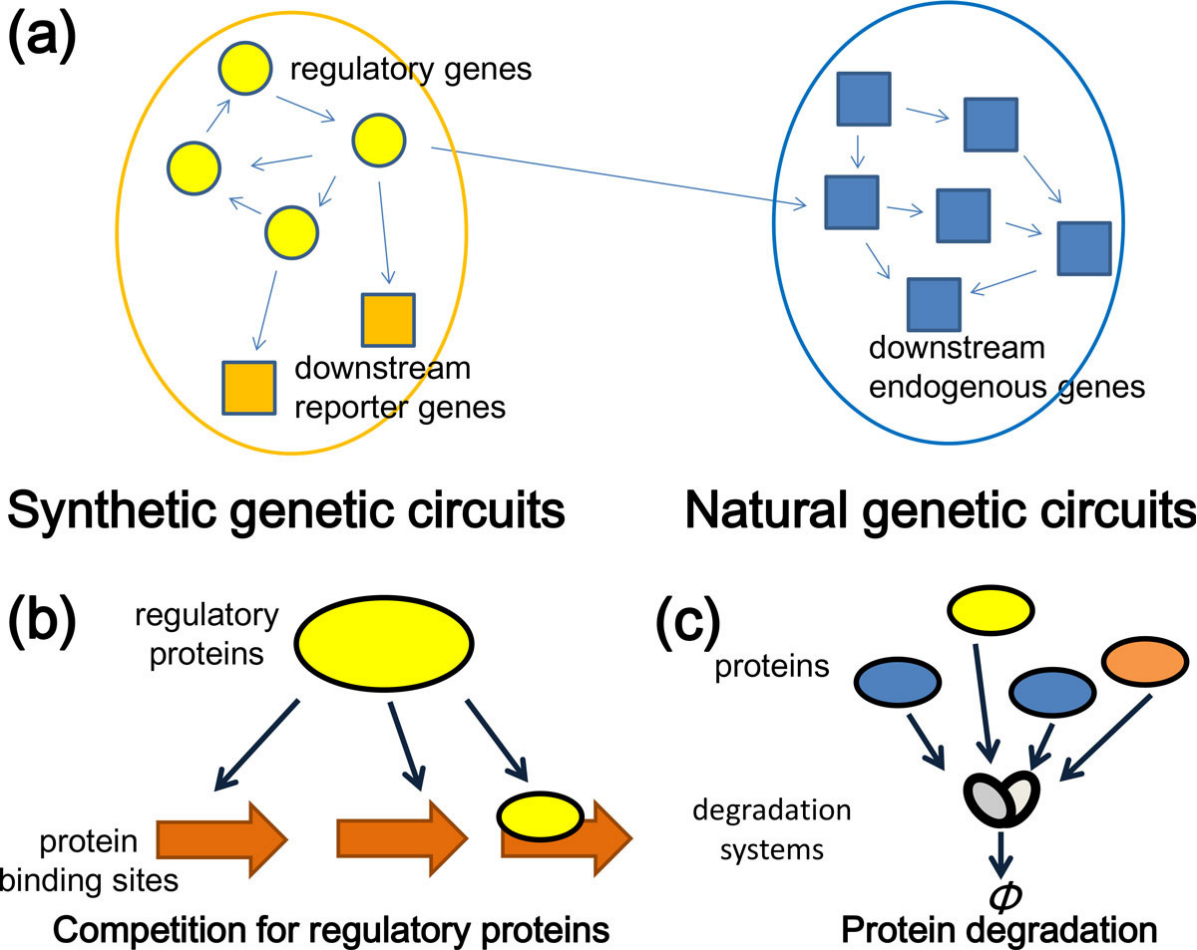
**The effect of downstream genes on regulatory genes in synthetic genetic circuits**. (a) Synthetic genetic circuits in a cell. Regulatory genes of synthetic genetic circuits have an effect on downstream genes, such as reporter genes or genes in natural genetic circuits. (b) Competition for regulatory-protein-binding sites among genes encoding regulatory proteins and an additional reporter gene in synthetic genetic circuits. (c) Protein degradation systems in a cell. Proteins often share the same degradation system.

Synthetic biology allows for the understanding of biological phenomena through mathematical modeling and simulation [1-5]. In order to control cells optimally, it is important to identify the relationships among cell dynamics in computational experiments and those in living cells. In computational experiments, synthetic genetic circuits are often designed such that they only comprise regulatory genes in synthetic genetic circuits, and do not include downstream genes, such as reporter genes or genes present in natural genetic circuits (Figure 1a) [1-5]. However, downstream genes, including reporter genes in particular, are essential for monitoring, analyzing, and exploiting synthetic genetic circuits in living cells.

Exogenous reporter genes can affect the dynamics of regulatory genes in synthetic genetic circuits in two ways. The first involves increased competition for regulatory proteins among the existing regulatory gene promoters and the additional reporter- gene promoters in synthetic genetic circuits (Figure 1b); binding of a regulatory protein to a decoy site on DNA is an example of such competition [6]. Decoy sites vary and are present in viruses, prokaryotes, and eukaryotes [7-9]. The second effect of introducing reporter genes into synthetic genetic circuits involves effects on protein degradation (Figure 1c). Protein degradation components, such as the ubiquitin- proteasome system, autophagy (lysozyme and cathepsin activity), caspase, γ-secretase, and calpain, play a key role in natural regulatory systems [10-14]. The dynamics of synthetic regulatory circuits are modified by altered degradation of regulatory proteins that results from the use of downstream reporter proteins when the same degradation mechanism is involved. Together, these two effects of employing downstream genes are important factors to consider in the control of cells through mathematical modeling and simulation.

In this paper, we describe changes in the dynamics of regulatory genes in synthetic genetic circuits when employing downstream genes. Here, we evaluated the effect of connecting a synthetic oscillating regulatory circuit with a reporter gene. We found that protein degradation plays a central role, and is as important as protein production [15], because the downstream genes exhibit competition for regulatory proteins among binding sites on the DNA and so compete for protein degradation machinery.

## Results and discussion

### Mathematical modeling for evaluation of the effects of downstream genes on oscillators

**Figure 2:**
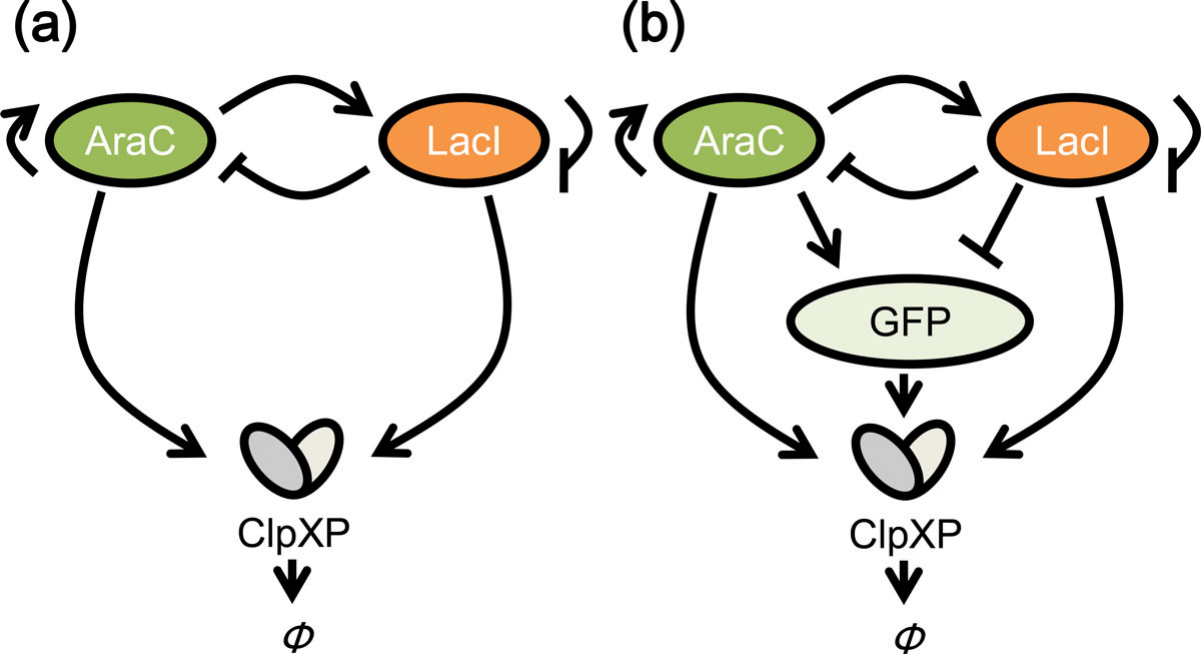
**Gene network models of the effects of downstream genes**. (a) The reporter-less model. (b) The reporter-containing model. AraC and LacI form positive and negative feedback loops, respectively. GFP is the downstream gene of this oscillator. ClpXP is the protease that recognizes and cleaves the SsrA-tagged proteins (AraC, LacI, and GFP).

We here describe two oscillator network models for monitoring the effects of downstream genes (Figure 2). One model is a reporter-less model, which has been described previously [4]. By addition of a reporter gene, we created the other model, called the reporter-containing model.

These two models share a regulatory gene-based oscillator, called the Smolen oscillator [16,17], which is dependent on protein degradation and feedback from protein synthesis. The Smolen oscillator is known to be robust and tunable. In the oscillator, AraC and LacI form positive and negative feedback loops. AraC is a protein that is activated in the presence of arabinose. LacI acts as a repressor protein in the absence of isopropyl β-D-1-thiogalactopyranoside (IPTG). In these models, AraC and LacI proteins were tagged with the SsrA peptide and thus were specifically degraded efficiently by the ClpXP protease [18,19].

The additional gene in the second model is a downstream green fluorescent protein (GFP) reporter gene (Figure 2b). Although downstream genes are very complex in living cells, we, for simplicity's sake, utilized a single reporter gene in this study. Moreover, notably, AraC proteins and LacI proteins bind not only to the promoters driving their own expressions, but also to the GFP promoter. Consequently, GFP expression can be activated by AraC and be repressed by LacI. By means of the SsrA tag on GFP, this protein was also specifically degraded efficiently by the ClpXP protease. In contrast, in the reporter-less GFP model, the downstream component is absent (Figure 2a). Detailed mathematical modeling for these two models is shown in the Methods section.

### Addition of downstream genes affects the bifurcation of the oscillator

**Figure 3:**
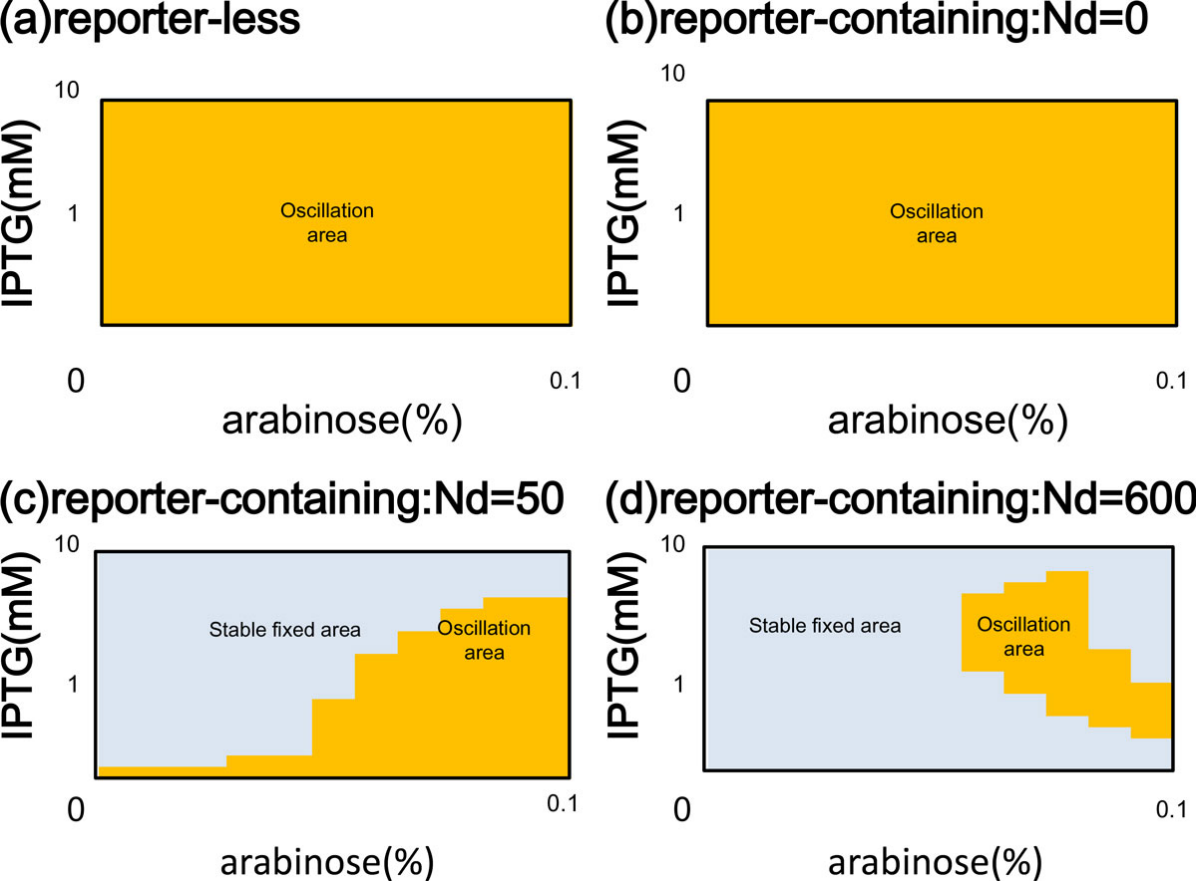
**Bifurcation diagrams for the reporter-less model and the reporter containing model**. (a) The reporter-less model. (b) The reporter-containing model (copy number of GFP gene: N_d _= 0). (c) N_d _= 50. (d) N_d _= 600. The behavior of the models with respect to arabinose concentration (X-axis) and IPTG concentration (Y-axis) is shown. The yellow area is the oscillation area and the grey area is the stable fixed area.

The reporter-less model exhibited a greater oscillation area in a bifurcation diagram than did the reporter-containing model (Figure 3). To describe the effects of the downstream components, we firstly investigated the bifurcation diagram for deterministic calculations of both models. The bifurcation diagram was defined by arabinose and IPTG concentration. Furthermore, in order to determine the competition for a regulatory protein, AraC or LacI, among its binding sites on the promoters of the synthetic regulatory-network genes and the reporter gene, we increased the copy number of GFP gene (Nd) in the reporter-containing model (Figure 3b-d). Note that the copy number of the regulatory gene is invariable. Consequently, the reporter-less model exhibited a wide oscillation area (Figure 3a). Similarly, for Nd = 0, the reporter-containing model also exhibited a wide oscillation area (Figure 3b). Increase of the copy number of the GFP gene expanded the fixed area (Figure 3c, d), where each oscillation with amplitude gradually decreases to constant (Additional File 1). These results suggested that the competition for a regulatory protein among its binding sites on the promoters affects oscillation bifurcation, similar to the effect caused by an increase in the number of decoy sites in a genetic circuit [6].

This and previous studies have analyzed competition for regulatory proteins among binding sites in the promoter regions and other regions of DNA. In one study of positive feedback, massive introduction of tandem-repeat "Decoy" DNA sequences decreased expression in living yeast by binding an activator protein, and also altered the inhibitory effect of a small molecule on the activator [6]. Another group analyzed circuit dynamics to demonstrate retroactivity, by which an upstream system receives information from downstream systems. In general, retroactivity causes time delay in signal transduction of upstream systems [20-22]. More particularly, it has been shown that a change in the copy number of reporter gene causes a change in the oscillation period [22], as was also demonstrated by our study. Our study further revealed that an increase in the copy number of reporter genes causes narrowing of the oscillating area in the bifurcation diagram.

### Competition for degradation machinery among proteins, including downstream proteins, results in a longer oscillator period

**Figure 4:**
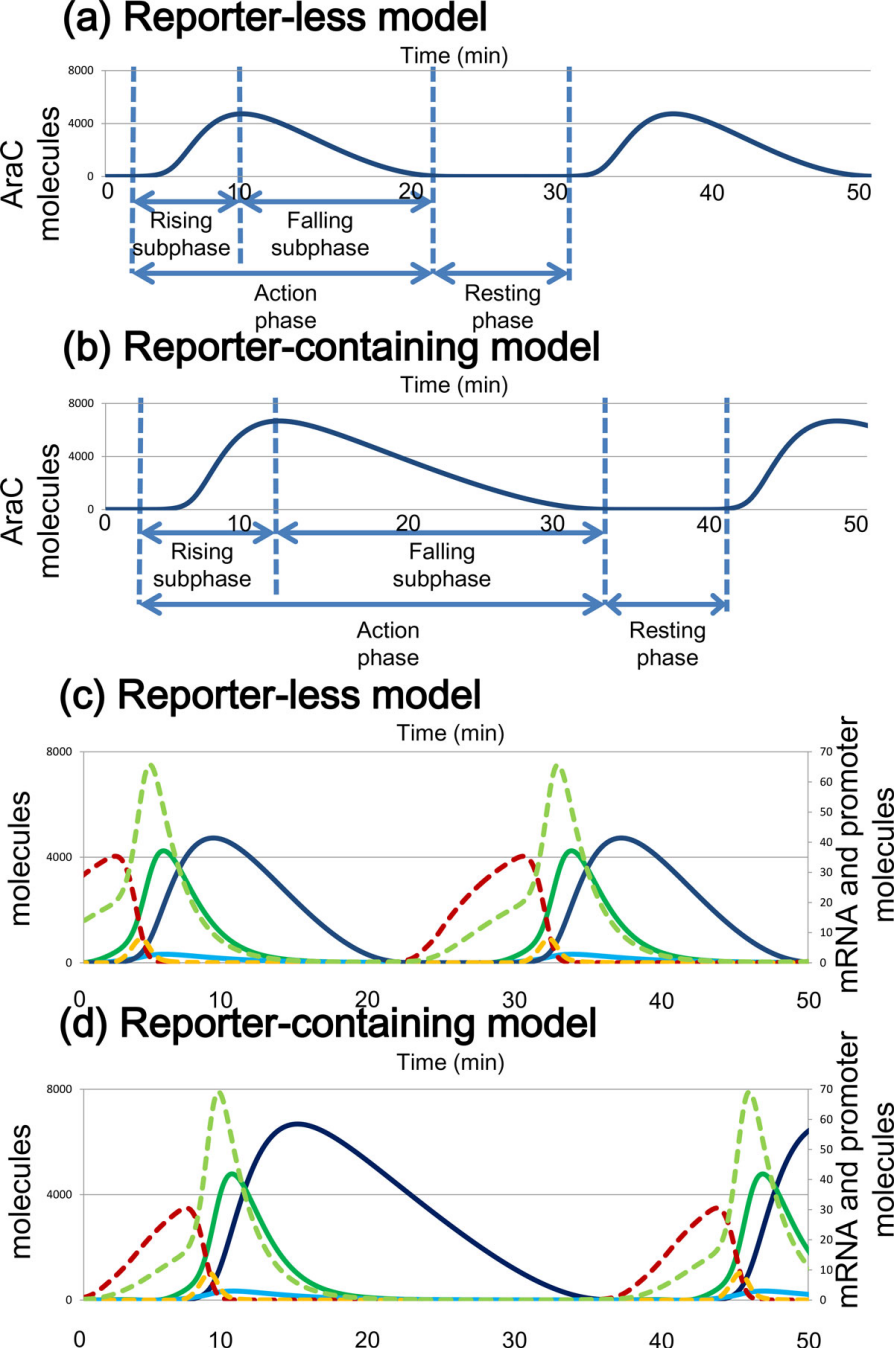
**Oscillation components in the time-course of the reporter-less model and the reporter-containing model by deterministic simulation**. Oscillation components in the two models at 1.0% arabinose and 10 mM IPTG. AraC dimer (dark blue) in (a) the reporter-less model and (b) the reporter-containing model. AraC composition in the two models at 1.0% arabinose and 10 mM IPTG. AraC dimer (dark blue), AraC mRNA (light green), AraC unfolded polypeptides (deep green), AraC monomer (light blue), non-binding AraC promoter (red), and one AraC- binding AraC promoter (orange) in (c) the reporter-less model and (d) the reporter- containing model.

Even in the same parameter set, where both models show oscillation, the reporter- containing model demonstrated a longer oscillation period than that of the reporter- less model (Figure 4a, b). For each of the parameter sets, the oscillation area of the reporter-containing model (Nd = 50) had a longer period than that seen for the corresponding parameter sets of the reporter-less model (Figure 3a, c and Additional File 2).

Detailed analysis of the time course of oscillation components in the two models showed that the longer total period in the reporter-containing model was mainly derived from the period where the rate of protein decay exceeded that of protein production. In the AraC-dimer time-course, for example, we defined the rising subphase, falling subphase, action phase, and resting phase (Figure 4a, b). The total length of the oscillation period was equal to the sum of the action phase and resting phase. The resting phase was defined as that phase in which fewer than 10 AraC dimer molecules exist. The action phase was equal to the sum of the rising subphase and falling subphase. In the rising subphase, the rate of protein production exceeded the rate of protein decay, whereas in the falling subphase, the rate of protein decay exceeded that of protein production. The length of the oscillation period, calculated using deterministic simulation in both models, mainly depended on the falling subphase, but not on the rising subphase or resting phase (Table 1). Similarly, the total length of the oscillation period in deterministic simulation of LacI concentration oscillation was also dependent on the falling subphase.

**Table 1:** Time-courses in the reporter-less model and the reporter-containing model by deterministic simulations

	Reporter-less model	Reporter-containing model
Rising subphase	8.0 min	10.0 min
Falling subphase	16.0 min	27.0 min
Resting phase	4.0 min	1.0 min
Total oscillation period	28.0 min	38.0 min

These values reflect simulations performed at 1.0% arabinose and 10 mM IPTG

The greater length of the falling subphase in the reporter-containing model than that in the reporter-less model mainly derived from a greater probability of degradation. Protein production in the reporter-less model was slightly higher than that in the reporter-containing model: the integrated activity of AraC mRNA, which is the area under the AraC mRNA oscillation time course divided by the time interval, was 330 in the reporter-less model compared to 310 in the reporter-containing model (Figure 4c, d; Table 2). However, the integrated activity of the AraC dimer protein in the reporter-containing model was much higher than that in the reporter-less model; the integrated activity of AraC dimer was 43000 in the reporter-less model vs. 74000 in the reporter-containing model. This difference in the integrated activity of the AraC dimer protein suggested reduced degradation in the reporter-containing model compared to the reporter-less model, because the amount of a protein in a cell is affected by both production and degradation. Indeed, in these models, three proteins (AraC, LacI, and GFP) compete with each other for the protein degradation system. As the reporter-less model does not contain GFP, AraC and LacI are degraded rapidly by the protein degradation system, and the threshold between the action phase and resting phase is reached early. Thus, additional downstream genes cause a longer falling subphase by generating competition for the degradation machinery among proteins. Very recently, similar competition for degradation machinery between proteins of two synthetic regulatory networks has been reported to cause oscillation coupling [23].

**Table 2:** Integrated activity of each of molecules in oscillation time course in the reporter-less model and the reporter-containing model by deterministic simulation

	Reporter-less model	Reporter-containing model
AraC P_00 _promoter	2.1 × 10^2^	1.1 × 10^2^
AraC P_10 _promoter	1.4 × 10	2.2 × 10
AraC transcription rate (AraC P_00 _+ P_10 _promoter)	4.9 × 10^2^	5.5 × 10^2^
AraC mRNA	3.3 × 10^2^	3.1 × 10^2^
AraC unfolding	2.1 × 10^4^	2.1 × 10^4^
AraC monomer	2.4 × 10^3^	2.7 × 10^3^
AraC dimer	4.3 × 10^4^	7.4 × 10^4^

These values performed at 1.0% arabinose and 10 mM IPTG.

### Design of synthetic genetic circuits by considering output impedance and input impedance

In order to drive large-scale and complex genetic circuits while using restricted resources in a cell, a situation similar to output impedance and input impedance in electric circuits should be considered when designing synthetic genetic circuits. In electric circuits, connection between electronic components may perturb the dynamics of the circuits. Similarly, previous modeling studies proposed that connections between regulatory genes and downstream gene perturb oscillation dynamics [20].

**Figure 5:**
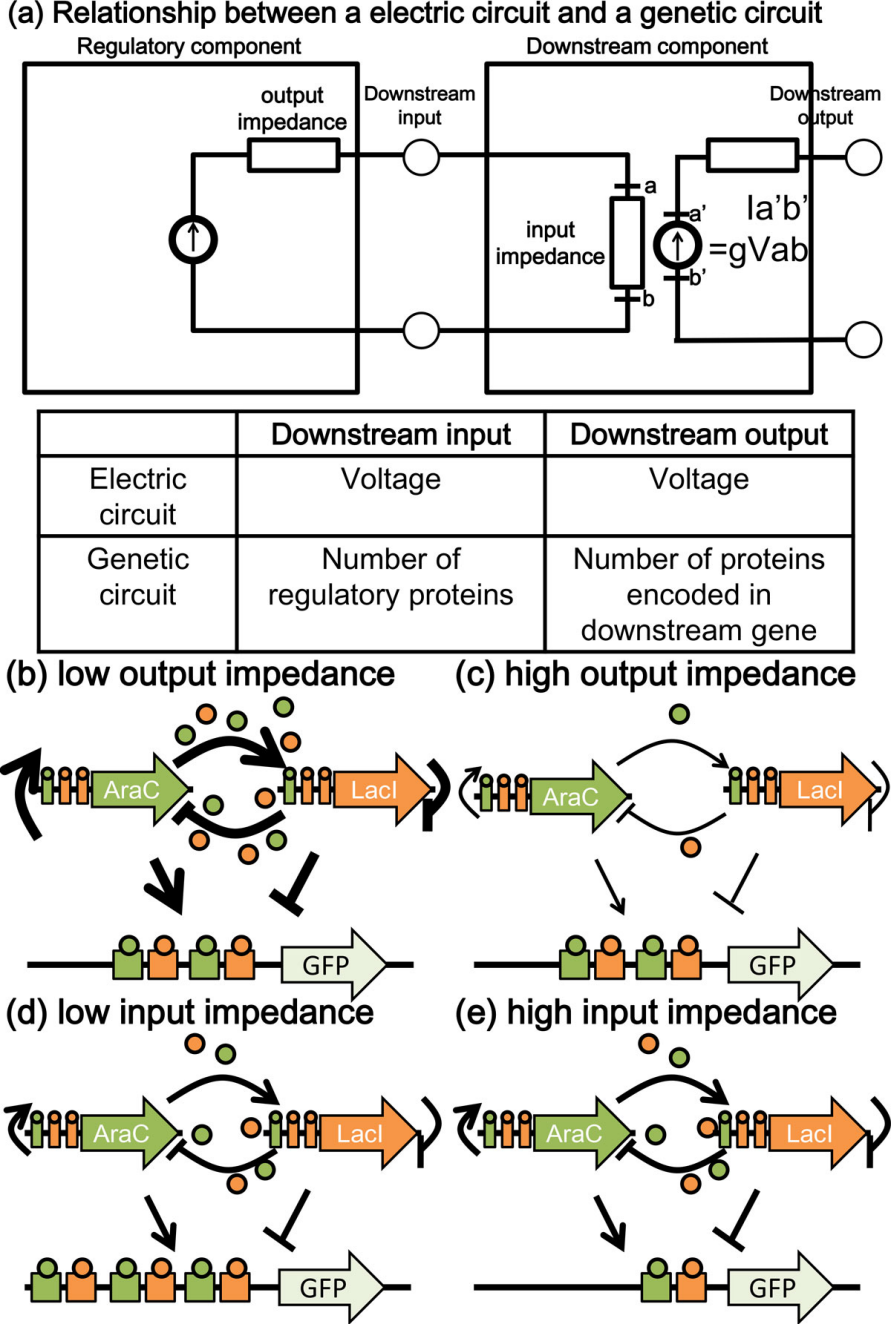
**Relationship of the effects of impedance between electric circuit and Genetic circuit**. (a) Relationship of the effects of impedance between electric circuit and genetic circuit. In electric circuit, downstream component outputs voltage in proportion to downstream input consumed by downstream input impedance. (b-e) Genetic circuit models of the effects of impedance. (b) The low output impedance model of genetic circuit models. (c) The high output impedance model of genetic circuit models. (d) The low input impedance model of genetic circuit models. (e) The high input impedance model of genetic circuit models. AraC and LacI form regulatory genes. GFP is the downstream gene. AraC protein and LacI protein represented by colored circles are accommodated in the binding sites of the GFP promoter. Numbers of colored circles represent concentrations of regulatory proteins. Low output impedance and high input impedance are suitable to keep dynamics of regulatory genes even after connection of the downstream gene.

Our mathematical modeling and simulation also showed that the connection of downstream genes changed the oscillation area in the bifurcation diagram and the oscillation period of regulatory genes (Figure 3, 4). In electric circuits, perturbation by the connection between electric components is indicated by input impedance and output impedance (Figure 5a). Electric circuits are composed by many components. Regulatory component is built constant current source. Voltage is defined by output impedance and input impedance of regulatory component. Downstream component outputs voltage in proportion to downstream input consumed by input impedance. The dynamics of regulatory component is changed by connection of downstream component, in case of high output impedance of regulatory component or low input impedance of downstream component.

Genetic circuits are composed by similar components which the number of regulatory proteins treats as voltage. Given this concept of impedance, improved designs of genetic circuits as the same topology as those used in this study, would require low output impedance of the regulatory genes consisting Smolen oscillator and high input impedance of downstream reporter genes (Figure 5b-e). In order to reduce the disturbance of regulatory genes by connection to downstream genes, a previous study proposed connection via insulators that do not affect regulatory genes from which downstream genes receive the signal [20]. Insulator is the device that reduces high output impedance of the regulatory genes and increases low input impedance of downstream genes. However, such a connection causes a time delay. In order to decrease output impedance, reflected by altered regulatory genes, we propose that the copy numbers and expression of the regulatory genes should be increased (Figure 5b). In this case, the dynamics of the altered regulatory genes with increased copy numbers should be confirmed in advance, because changing the copy numbers and expression of regulatory genes would alter the dynamics between regulatory genes. In order to increase input impedance, reflected by amended downstream reporter genes, we also propose that the number and affinity of DNA-binding sites in downstream genes should be decreased (Figure 5e). Note that this change would decrease the signal-to-noise ratio of the downstream reporter genes. This decrease in the signal-to- noise ratio corresponds to elevated expression of reporter genes of the editing ribosome-binding site sequence and increased imaging sensitivity of the measuring devices. Downstream genes express by the effects of positive correlation of the number of regulatory proteins. Downstream coding sequence is conceivable of a reporter gene or a regulatory gene expressing more downstream genes. In the case of latter, downstream component is conceivable of connection to more downstream genes.

## Conclusions

Our results showed the effects of downstream reporter genes on regulatory genes of synthetic genetic circuits. Our mathematical modeling and simulation suggested that downstream genes exhibit (1) competition for regulatory proteins among binding sites on the DNA and (2) competition for protein degradation machinery in the living cell. This competition by components of genetic circuit targets not only proteases in the protein degradation machinery, but also RNA polymerase, ribosomes, and chaperone proteins [24].

Moreover, in synthetic biology, the downstream genes can be endogenous genes in natural genetic circuits (Figure 1a) [25-27]. Through interactions with downstream genes, synthetic genetic circuits sometimes exert unintentional negative effects on cellular viability [28,29], which can alter the dynamics of the circuits. Synthetic genetic circuits are also dependent on growth rate [28]. As growth rate increases, in a repressilator circuit, the stable fixed point enlarges, amplitude reduces, and the oscillation period shortens. In other examples [29], growth rate is dependent on synthetic genetic circuits. In modeling symbiosis of a tryptophan auxotroph metabolizing tyrosine and a tyrosine auxotroph metabolizing tryptophan, the synthesis of each amino acid regulates the growth rate of both auxotrophs. Furthermore, not only downstream genes, but also components that do not directly regulate each other can compete for some machinery in a cell.

Although our modeling dealt effects of competition for regulatory proteins and protein degradation independently, future modelings for the combinatorial effect of them would be evaluated both *in vivo *and *in scilico*. Competition for regulatory proteins *in vivo *by ChIP (Chromatin Immunoprecipitation) was modeled by computational approach [6]. Protein degradation by microscopy experiments was also evaluated by computational approach [23]. Because parameter dependency of each effect was measured for *in vivo*, combinatorial dependency will be also able to be measured in vivo. Activity landscape on such parameter space could lead us mathematical modeling for the combinatorial effect.

Our results will contribute to improvements in the field of systems and synthetic biology, because they demonstrate that considering the effect of downstream genes on regulatory genes in synthetic circuits is essential for mathematical modeling of genetic circuits.

## Methods

### Mathematical modeling of the chemical reactions and simulation

To predict the dynamic behavior of components in the gene network models, we constructed mathematical models based on biochemical reactions (e.g., the dynamics of interactions with the promoter, protein synthesis, decay of the components). Our mathematical models were based on those of Stricker *et al*. [13]. All parameters used in this work are listed in Additional File 3.

The dynamics of interaction with the promoter were determined by the following set of reactions:

$$\begin{array}{ccccc} P_{0,j}^{a,r,d}+{\text{a}}_{2} & \overset{k_{a}}{\underset{k_{-a}}{⇌}} & P_{1,j}^{a,r,d} & \text{j}\in \left\{\text{0},\text{1},\text{2}\right\} & \left(1\right)\\ P_{i,0}^{a,r,d}+{\text{r}}_{4} & \overset{2k_{r}}{\underset{k_{-r}}{⇌}} & P_{i,1}^{a,r,d} & \text{i}\in \left\{\text{0},\text{1}\right\} & \left(2\right)\\ P_{i,1}^{a,r,d}+{\text{r}}_{4} & \overset{k_{r}}{\underset{2k_{-r}}{⇌}} & P_{i,2}^{a,r,d} & \text{i}\in \left\{\text{0},\text{1}\right\} & \left(3\right)\\ \;\;P_{1,2}^{a,r,d} & \overset{k_{l}}{\underset{}{\to }} & P_{L,2}^{a,r,d}+{\text{a}}_{2} & & \left(4\right)\\ \;\;P_{0,2}^{a,r,d} & \overset{k_{l}}{\underset{}{\to }} & P_{L,2}^{a,r,d} & & \left(5\right)\\ \;\;P_{L,0}^{a,r,d} & \overset{k_{ul}}{\underset{}{\to }} & P_{0,0}^{a,r,d} & & \left(6\right) \end{array}$$

where $P_{i,j}^{a,r,d}$ represents the activator (a: AraC)/repressor (r: LacI)/downstream (d) promoters with i ∈ {0, 1} AraC dimers (a_2_) bound and j ∈ {0, 1, 2} LacI tetramers(r_4_) bound; $P_{L,j}^{a,r,d}$ is the looped state of the promoters.

The dynamics of protein synthesis were determined by the following set of reactions:

$$\begin{array}{cccc} P_{0,0}^{a,r,d} & \overset{b_{a},b_{r},b_{d}}{\to } & P_{0,0}^{a,r,d}+{\text{m}}_{\text{a/r/d}} & \left(7\right)\\ P_{1,0}^{a,r,d} & \overset{\alpha b_{a},\alpha b_{r},\alpha b_{d}}{\to } & P_{1,0}^{a,r,d}+{\text{m}}_{\text{a/r/d}} & \left(8\right)\\ \;{\text{m}}_{\text{a}} & \overset{t_{a}}{\to } & {\text{m}}_{\text{a}}+{\text{a}}_{\text{uf}}\;\; & \left(9\right)\\ \;{\text{m}}_{\text{r}} & \overset{t_{r}}{\to } & {\text{m}}_{\text{r}}+{\text{r}}_{\text{uf}}\;\; & \left(10\right)\\ \;{\text{m}}_{\text{d}} & \overset{t_{d}}{\to } & {\text{m}}_{\text{d}}+{\text{d}}_{\text{uf}}\;\; & \left(11\right)\\ \;{\text{a}}_{\text{uf}} & \overset{k_{fa}}{\to } & \text{a}\;\;\;\;\; & \left(12\right)\\ \;{\text{r}}_{\text{uf}} & \overset{k_{fr}}{\to } & \text{r}\;\;\;\;\; & \left(13\right)\\ \;{\text{d}}_{\text{uf}} & \overset{k_{fd}}{\to } & \text{d}\;\;\;\;\; & \left(14\right)\\ \text{a}+\text{a} & \overset{k_{da}}{\underset{k_{-da}}{⇌}} & {\text{a}}_{2}\;\;\;\;\; & \left(15\right)\\ \text{r}+\text{r} & \overset{k_{dr}}{\underset{k_{-dr}}{⇌}} & {\text{r}}_{\text{2}}\;\;\;\;\; & \left(16\right)\\ {\text{r}}_{2}+{\text{r}}_{2} & \overset{k_{t}}{\underset{k_{-t}}{⇌}} & {\text{r}}_{4}\;\;\;\;\; & \left(17\right) \end{array}$$

where m_a/r/d _represents the number of activators/repressors/downstream mRNAs, a_uf_, r_uf_, and d_uf _represent the number of activators/repressors/downstream unfolded polypeptides; a, r, and d represent the number of activators/repressors/downstream folded monomeric proteins; a_2 _and r_2 _represent the number of activators/repressors folded dimeric proteins; and r_4 _represents the number of repressors folded tetrameric proteins.

The dynamics of decay of the components were determined by the following set of reactions:

$$\begin{array}{ccccc} {\text{m}}_{\text{a/r/d}} & \overset{d_{a},d_{r},d_{d}}{\to } & \Phi & & \left(18\right)\\ {\text{a}}_{\text{uf}} & \overset{\lambda f\left(X\right)}{\to } & \Phi & & \left(19\right)\\ {\text{r}}_{\text{uf}} & \overset{f\left(X\right)}{\to } & \Phi & & \left(20\right)\\ {\text{d}}_{\text{uf}} & \overset{\lambda f\left(X\right)}{\to } & \Phi & & \left(21\right)\\ \text{a} & \overset{\lambda f\left(X\right)}{\to } & \Phi & & \left(22\right)\\ \text{r} & \overset{f\left(X\right)}{\to } & \Phi & & \left(23\right)\\ \text{d} & \overset{\lambda f\left(X\right)}{\to } & \Phi & & \left(24\right)\\ {\text{a}}_{2} & \overset{\lambda f\left(X\right)}{\to } & \Phi & & \left(25\right)\\ {\text{r}}_{2} & \overset{f\left(X\right)}{\to } & \Phi & & \left(26\right)\\ {\text{r}}_{4} & \overset{f\left(X\right)}{\to } & \Phi & & \left(27\right)\\ P_{1,j}^{a,r,d} & \overset{\alpha f\left(X\right)}{\to } & P_{0,j}^{a,r,d} & \text{j}\in \left\{0,1,2\right\} & \left(28\right)\\ P_{i,1}^{a,r,d} & \overset{f\left(X\right)}{\to } & P_{i,0}^{a,r,d} & \text{j}\in \left\{0,1\right\} & \left(29\right)\\ P_{i,2}^{a,r,d} & \overset{2f\left(X\right)}{\to } & P_{i,1}^{a,r,d} & \text{j}\in \left\{0,1\right\} & \left(30\right)\\ P_{L,2}^{a,r,d} & \overset{2\varepsilon f\left(X\right)}{\to } & P_{L,1}^{a,r,d} & & \left(31\right)\\ P_{L,1}^{a,r,d} & \overset{\varepsilon f\left(X\right)}{\to } & P_{L,0}^{a,r,d} & & \left(32\right) \end{array}$$

where

$$\begin{array}{cccc} X & = & {\text{a}}_{\text{uf}}+{\text{r}}_{\text{uf}}+{\text{d}}_{\text{uf}}+\text{a}+\text{r}+\text{d}+2{\text{r}}_{2}+2{\text{a}}_{2}+4{\text{r}}_{4} & \\ & & +4\left(P_{0,1}^{a}+P_{0,1}^{r}+P_{0,1}^{d}\right)+8\left(P_{0,2}^{a}+P_{0,2}^{r}+P_{0,2}^{d}\right) & \\ & & +2\left(P_{1,0}^{a}+P_{1,0}^{r}+P_{1,0}^{d}\right)+6\left(P_{1,1}^{a}+P_{1,1}^{r}+P_{1,1}^{d}\right)+10\left(P_{1,2}^{a}+P_{1,2}^{r}+P_{1,2}^{d}\right) & \\ & & +8\left(P_{L,2}^{a}+P_{L,2}^{r}+P_{L,2}^{d}\right)+4\left(P_{L,1}^{a}+P_{L,1}^{r}+P_{L,1}^{d}\right) & \left(33\right)\\ f\left(X\right) & = & \frac{\gamma }{c_{e}+f\left(X\right)} & \left(34\right)\\ \;\;{\text{k}}_{\text{r}} & = & {\text{k}}_{\text{ - r}}\left(\left(C_{r}^{max}-C_{r}^{min}\right)\frac{1}{1+{\left(\frac{\left[IPTG\right]}{k_{r1}}\right)}^{b_{1}}}+C_{r}^{min}\right) & \left(35\right)\\ \;\;{\text{k}}_{\text{a}} & = & {\text{k}}_{-\text{a}}\left(\left(C_{a}^{max}-C_{a}^{min}\right)\frac{{\left[ara\right]}^{c_{1}}}{k_{a1}^{c_{1}}+{\left[ara\right]}^{c_{1}}}\frac{1}{1+{\left(\frac{\left[IPTG\right]}{k_{r1}}\right)}^{b_{1}}}+C_{a}^{min}\right) & \left(36\right) \end{array}$$

and copy number variations are accounted for as follows:

$$\begin{array}{cc} P_{L,0}^{a}={\text{N}}_{\text{a}}-\left(P_{0,0}^{a}+P_{0,1}^{a}+P_{0,2}^{a}+P_{1,0}^{a}+P_{1,1}^{a}+P_{1,2}^{a}+P_{L,1}^{a}+P_{L,2}^{a}\right) & \left(37\right)\\ P_{L,0}^{r}={\text{N}}_{\text{r}}-\left(P_{0,0}^{r}+P_{0,1}^{r}+P_{0,2}^{r}+P_{1,0}^{r}+P_{1,1}^{r}+P_{1,2}^{r}+P_{L,1}^{r}+P_{L,2}^{r}\right) & \left(38\right)\\ P_{L,0}^{d}={\text{N}}_{\text{d}}-\left(P_{0,0}^{d}+P_{0,1}^{d}+P_{0,2}^{d}+P_{1,0}^{d}+P_{1,1}^{d}+P_{1,2}^{d}+P_{L,1}^{d}+P_{L,2}^{d}\right) & \left(39\right) \end{array}$$

The deterministic model was simulated using MATLAB software developed by MathWorks. Our simulation ODEs were performed by ode45 solver in MATLAB. Initial values in the reporter-less model are configured in [$P_{0,0}^{a}$, $P_{0,1}^{a}$, $P_{0,2}^{a}$, $P_{1,0}^{a}$, $P_{1,1}^{a}$, $P_{1,2}^{a}$, $P_{L,1}^{a}$, $P_{L,2}^{a}$, $P_{0,0}^{r}$, $P_{0,1}^{r}$, $P_{0,2}^{r}$, $P_{1,0}^{r}$, $P_{1,1}^{r}$, $P_{1,2}^{r}$, $P_{L,1}^{r}$, $P_{L,2}^{r}$, m_a_, m_r_, a_uf_, r_uf_, a, r, a_2_, r_2_, r_4 _= N_a_, 0, 0, 0, 0, 0, 0, 0, N_r_, 0, 0, 0, 0, 0, 0, 0, 0, 0, 0, 0, 0, 0, 0, 0, 0 (molecules)]. Initial values in the reporter-containing model are configured in [$P_{0,0}^{a}$, $P_{0,1}^{a}$, $P_{0,2}^{a}$, $P_{1,0}^{a}$, $P_{1,1}^{a}$, $P_{1,2}^{a}$, $P_{L,1}^{a}$, $P_{L,2}^{a}$, $P_{0,0}^{r}$, $P_{0,1}^{r}$, $P_{0,2}^{r}$, $P_{1,0}^{r}$, $P_{1,1}^{r}$, $P_{1,2}^{r}$, $P_{L,1}^{r}$, $P_{L,2}^{r}$, $P_{0,0}^{d}$, $P_{0,1}^{d}$, $P_{0,2}^{d}$, $P_{1,0}^{d}$, $P_{1,1}^{d}$, $P_{1,2}^{d}$, $P_{L,1}^{d}$, $P_{L,2}^{d}$, m_a_, m_r_, m_d_, a_uf_, r_uf_, d_uf_, a, r, d, a_2_, r_2_, r_4 _= N_a_, 0, 0, 0, 0, 0, 0, 0, N_r_, 0, 0, 0, 0, 0, 0, 0, N_d_, 0, 0, 0, 0, 0, 0, 0, 0, 0, 0, 0, 0, 0, 0, 0, 0, 0, 0, 0 (molecules)].

## Competing interests

The authors declare that they have no competing interests.

## Authors' contributions

TM performed the mathematical modeling and simulation. TM, MY, and DK designed the study. All authors wrote and revised the manuscript together. All authors read and approved the final manuscript.

## Supplementary Material

Additional File 1 **Oscillation time-course of the reporter-containing model (Nd = 50) at 0**.0% arabinose and 10 mM IPTG concentration. AraC dimer protein (blue), LacI tetramer protein (green), and GFP monomer protein (red). Click here for file

Additional File 2 **AraC dimer oscillation time-course JPEG-format file of the two models at each arabinose and IPTG concentration**. The reporter-less model is shown in blue and the reporter-containing model (Nd = 50) in red. Click here for file

Additional File 3 **Parameters used for the simulations**. Click here for file

## Declarations

Publication of this article is supported by the program for promoting the enhancement of research universities, MEXT, Japan.

This article has been published as part of *BMC Systems Biology *Volume 8 Supplement 4, 2014: Thirteenth International Conference on Bioinformatics (InCoB2014): Systems Biology. The full contents of the supplement are available online at http://www.biomedcentral.com/bmcsystbiol/supplements/8/S4.
