# Supplementary material for: Effects of downstream genes on synthetic genetic circuits
Source: BMC Syst Biol. 2014 Dec 8;8(Suppl 4):S4. doi: 10.1186/1752-0509-8-S4-S4 (PMC4290693; doi:10.1186/1752-0509-8-S4-S4)
Supplement: Additional File 1 — Oscillation time-course of the reporter-containing model (Nd = 50) at 0.0% arabinose and 10 mM IPTG concentration. AraC dimer protein (blue), LacI tetramer protein (green), and GFP monomer protein (red). [file 1752-0509-8-S4-S4-S1.pdf]

(Additional File 1) Reporter-containing model

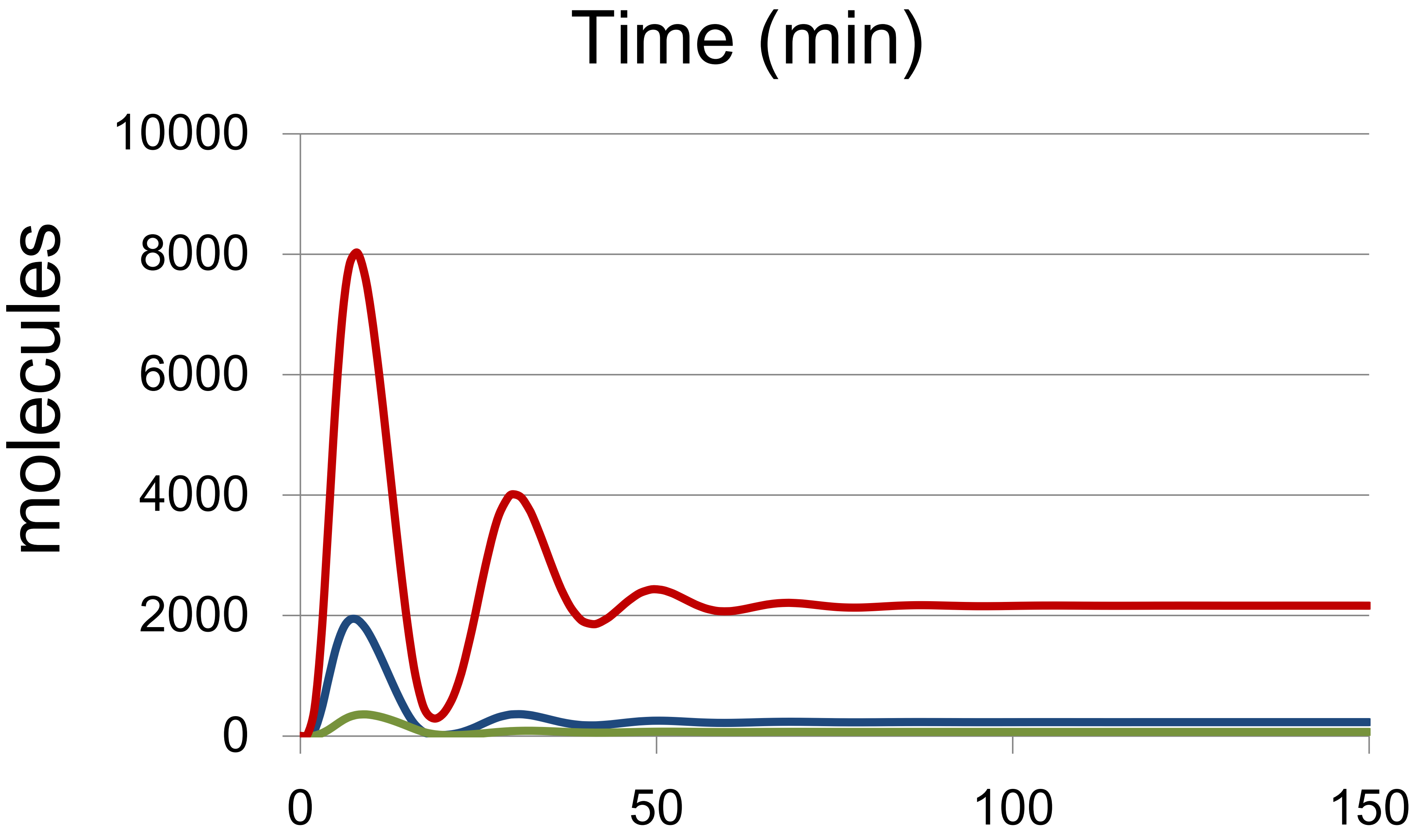

This time course was performed at 0.0% arabinose and 10 mM IPTG.
