# Supplementary figures and images for: Effects of downstream genes on synthetic genetic circuits
Source: BMC Syst Biol. 2014 Dec 8;8(Suppl 4):S4. doi: 10.1186/1752-0509-8-S4-S4 (PMC4290693; doi:10.1186/1752-0509-8-S4-S4)

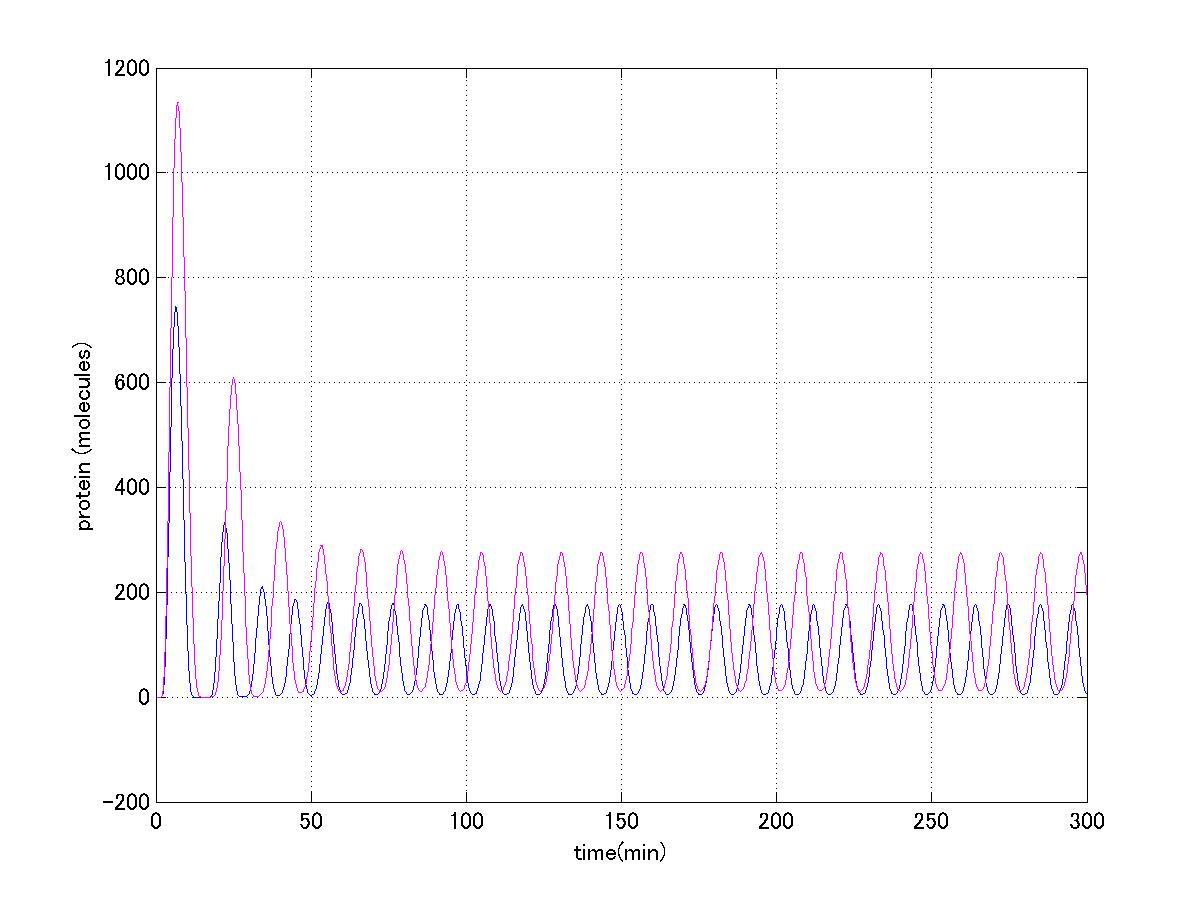

Supplement: Additional File 2 — AraC dimer oscillation time-course JPEG-format file of the two models at each arabinose and IPTG concentration. The reporter-less model is shown in blue and the reporter-containing model (Nd = 50) in red. [file 1752-0509-8-S4-S4-S2.zip › 1752-0509-8-S3-S5-S2/AraC_time_course_arabinose0.039811%_IPTG0.12589mM.jpg]

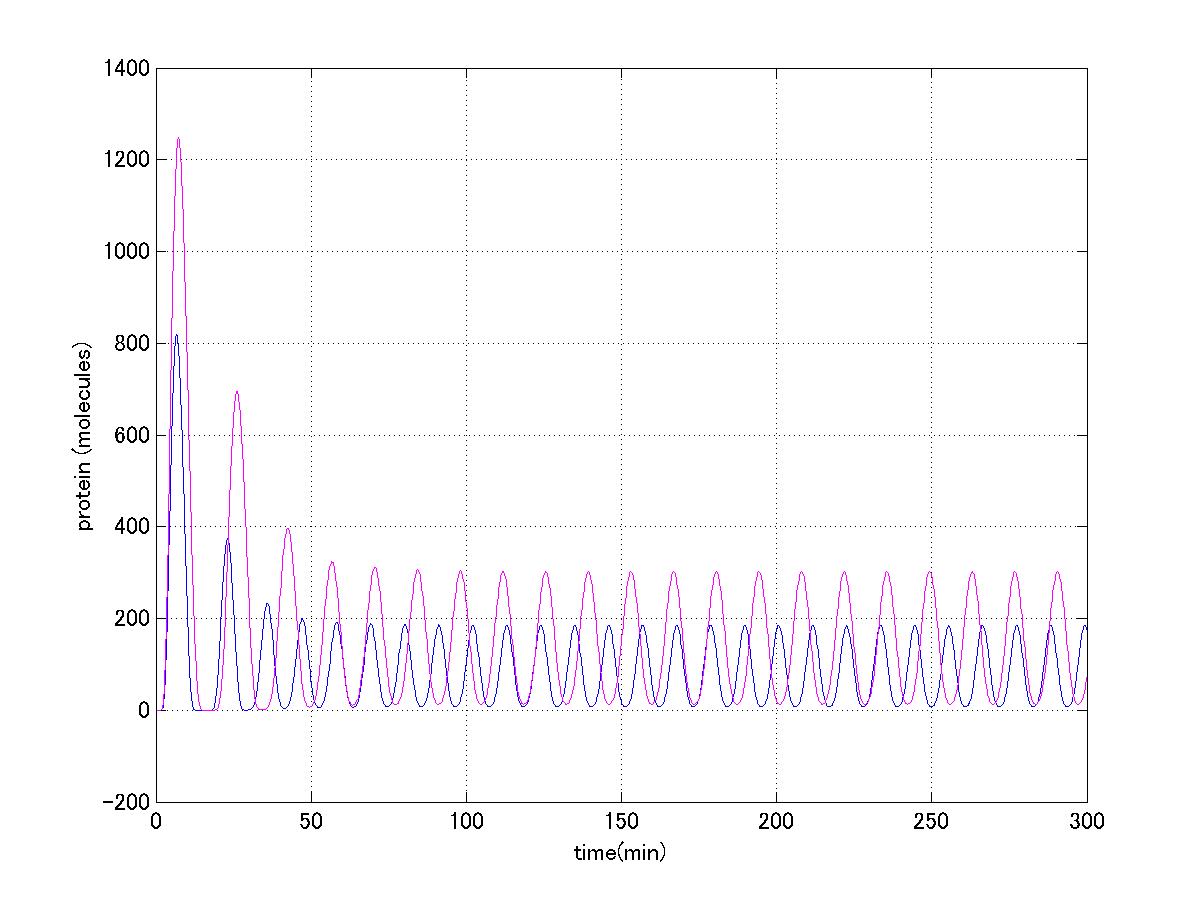

Supplement: Additional File 2 — AraC dimer oscillation time-course JPEG-format file of the two models at each arabinose and IPTG concentration. The reporter-less model is shown in blue and the reporter-containing model (Nd = 50) in red. [file 1752-0509-8-S4-S4-S2.zip › 1752-0509-8-S3-S5-S2/AraC_time_course_arabinose0.039811%_IPTG0.15849mM.jpg]

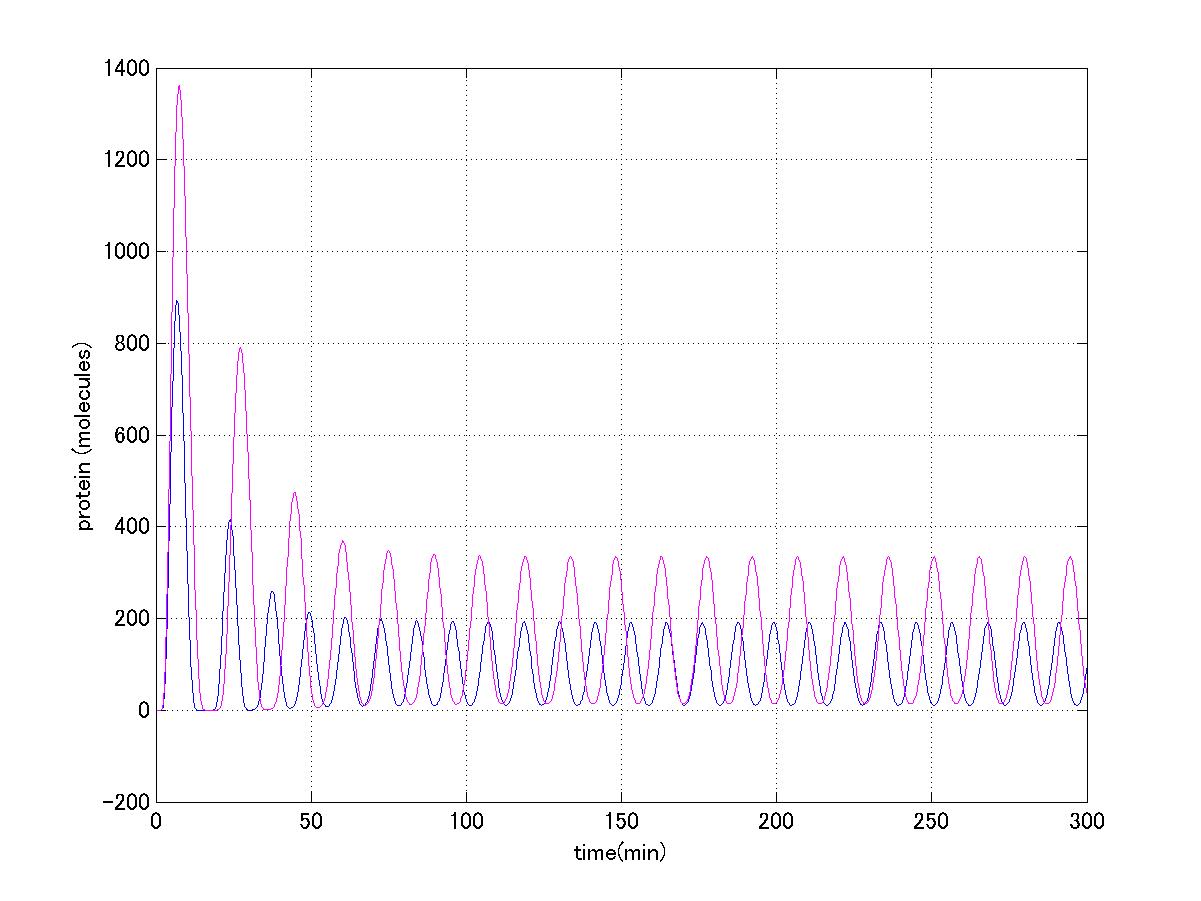

Supplement: Additional File 2 — AraC dimer oscillation time-course JPEG-format file of the two models at each arabinose and IPTG concentration. The reporter-less model is shown in blue and the reporter-containing model (Nd = 50) in red. [file 1752-0509-8-S4-S4-S2.zip › 1752-0509-8-S3-S5-S2/AraC_time_course_arabinose0.039811%_IPTG0.19953mM.jpg]

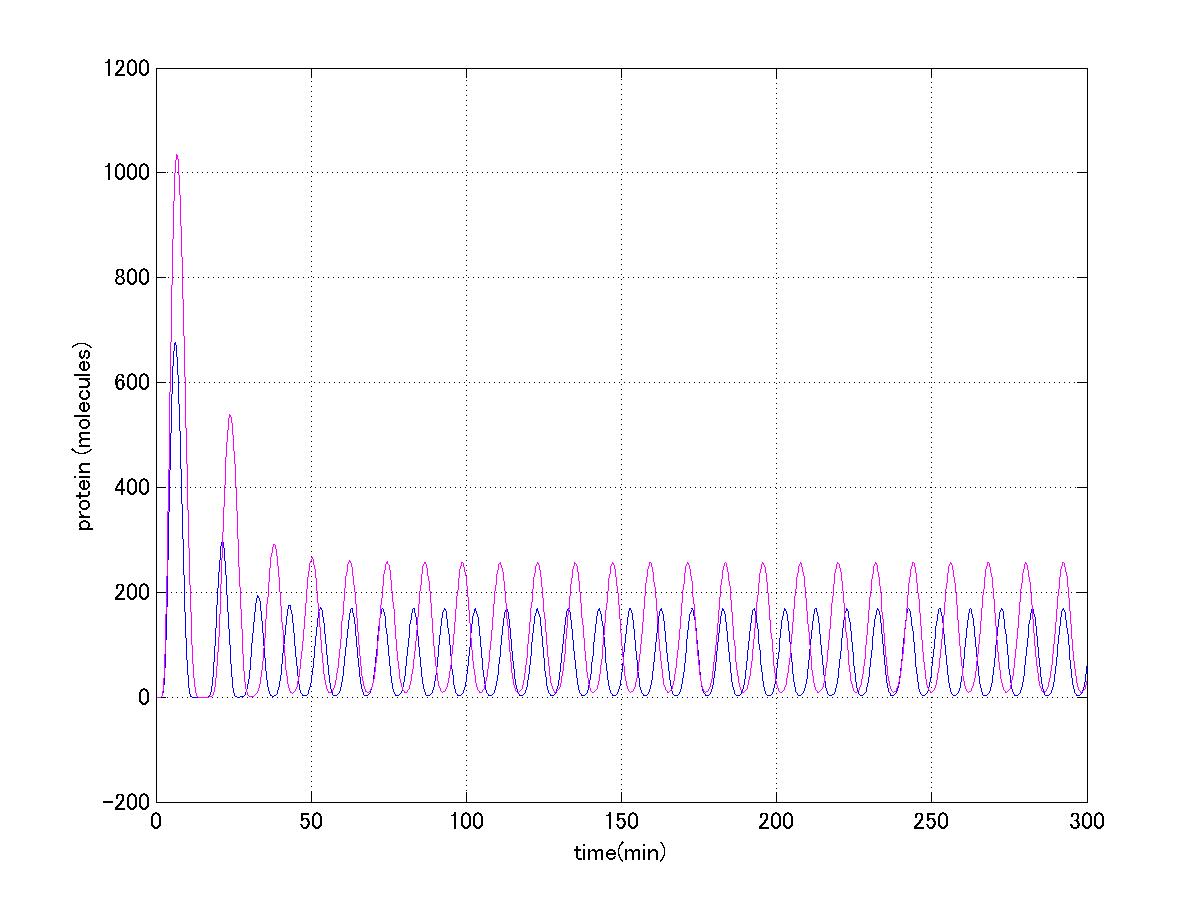

Supplement: Additional File 2 — AraC dimer oscillation time-course JPEG-format file of the two models at each arabinose and IPTG concentration. The reporter-less model is shown in blue and the reporter-containing model (Nd = 50) in red. [file 1752-0509-8-S4-S4-S2.zip › 1752-0509-8-S3-S5-S2/AraC_time_course_arabinose0.039811%_IPTG0.1mM.jpg]

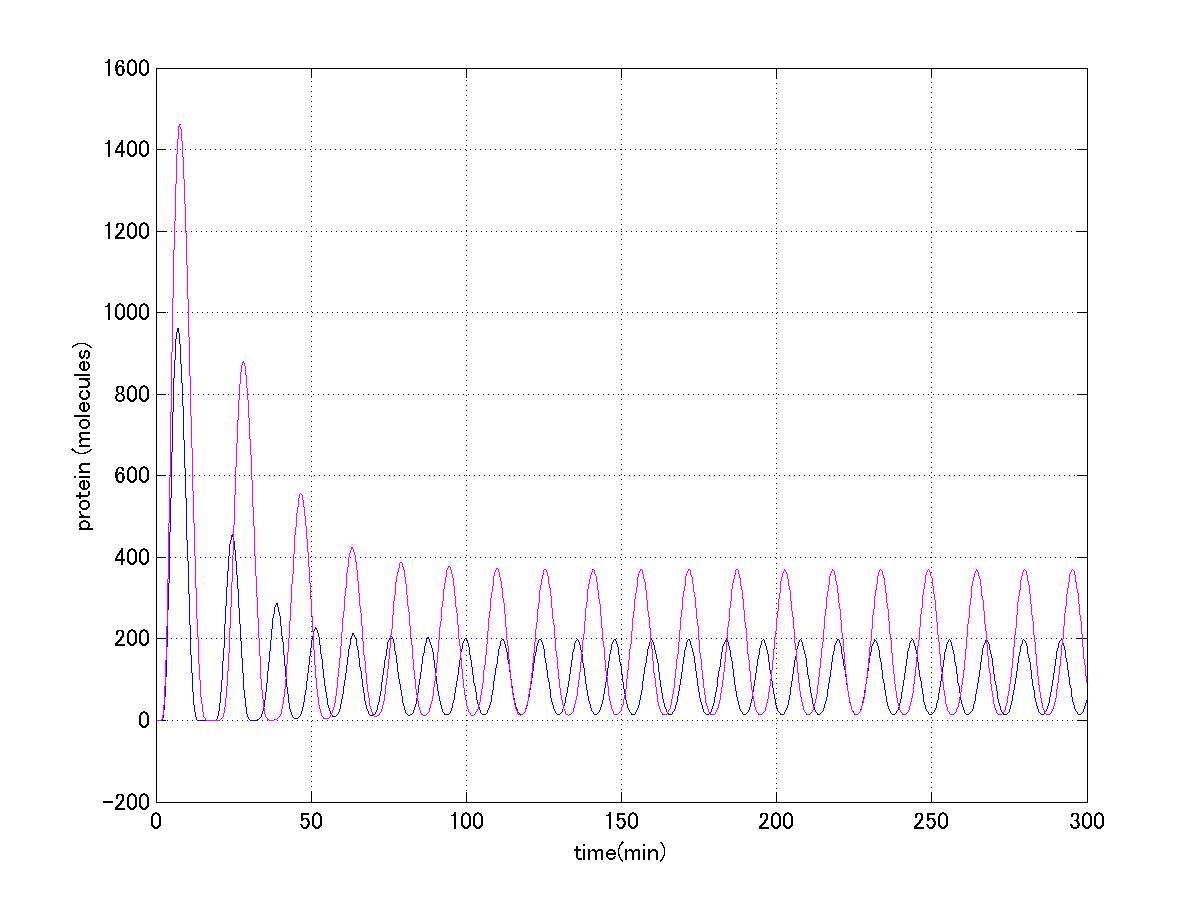

Supplement: Additional File 2 — AraC dimer oscillation time-course JPEG-format file of the two models at each arabinose and IPTG concentration. The reporter-less model is shown in blue and the reporter-containing model (Nd = 50) in red. [file 1752-0509-8-S4-S4-S2.zip › 1752-0509-8-S3-S5-S2/AraC_time_course_arabinose0.039811%_IPTG0.25119mM.jpg]

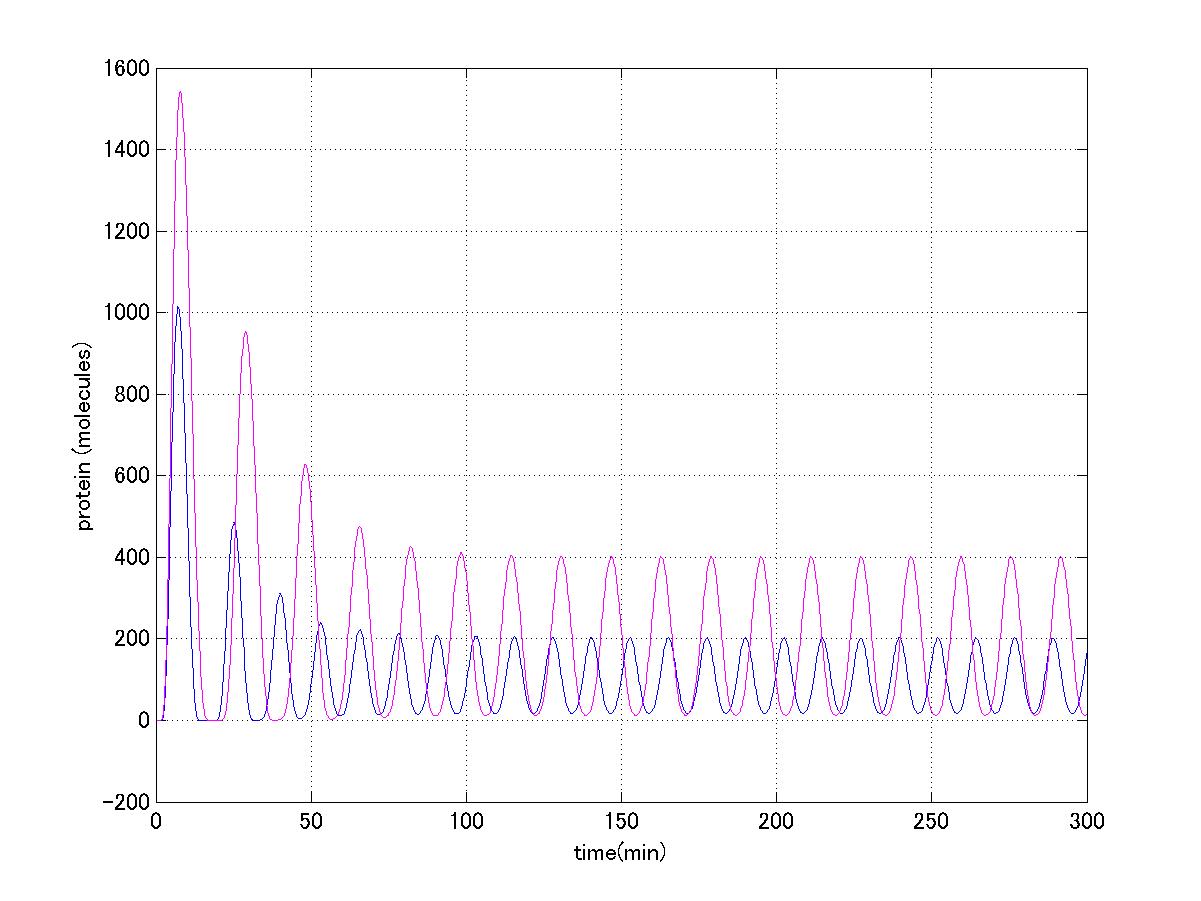

Supplement: Additional File 2 — AraC dimer oscillation time-course JPEG-format file of the two models at each arabinose and IPTG concentration. The reporter-less model is shown in blue and the reporter-containing model (Nd = 50) in red. [file 1752-0509-8-S4-S4-S2.zip › 1752-0509-8-S3-S5-S2/AraC_time_course_arabinose0.039811%_IPTG0.31623mM.jpg]

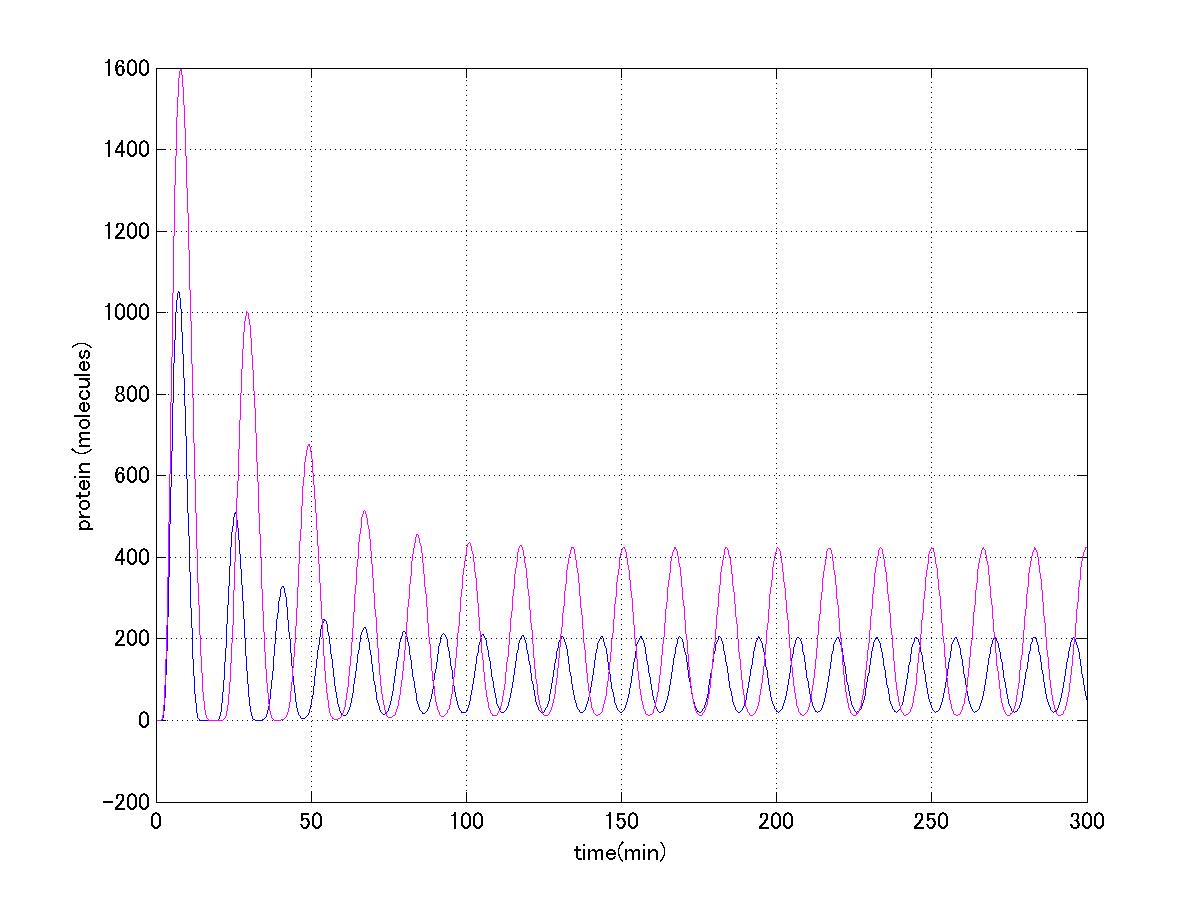

Supplement: Additional File 2 — AraC dimer oscillation time-course JPEG-format file of the two models at each arabinose and IPTG concentration. The reporter-less model is shown in blue and the reporter-containing model (Nd = 50) in red. [file 1752-0509-8-S4-S4-S2.zip › 1752-0509-8-S3-S5-S2/AraC_time_course_arabinose0.039811%_IPTG0.39811mM.jpg]

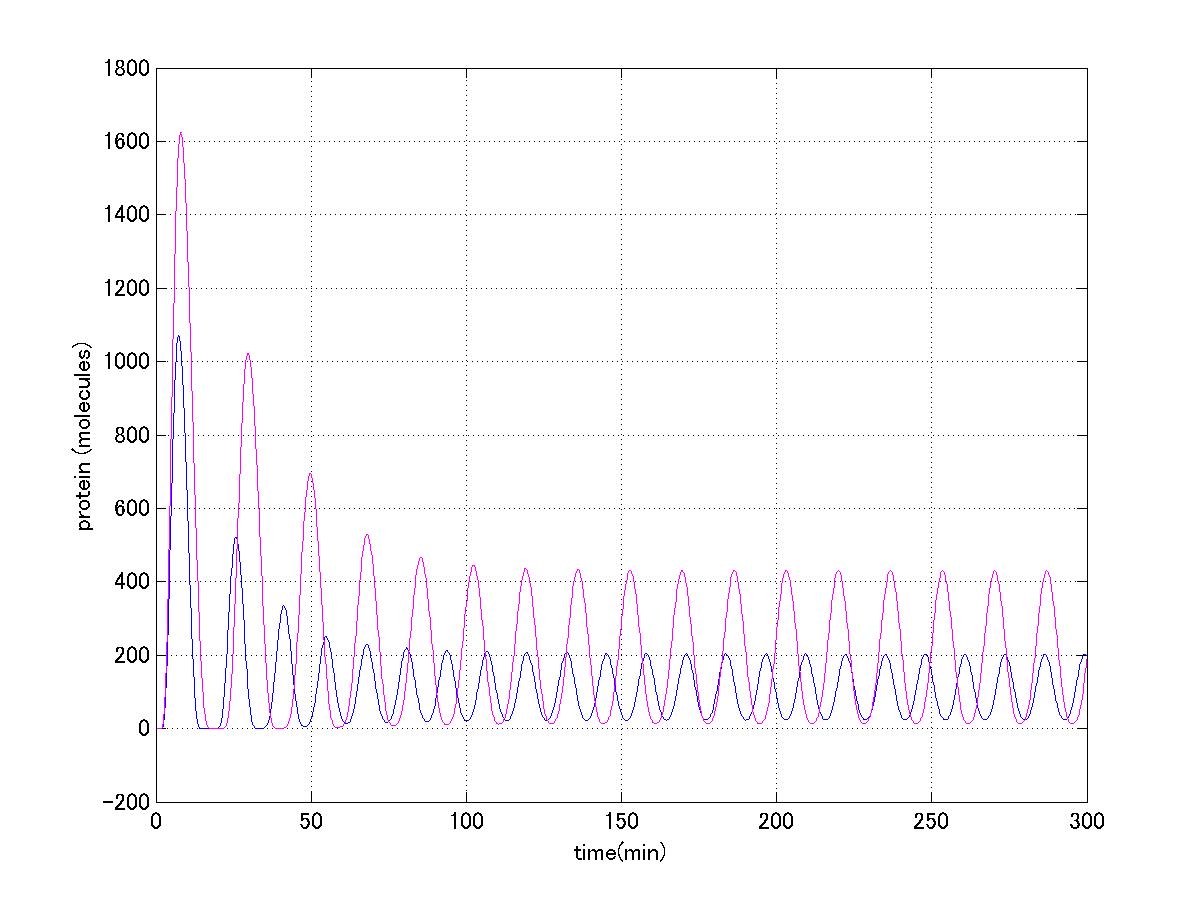

Supplement: Additional File 2 — AraC dimer oscillation time-course JPEG-format file of the two models at each arabinose and IPTG concentration. The reporter-less model is shown in blue and the reporter-containing model (Nd = 50) in red. [file 1752-0509-8-S4-S4-S2.zip › 1752-0509-8-S3-S5-S2/AraC_time_course_arabinose0.039811%_IPTG0.50119mM.jpg]

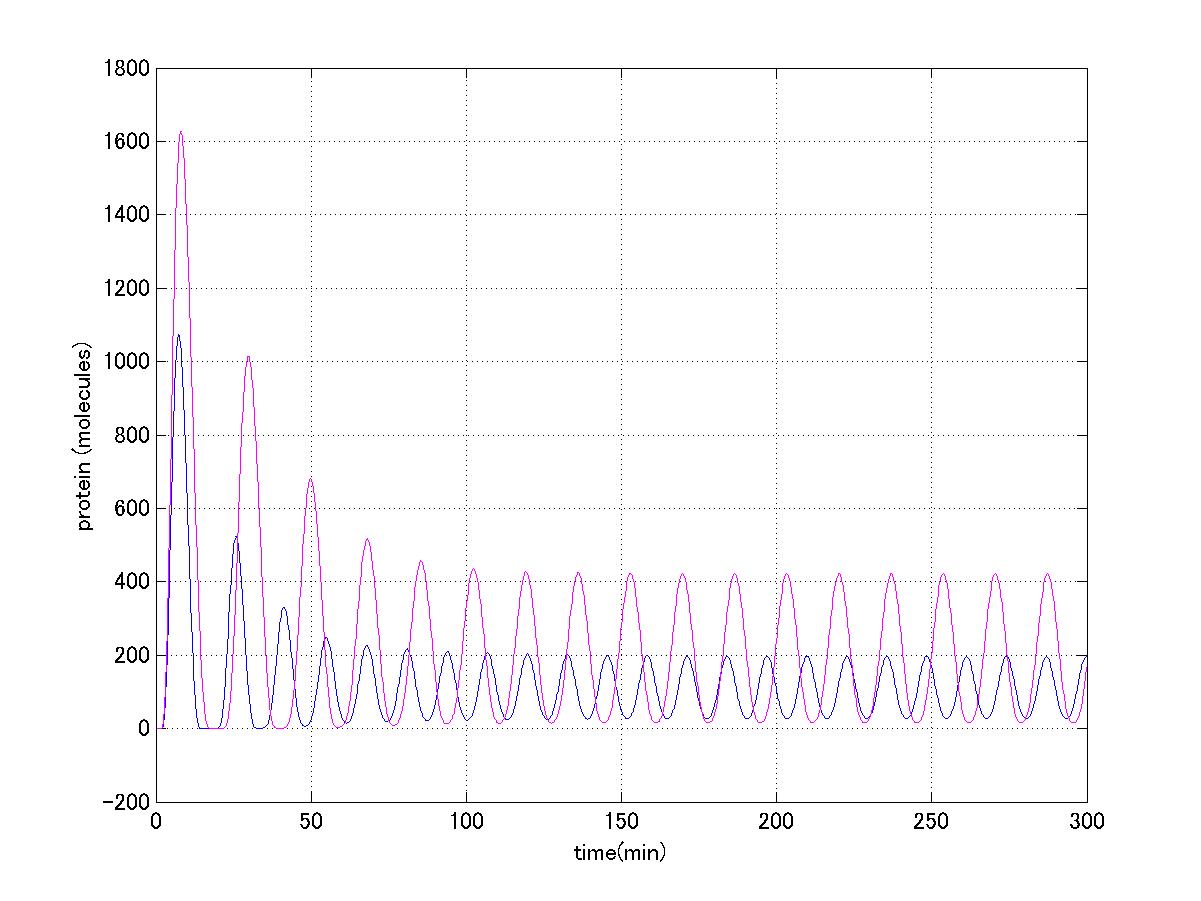

Supplement: Additional File 2 — AraC dimer oscillation time-course JPEG-format file of the two models at each arabinose and IPTG concentration. The reporter-less model is shown in blue and the reporter-containing model (Nd = 50) in red. [file 1752-0509-8-S4-S4-S2.zip › 1752-0509-8-S3-S5-S2/AraC_time_course_arabinose0.039811%_IPTG0.63096mM.jpg]

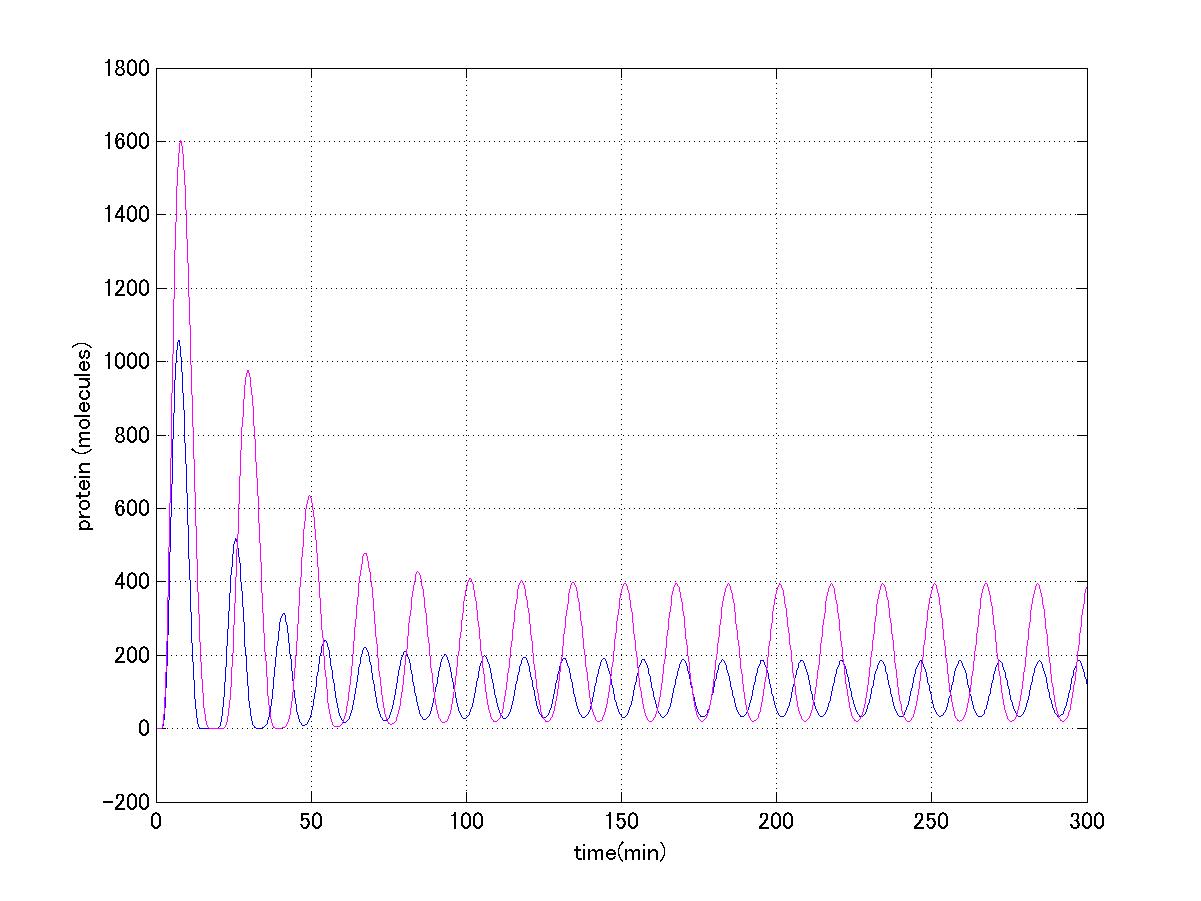

Supplement: Additional File 2 — AraC dimer oscillation time-course JPEG-format file of the two models at each arabinose and IPTG concentration. The reporter-less model is shown in blue and the reporter-containing model (Nd = 50) in red. [file 1752-0509-8-S4-S4-S2.zip › 1752-0509-8-S3-S5-S2/AraC_time_course_arabinose0.039811%_IPTG0.79433mM.jpg]

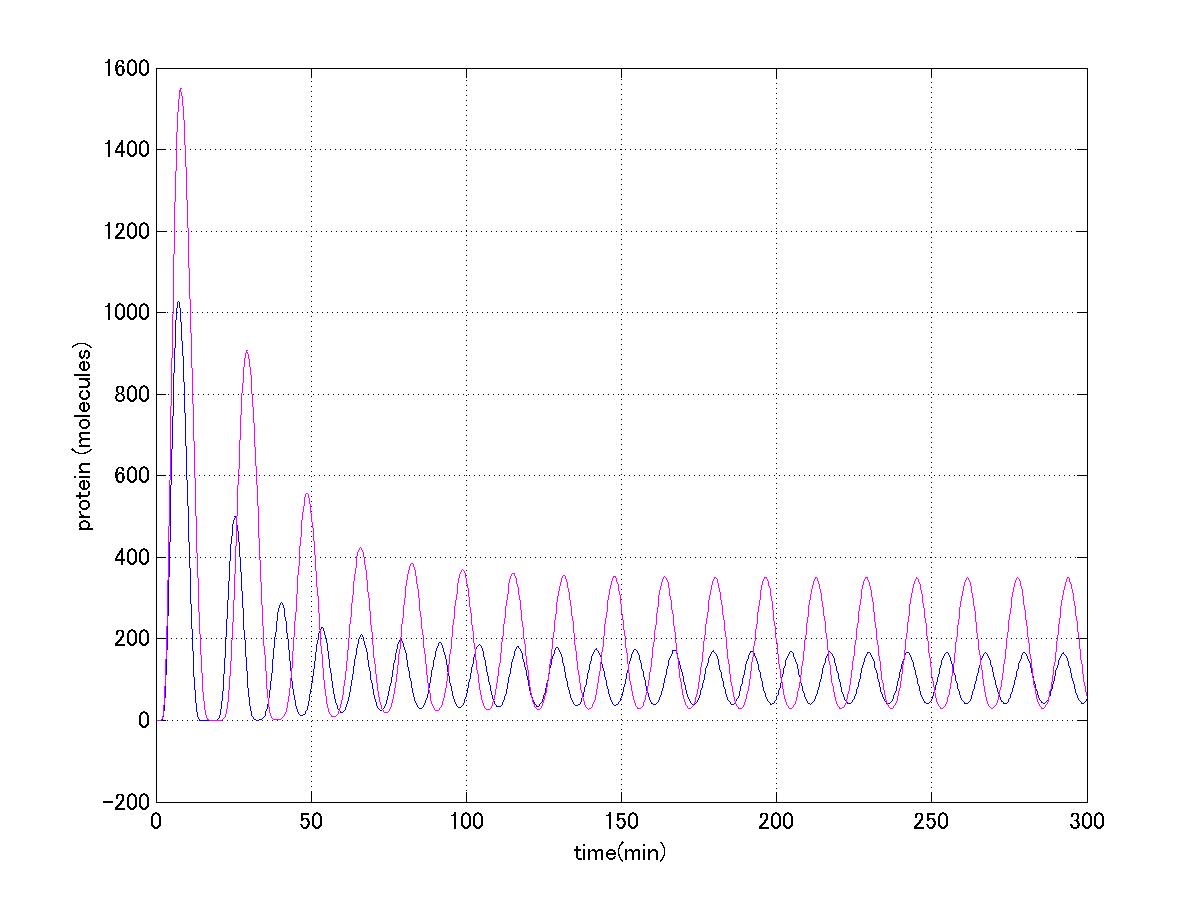

Supplement: Additional File 2 — AraC dimer oscillation time-course JPEG-format file of the two models at each arabinose and IPTG concentration. The reporter-less model is shown in blue and the reporter-containing model (Nd = 50) in red. [file 1752-0509-8-S4-S4-S2.zip › 1752-0509-8-S3-S5-S2/AraC_time_course_arabinose0.039811%_IPTG1mM.jpg]

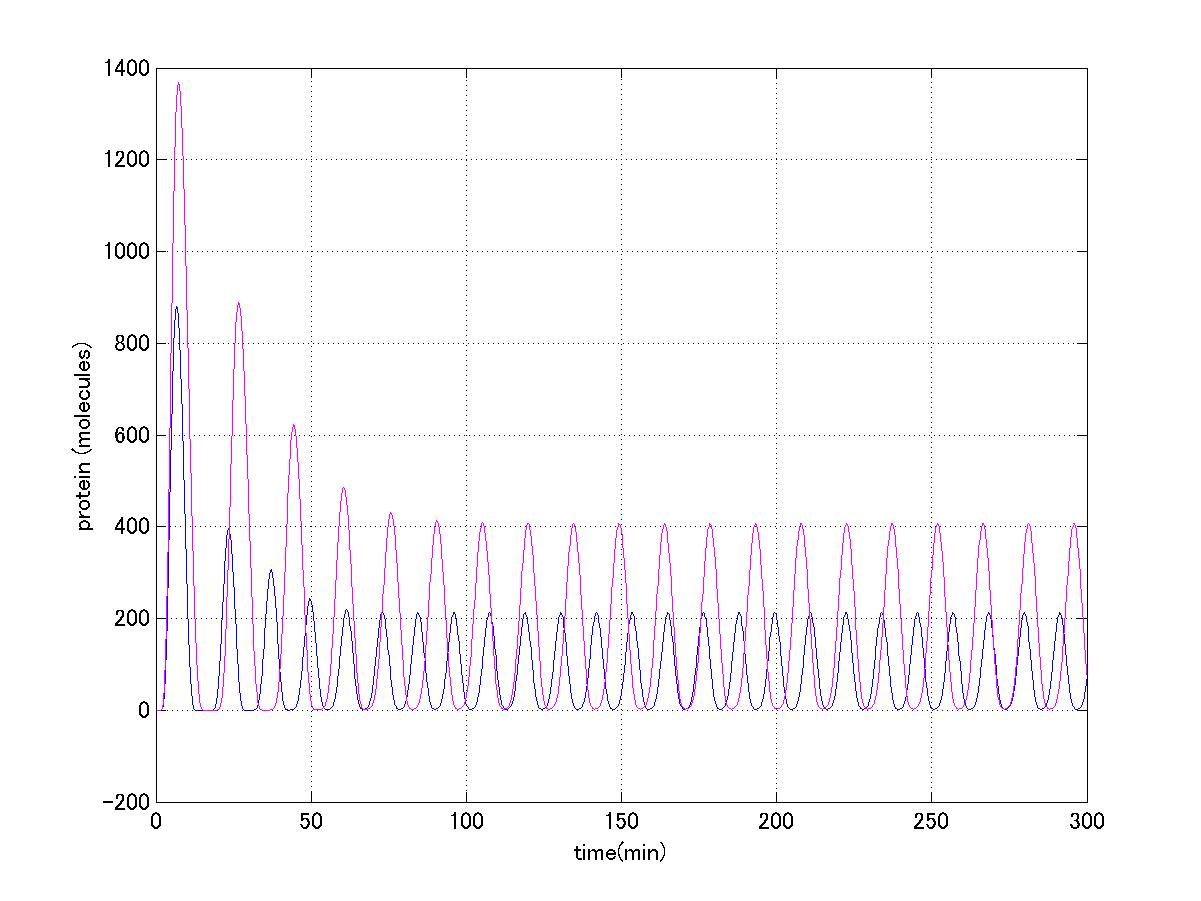

Supplement: Additional File 2 — AraC dimer oscillation time-course JPEG-format file of the two models at each arabinose and IPTG concentration. The reporter-less model is shown in blue and the reporter-containing model (Nd = 50) in red. [file 1752-0509-8-S4-S4-S2.zip › 1752-0509-8-S3-S5-S2/AraC_time_course_arabinose0.050119%_IPTG0.12589mM.jpg]

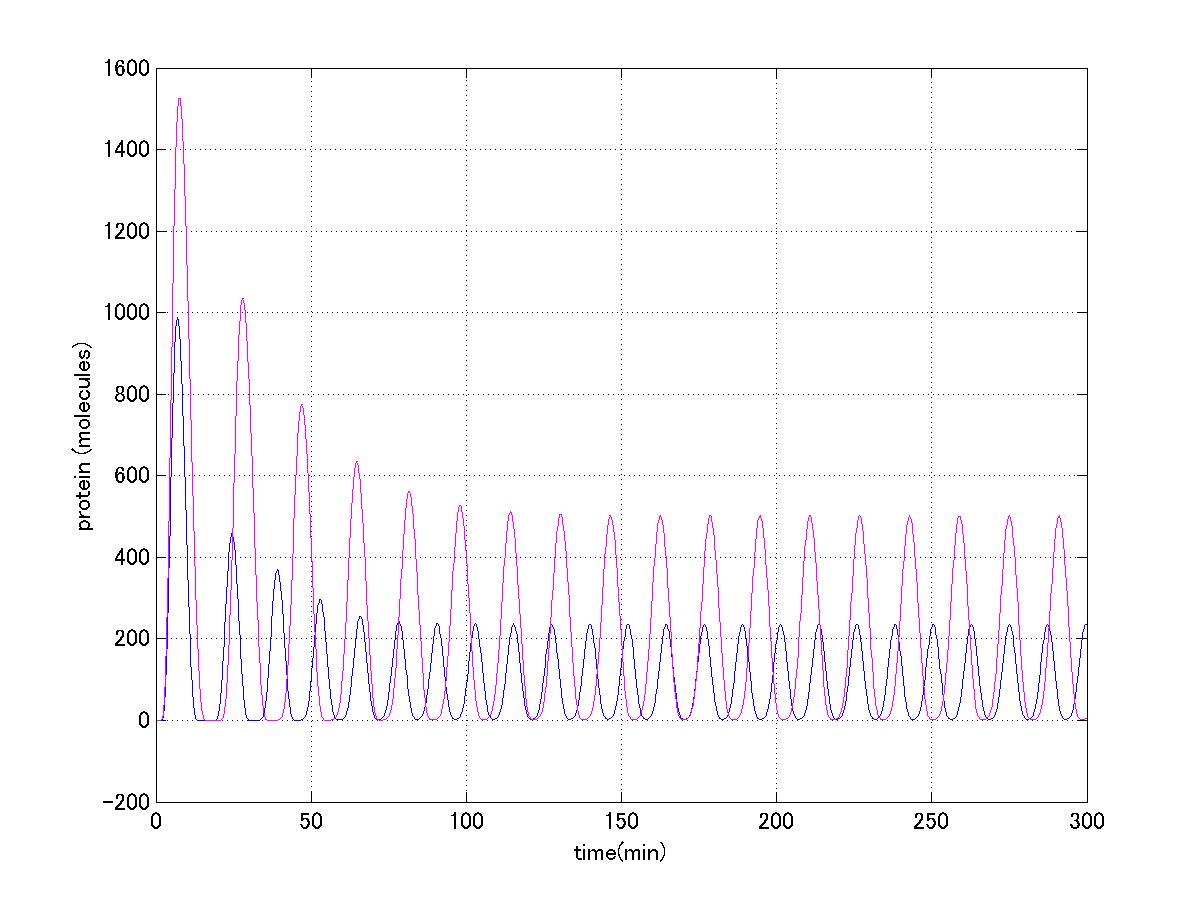

Supplement: Additional File 2 — AraC dimer oscillation time-course JPEG-format file of the two models at each arabinose and IPTG concentration. The reporter-less model is shown in blue and the reporter-containing model (Nd = 50) in red. [file 1752-0509-8-S4-S4-S2.zip › 1752-0509-8-S3-S5-S2/AraC_time_course_arabinose0.050119%_IPTG0.15849mM.jpg]

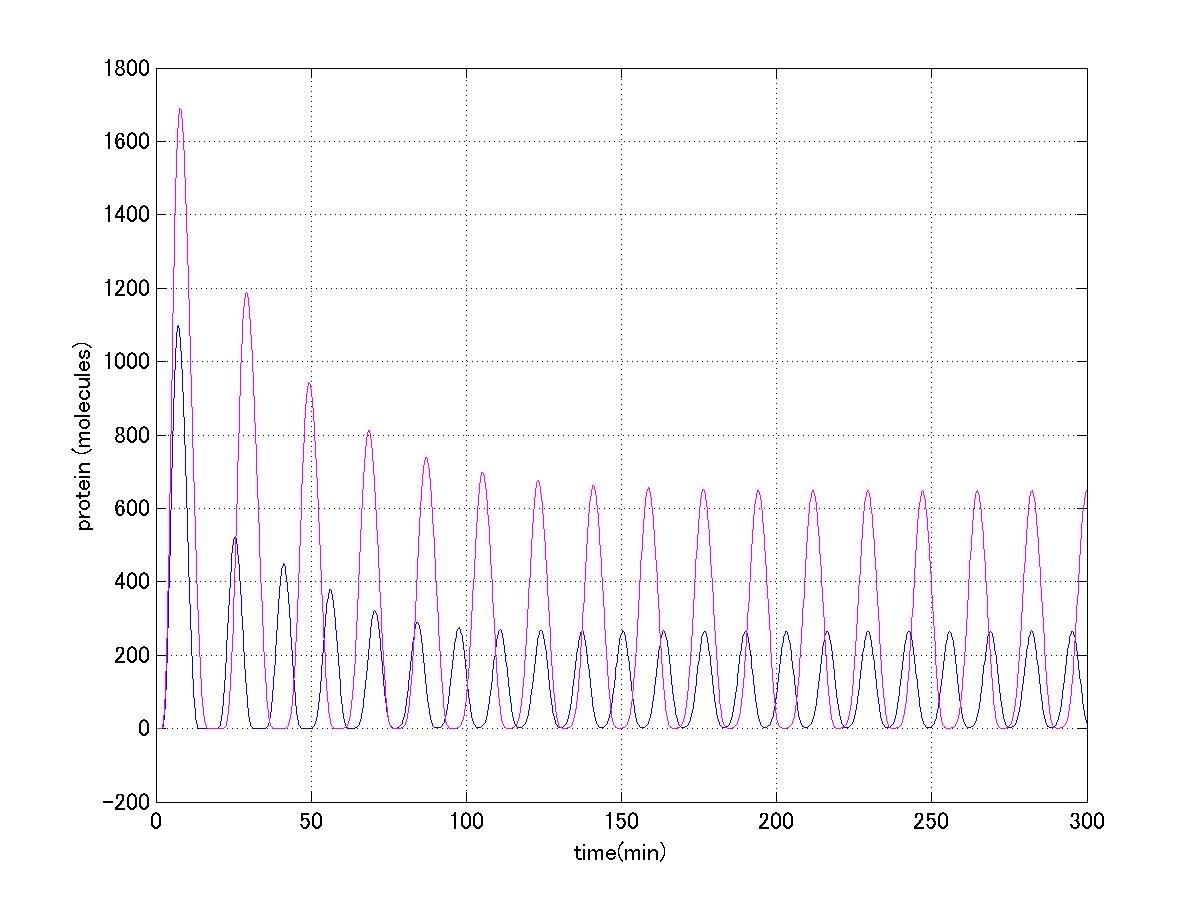

Supplement: Additional File 2 — AraC dimer oscillation time-course JPEG-format file of the two models at each arabinose and IPTG concentration. The reporter-less model is shown in blue and the reporter-containing model (Nd = 50) in red. [file 1752-0509-8-S4-S4-S2.zip › 1752-0509-8-S3-S5-S2/AraC_time_course_arabinose0.050119%_IPTG0.19953mM.jpg]

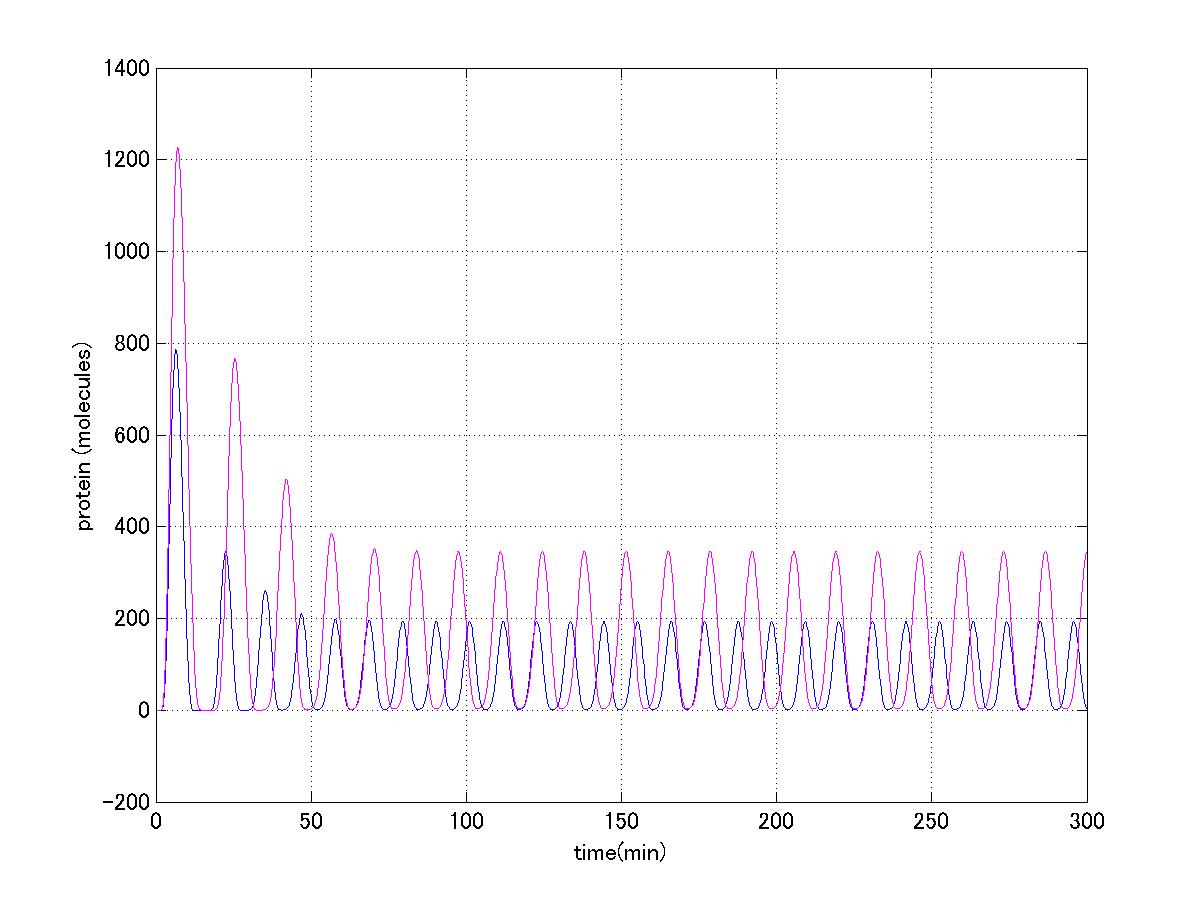

Supplement: Additional File 2 — AraC dimer oscillation time-course JPEG-format file of the two models at each arabinose and IPTG concentration. The reporter-less model is shown in blue and the reporter-containing model (Nd = 50) in red. [file 1752-0509-8-S4-S4-S2.zip › 1752-0509-8-S3-S5-S2/AraC_time_course_arabinose0.050119%_IPTG0.1mM.jpg]

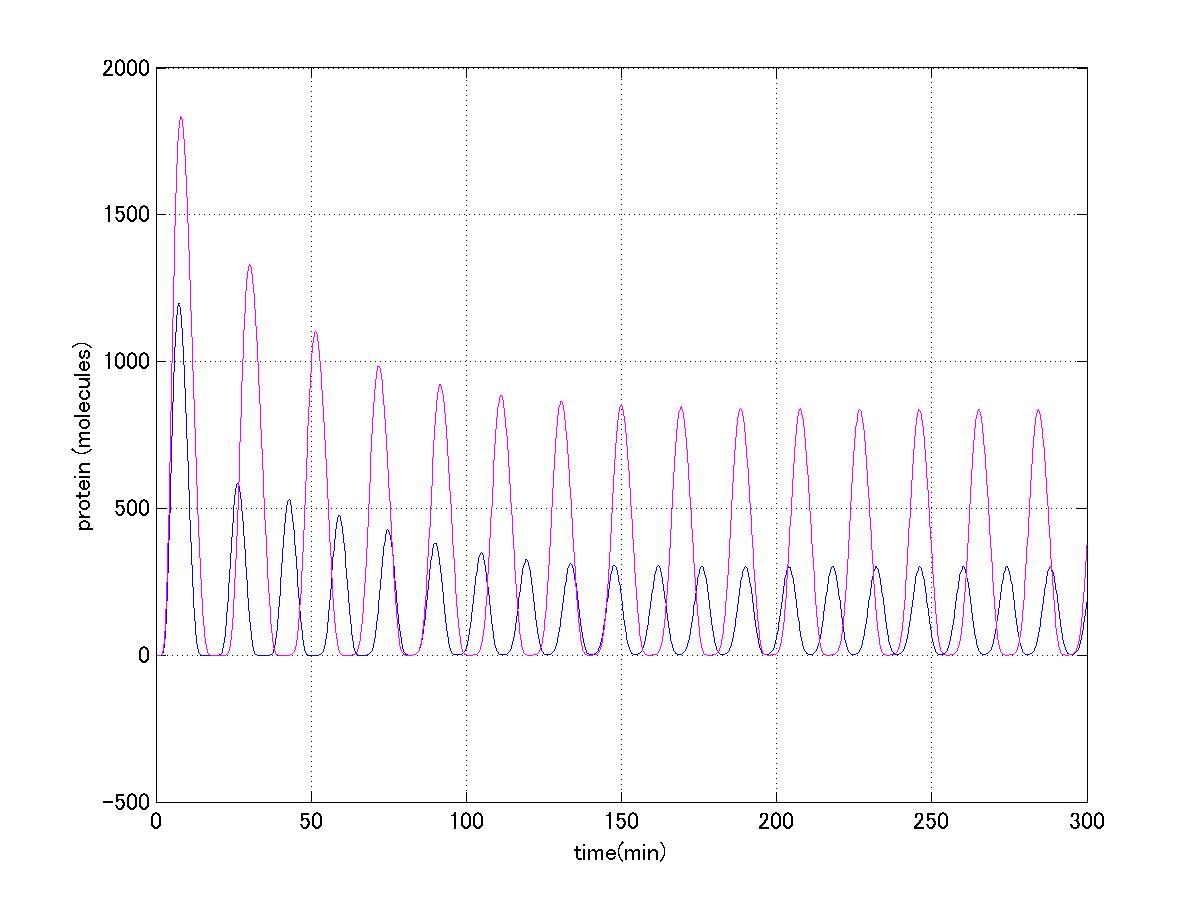

Supplement: Additional File 2 — AraC dimer oscillation time-course JPEG-format file of the two models at each arabinose and IPTG concentration. The reporter-less model is shown in blue and the reporter-containing model (Nd = 50) in red. [file 1752-0509-8-S4-S4-S2.zip › 1752-0509-8-S3-S5-S2/AraC_time_course_arabinose0.050119%_IPTG0.25119mM.jpg]

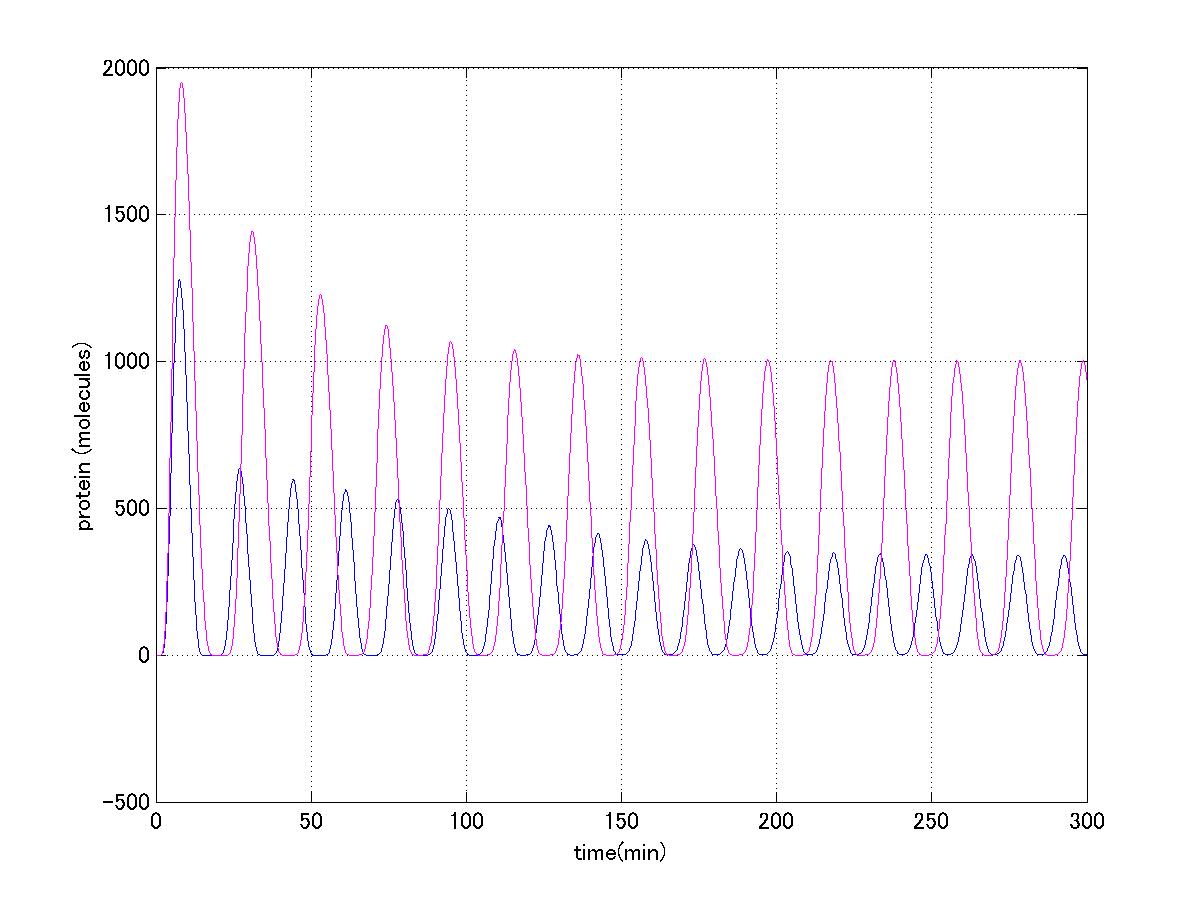

Supplement: Additional File 2 — AraC dimer oscillation time-course JPEG-format file of the two models at each arabinose and IPTG concentration. The reporter-less model is shown in blue and the reporter-containing model (Nd = 50) in red. [file 1752-0509-8-S4-S4-S2.zip › 1752-0509-8-S3-S5-S2/AraC_time_course_arabinose0.050119%_IPTG0.31623mM.jpg]

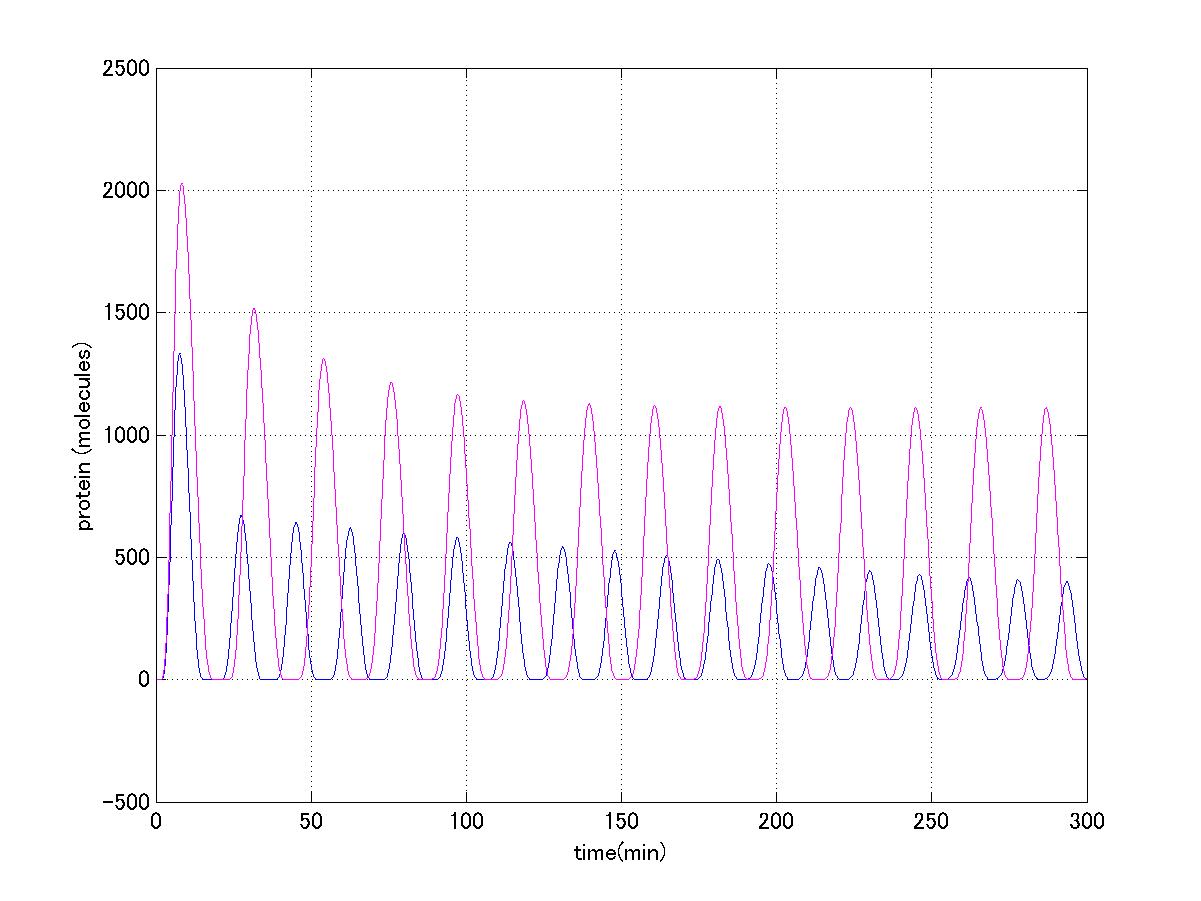

Supplement: Additional File 2 — AraC dimer oscillation time-course JPEG-format file of the two models at each arabinose and IPTG concentration. The reporter-less model is shown in blue and the reporter-containing model (Nd = 50) in red. [file 1752-0509-8-S4-S4-S2.zip › 1752-0509-8-S3-S5-S2/AraC_time_course_arabinose0.050119%_IPTG0.39811mM.jpg]

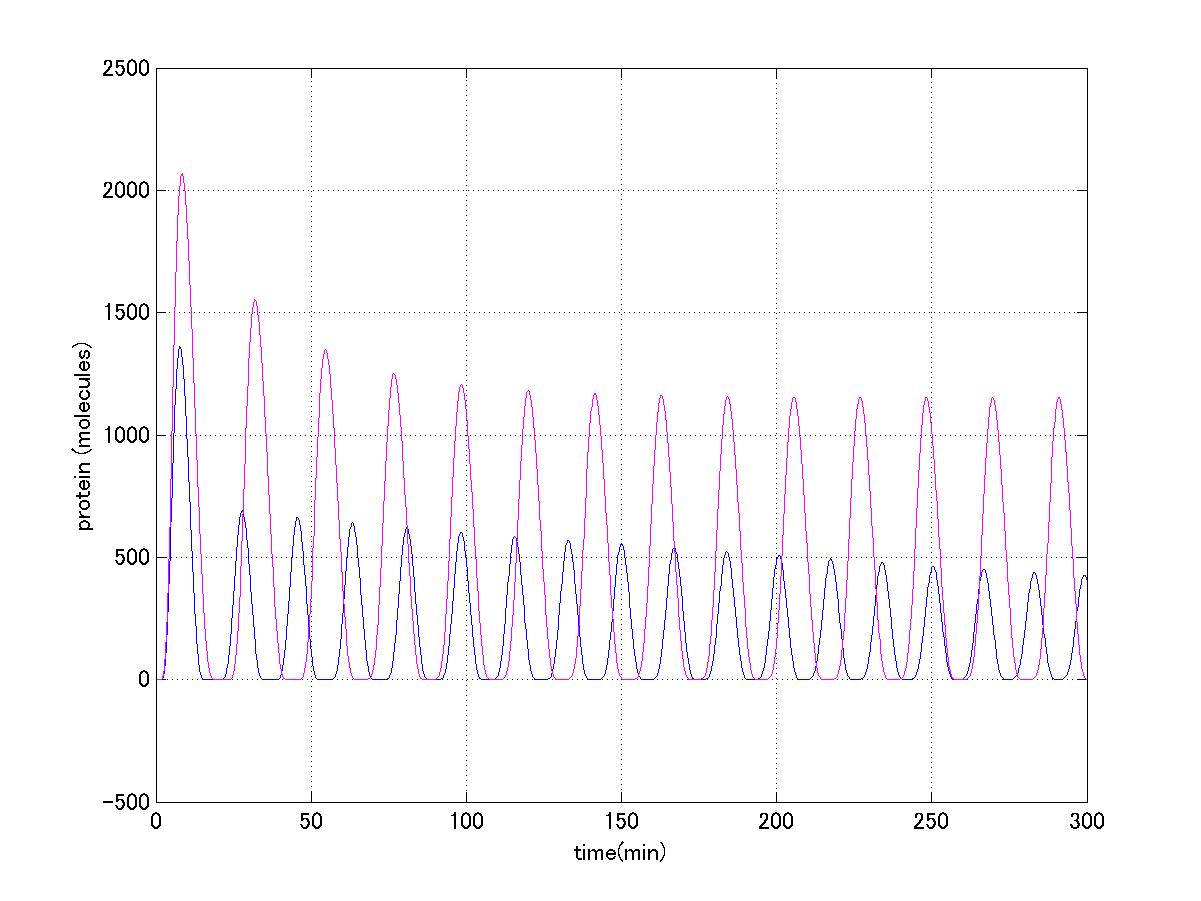

Supplement: Additional File 2 — AraC dimer oscillation time-course JPEG-format file of the two models at each arabinose and IPTG concentration. The reporter-less model is shown in blue and the reporter-containing model (Nd = 50) in red. [file 1752-0509-8-S4-S4-S2.zip › 1752-0509-8-S3-S5-S2/AraC_time_course_arabinose0.050119%_IPTG0.50119mM.jpg]

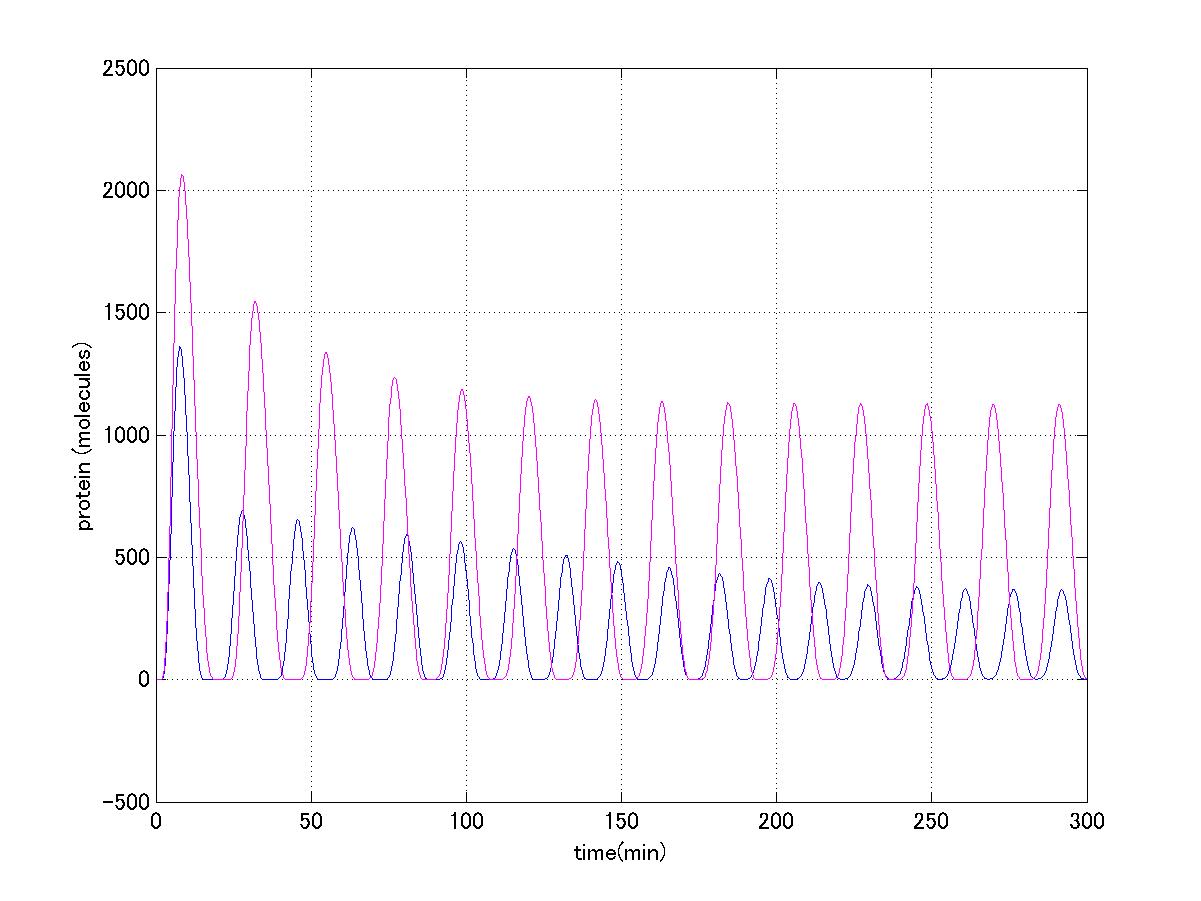

Supplement: Additional File 2 — AraC dimer oscillation time-course JPEG-format file of the two models at each arabinose and IPTG concentration. The reporter-less model is shown in blue and the reporter-containing model (Nd = 50) in red. [file 1752-0509-8-S4-S4-S2.zip › 1752-0509-8-S3-S5-S2/AraC_time_course_arabinose0.050119%_IPTG0.63096mM.jpg]

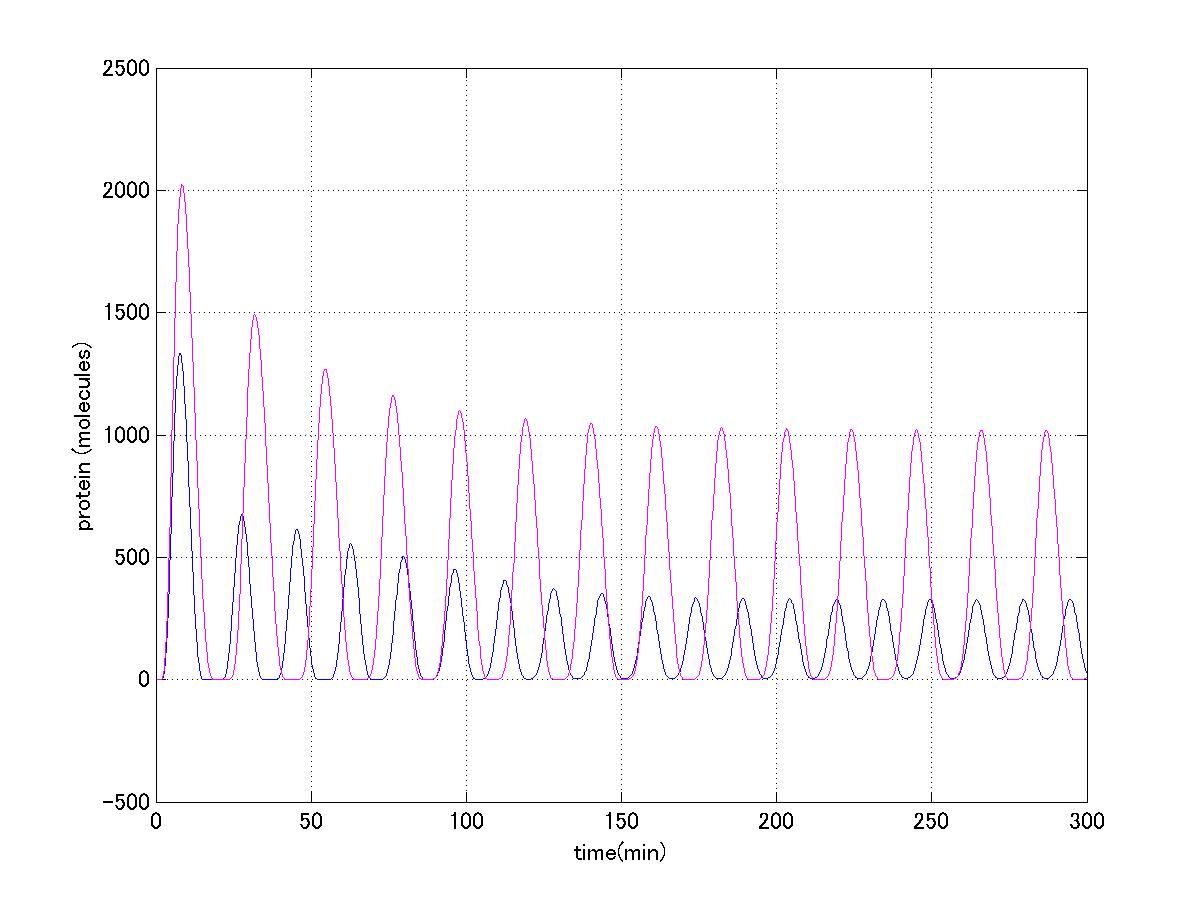

Supplement: Additional File 2 — AraC dimer oscillation time-course JPEG-format file of the two models at each arabinose and IPTG concentration. The reporter-less model is shown in blue and the reporter-containing model (Nd = 50) in red. [file 1752-0509-8-S4-S4-S2.zip › 1752-0509-8-S3-S5-S2/AraC_time_course_arabinose0.050119%_IPTG0.79433mM.jpg]

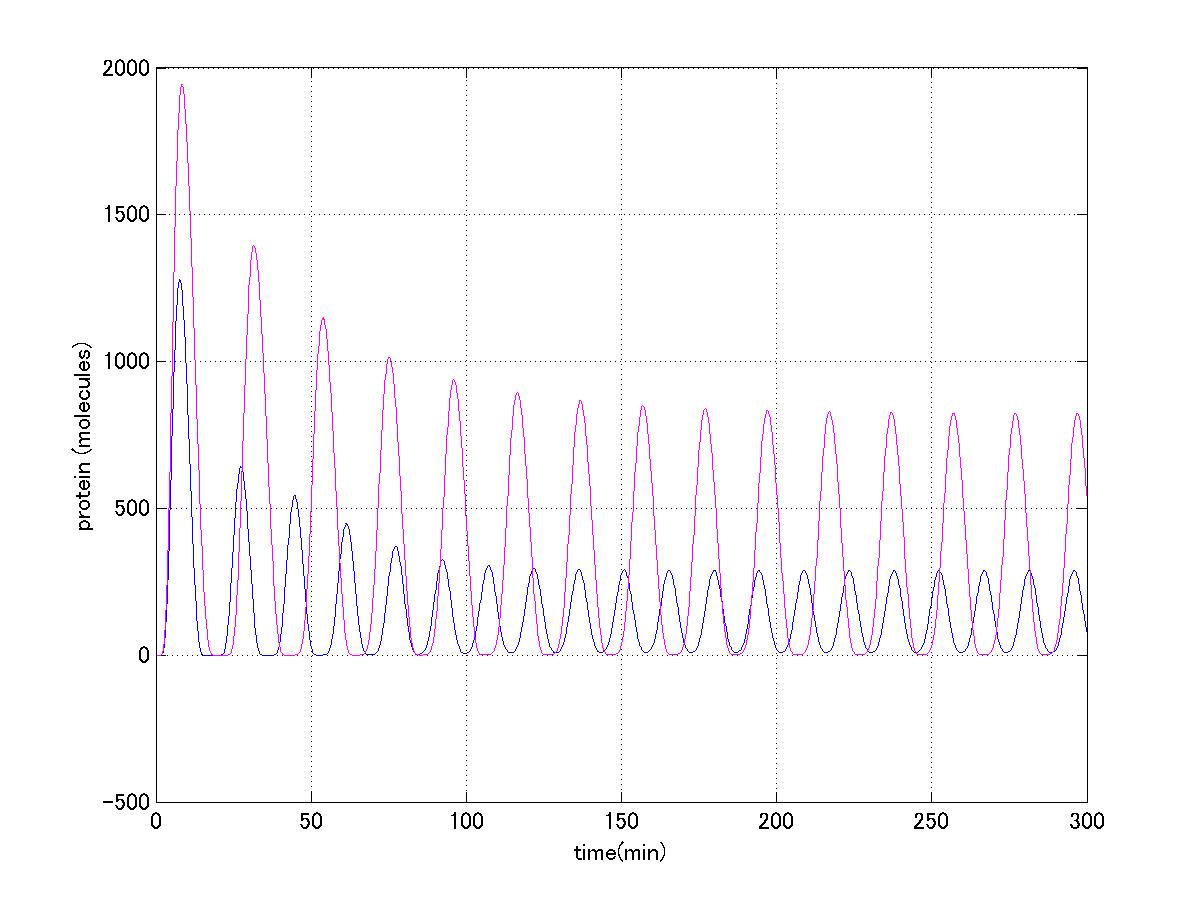

Supplement: Additional File 2 — AraC dimer oscillation time-course JPEG-format file of the two models at each arabinose and IPTG concentration. The reporter-less model is shown in blue and the reporter-containing model (Nd = 50) in red. [file 1752-0509-8-S4-S4-S2.zip › 1752-0509-8-S3-S5-S2/AraC_time_course_arabinose0.050119%_IPTG1mM.jpg]

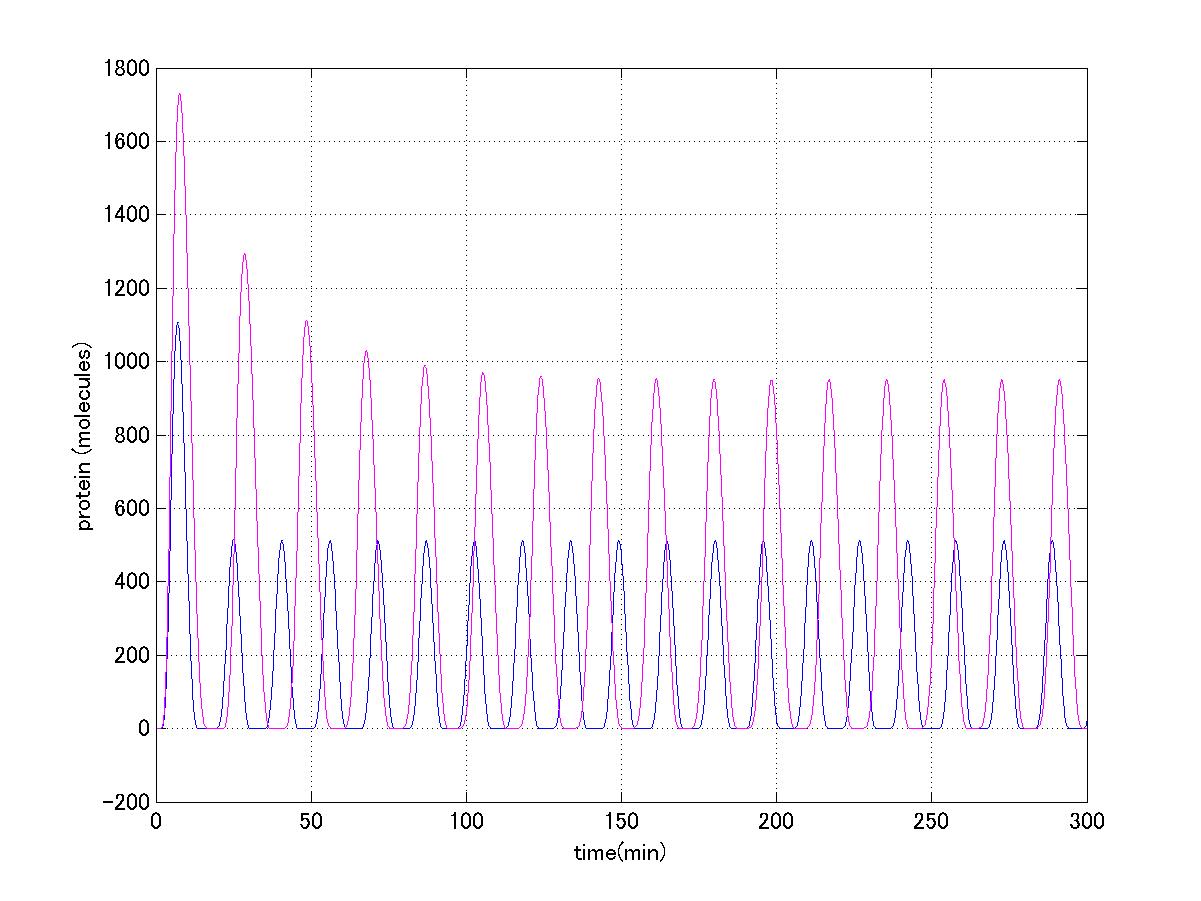

Supplement: Additional File 2 — AraC dimer oscillation time-course JPEG-format file of the two models at each arabinose and IPTG concentration. The reporter-less model is shown in blue and the reporter-containing model (Nd = 50) in red. [file 1752-0509-8-S4-S4-S2.zip › 1752-0509-8-S3-S5-S2/AraC_time_course_arabinose0.063096%_IPTG0.12589mM.jpg]

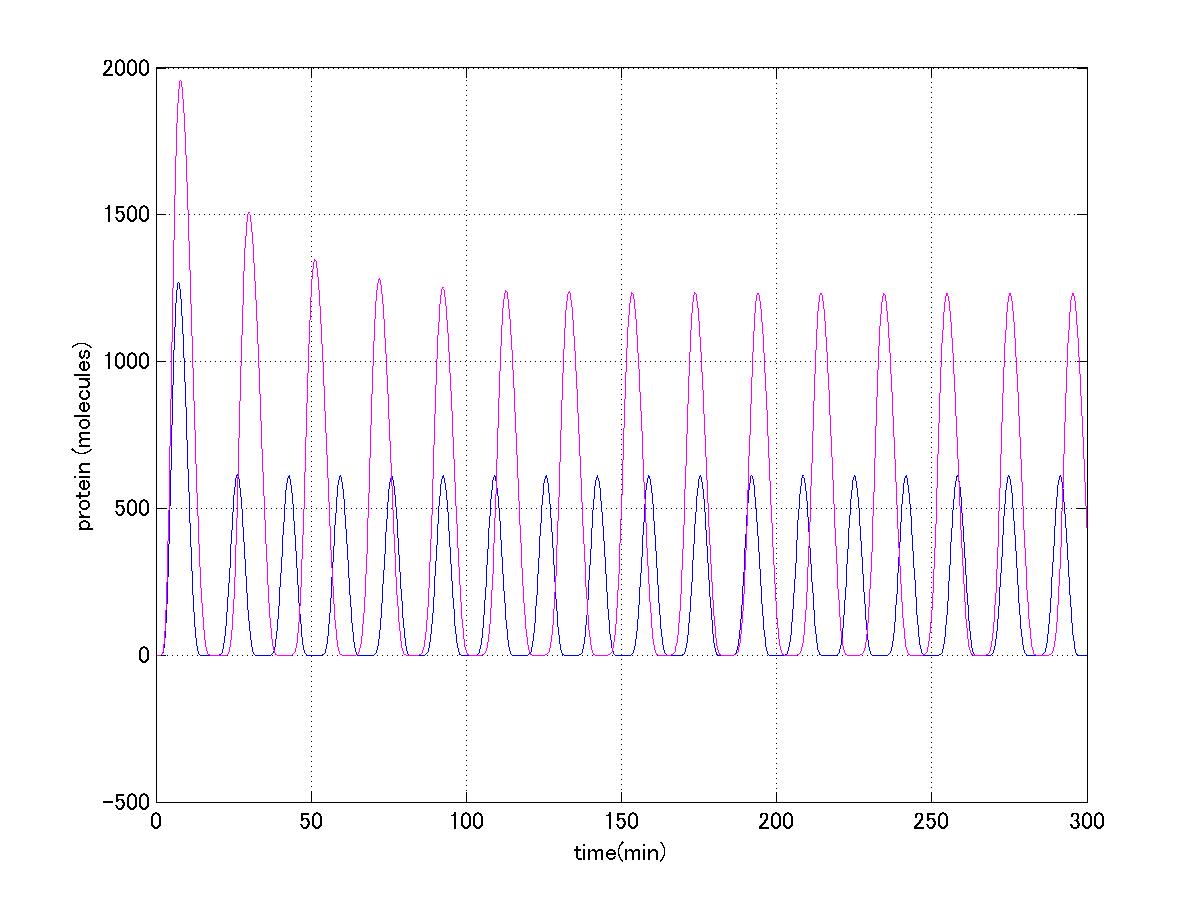

Supplement: Additional File 2 — AraC dimer oscillation time-course JPEG-format file of the two models at each arabinose and IPTG concentration. The reporter-less model is shown in blue and the reporter-containing model (Nd = 50) in red. [file 1752-0509-8-S4-S4-S2.zip › 1752-0509-8-S3-S5-S2/AraC_time_course_arabinose0.063096%_IPTG0.15849mM.jpg]

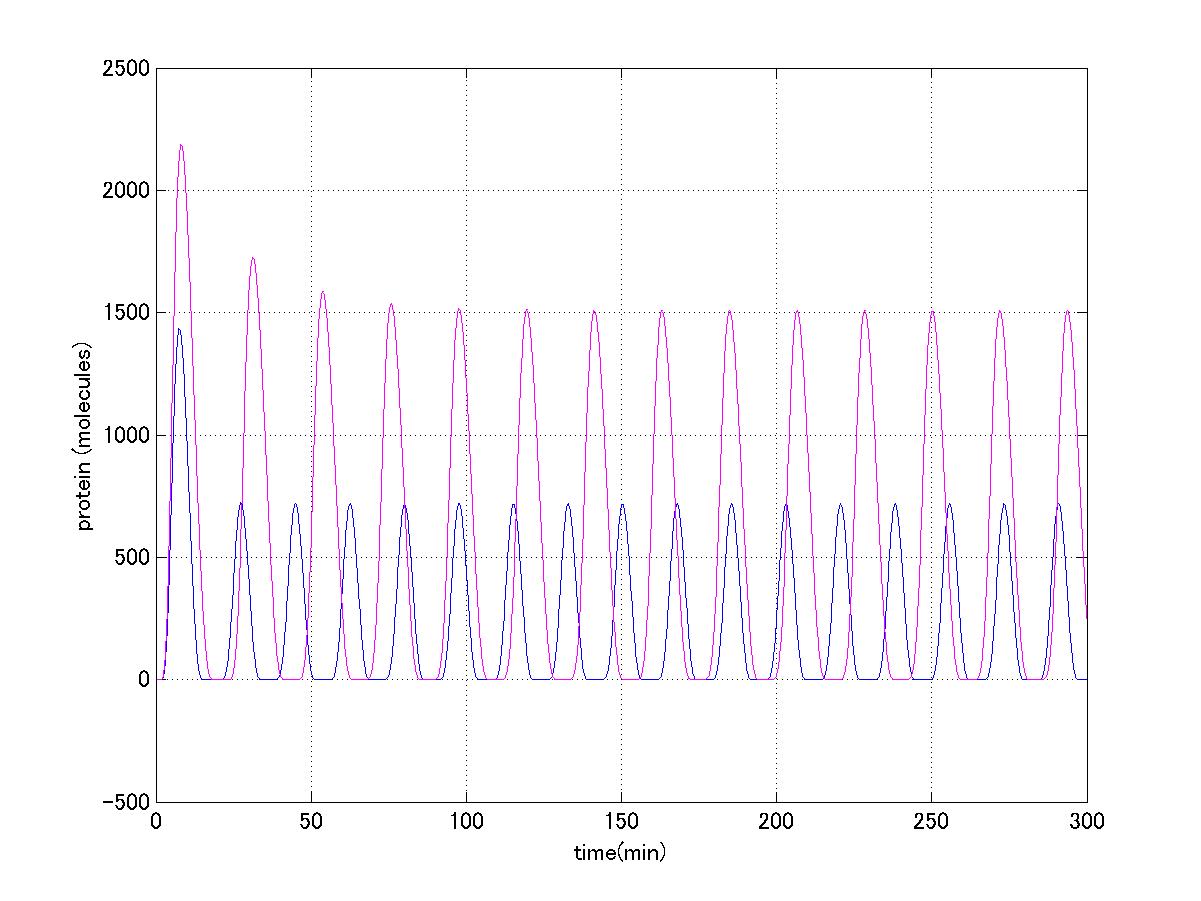

Supplement: Additional File 2 — AraC dimer oscillation time-course JPEG-format file of the two models at each arabinose and IPTG concentration. The reporter-less model is shown in blue and the reporter-containing model (Nd = 50) in red. [file 1752-0509-8-S4-S4-S2.zip › 1752-0509-8-S3-S5-S2/AraC_time_course_arabinose0.063096%_IPTG0.19953mM.jpg]

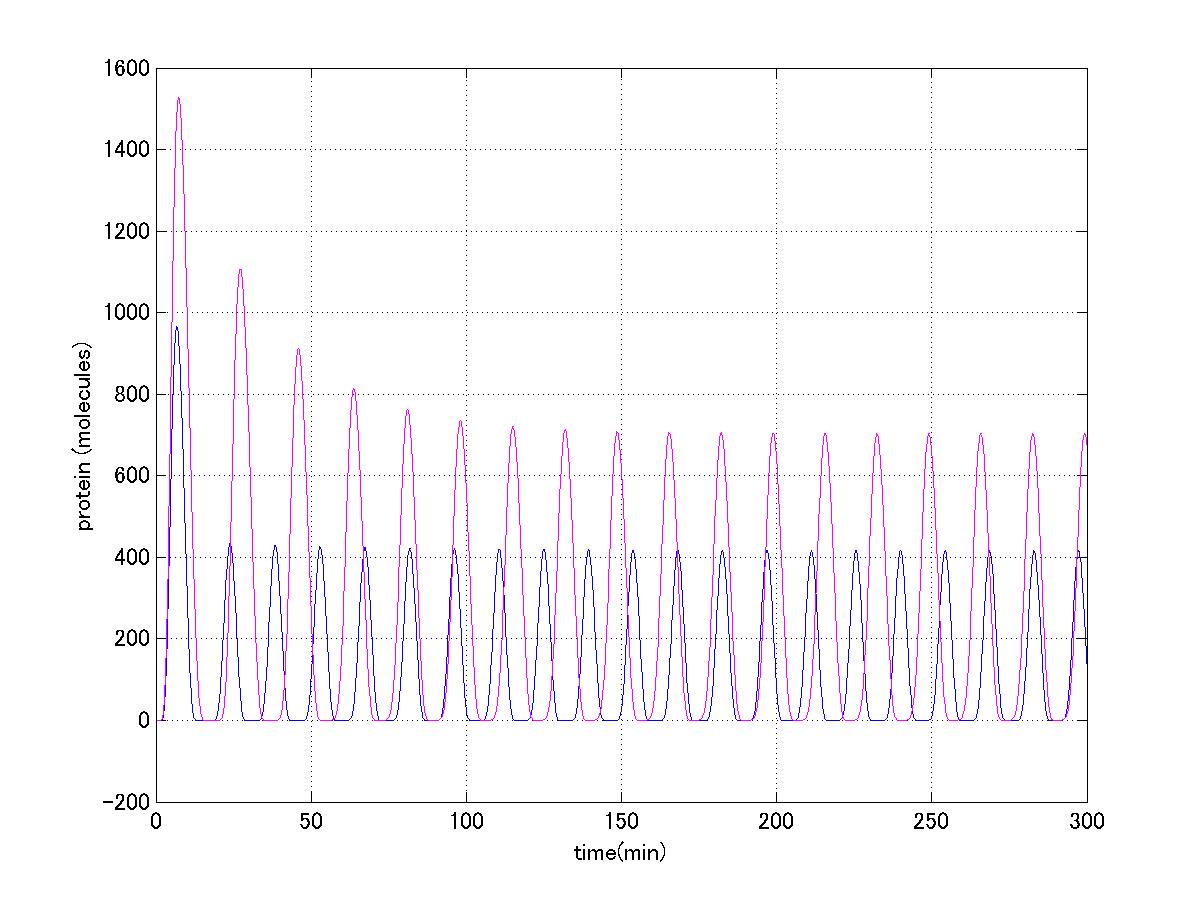

Supplement: Additional File 2 — AraC dimer oscillation time-course JPEG-format file of the two models at each arabinose and IPTG concentration. The reporter-less model is shown in blue and the reporter-containing model (Nd = 50) in red. [file 1752-0509-8-S4-S4-S2.zip › 1752-0509-8-S3-S5-S2/AraC_time_course_arabinose0.063096%_IPTG0.1mM.jpg]

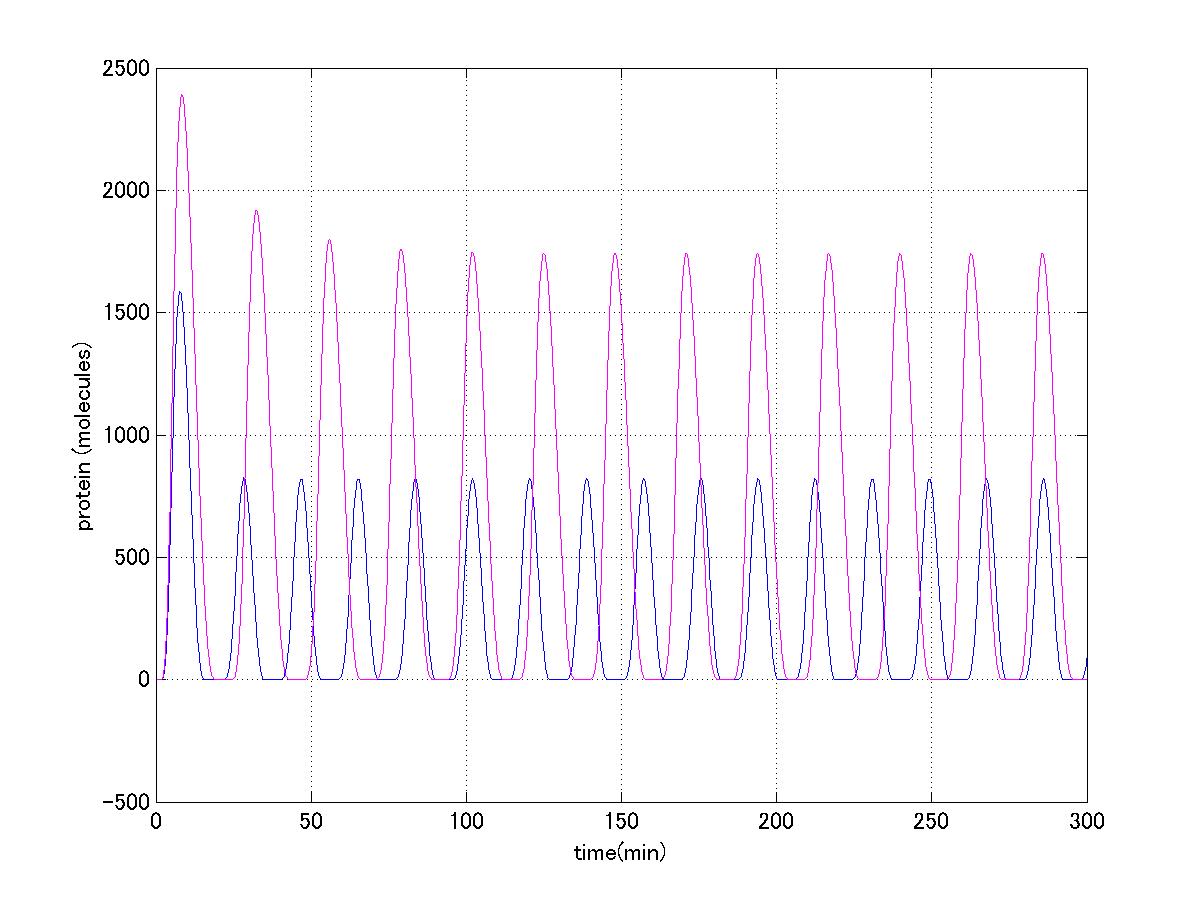

Supplement: Additional File 2 — AraC dimer oscillation time-course JPEG-format file of the two models at each arabinose and IPTG concentration. The reporter-less model is shown in blue and the reporter-containing model (Nd = 50) in red. [file 1752-0509-8-S4-S4-S2.zip › 1752-0509-8-S3-S5-S2/AraC_time_course_arabinose0.063096%_IPTG0.25119mM.jpg]

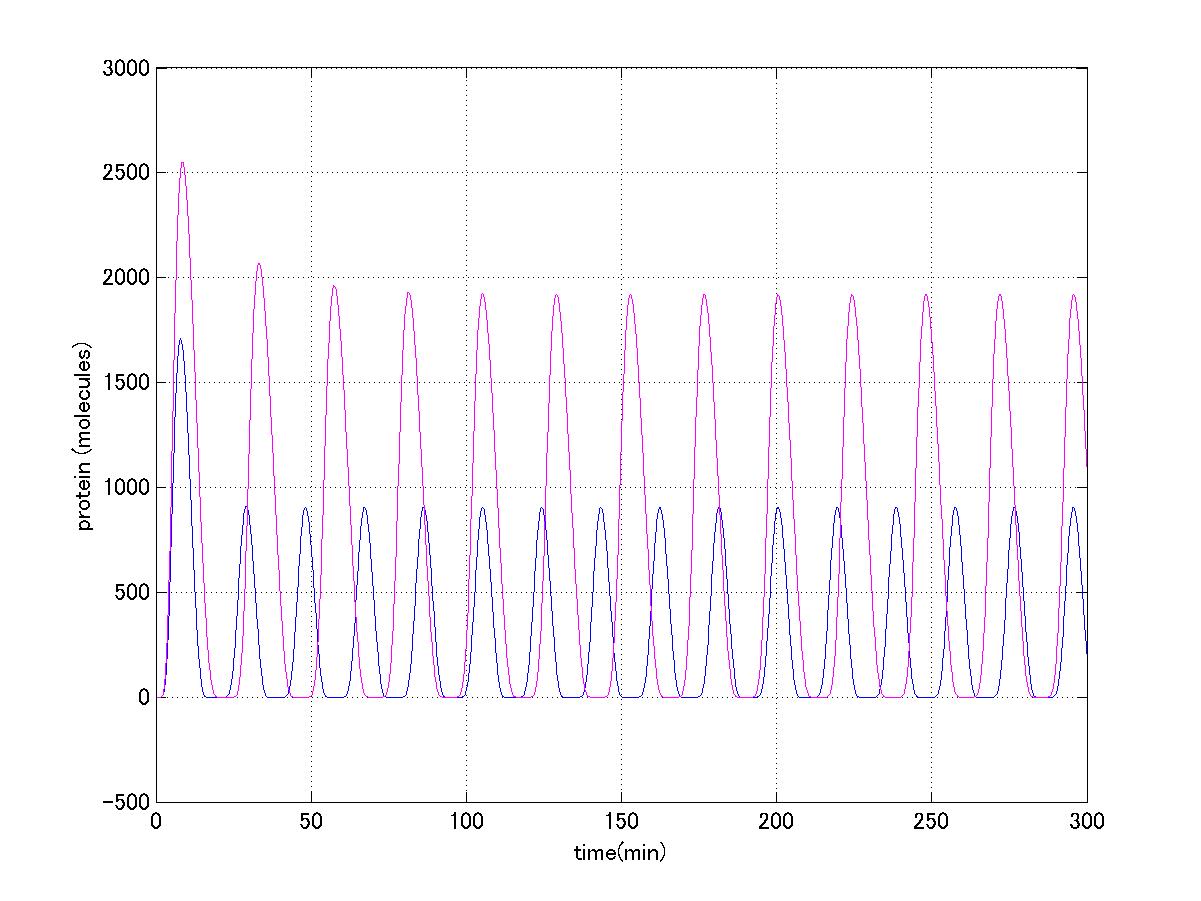

Supplement: Additional File 2 — AraC dimer oscillation time-course JPEG-format file of the two models at each arabinose and IPTG concentration. The reporter-less model is shown in blue and the reporter-containing model (Nd = 50) in red. [file 1752-0509-8-S4-S4-S2.zip › 1752-0509-8-S3-S5-S2/AraC_time_course_arabinose0.063096%_IPTG0.31623mM.jpg]

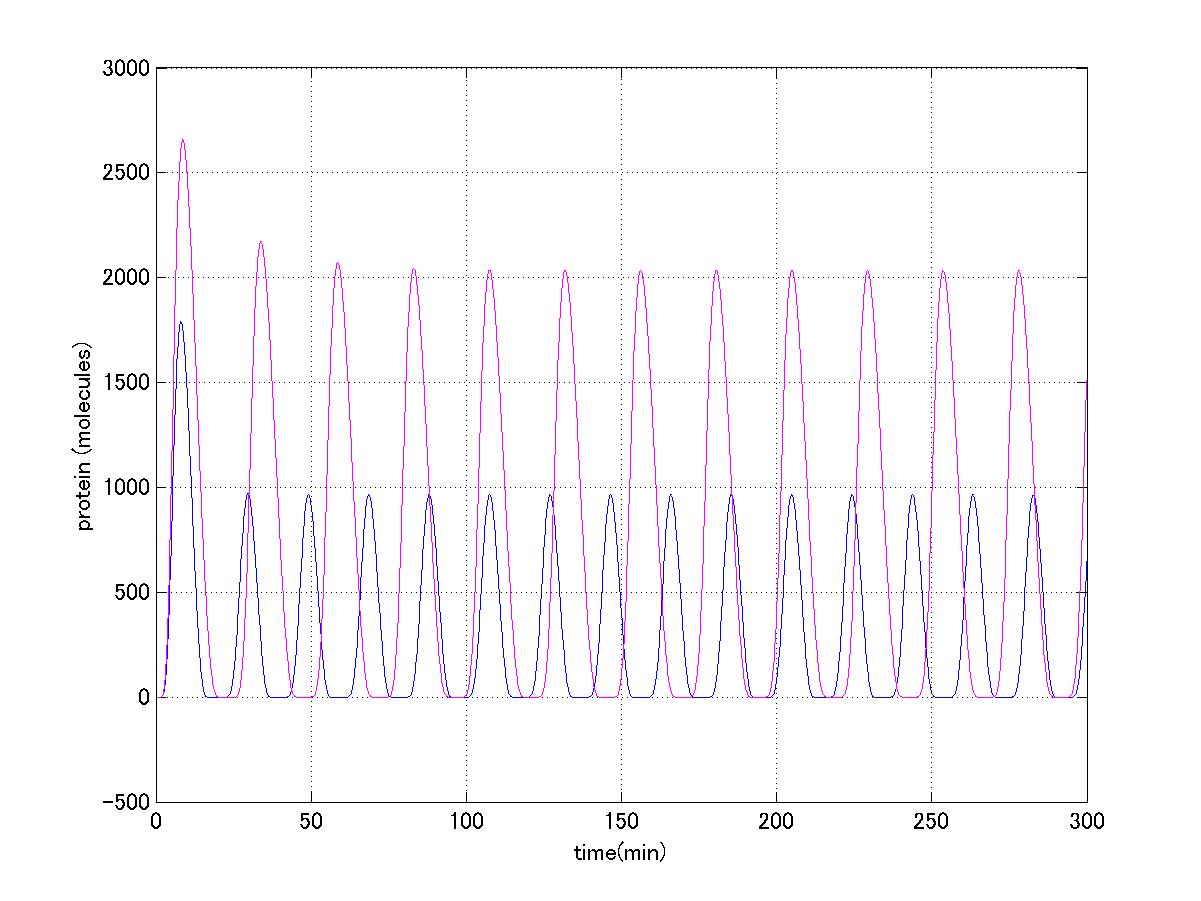

Supplement: Additional File 2 — AraC dimer oscillation time-course JPEG-format file of the two models at each arabinose and IPTG concentration. The reporter-less model is shown in blue and the reporter-containing model (Nd = 50) in red. [file 1752-0509-8-S4-S4-S2.zip › 1752-0509-8-S3-S5-S2/AraC_time_course_arabinose0.063096%_IPTG0.39811mM.jpg]

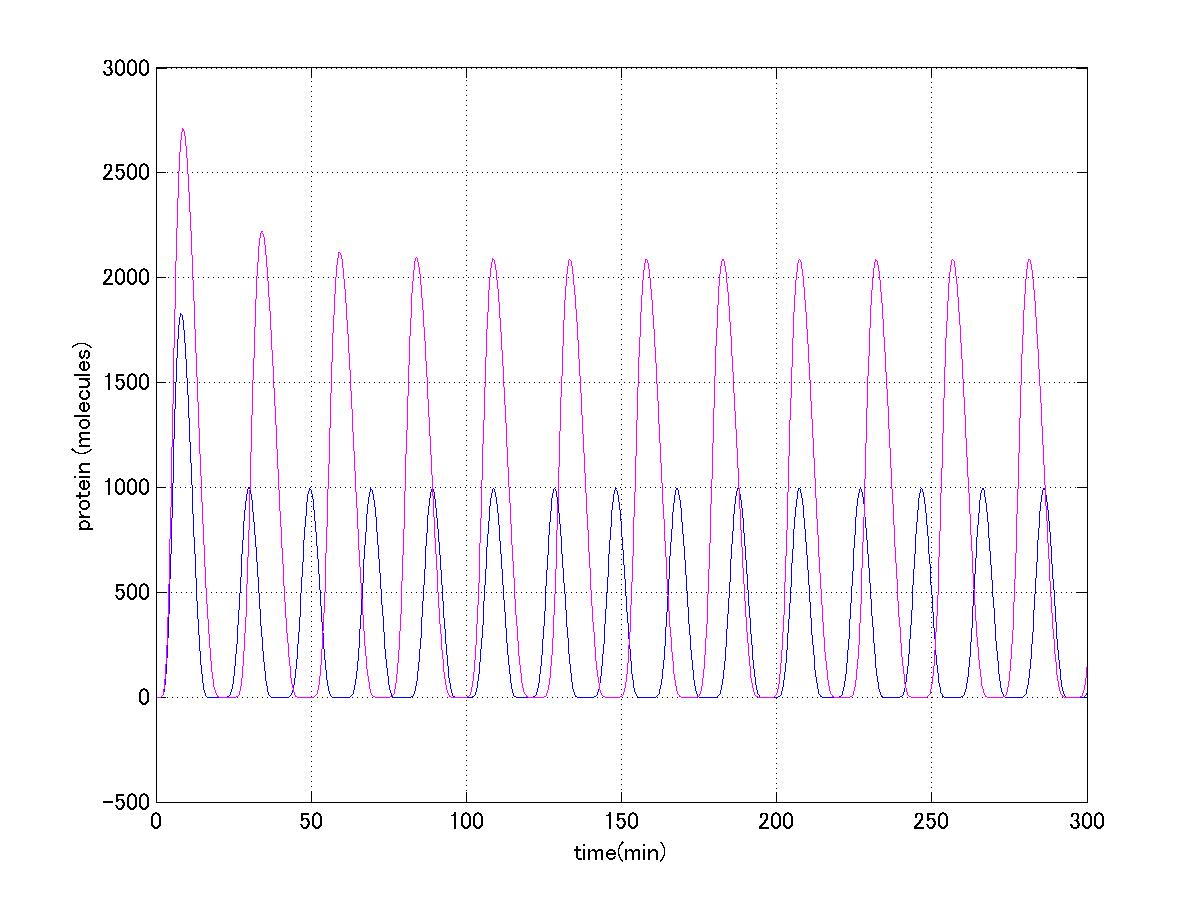

Supplement: Additional File 2 — AraC dimer oscillation time-course JPEG-format file of the two models at each arabinose and IPTG concentration. The reporter-less model is shown in blue and the reporter-containing model (Nd = 50) in red. [file 1752-0509-8-S4-S4-S2.zip › 1752-0509-8-S3-S5-S2/AraC_time_course_arabinose0.063096%_IPTG0.50119mM.jpg]

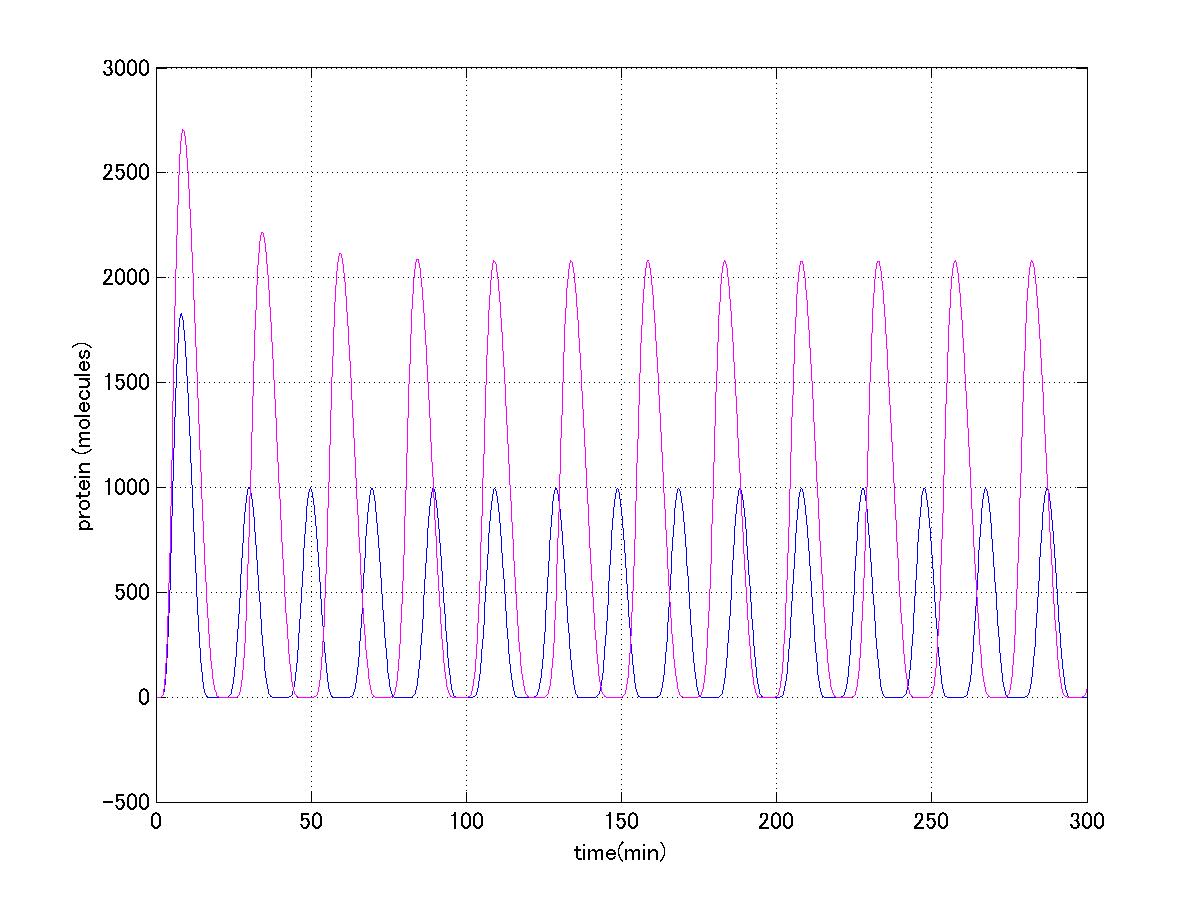

Supplement: Additional File 2 — AraC dimer oscillation time-course JPEG-format file of the two models at each arabinose and IPTG concentration. The reporter-less model is shown in blue and the reporter-containing model (Nd = 50) in red. [file 1752-0509-8-S4-S4-S2.zip › 1752-0509-8-S3-S5-S2/AraC_time_course_arabinose0.063096%_IPTG0.63096mM.jpg]

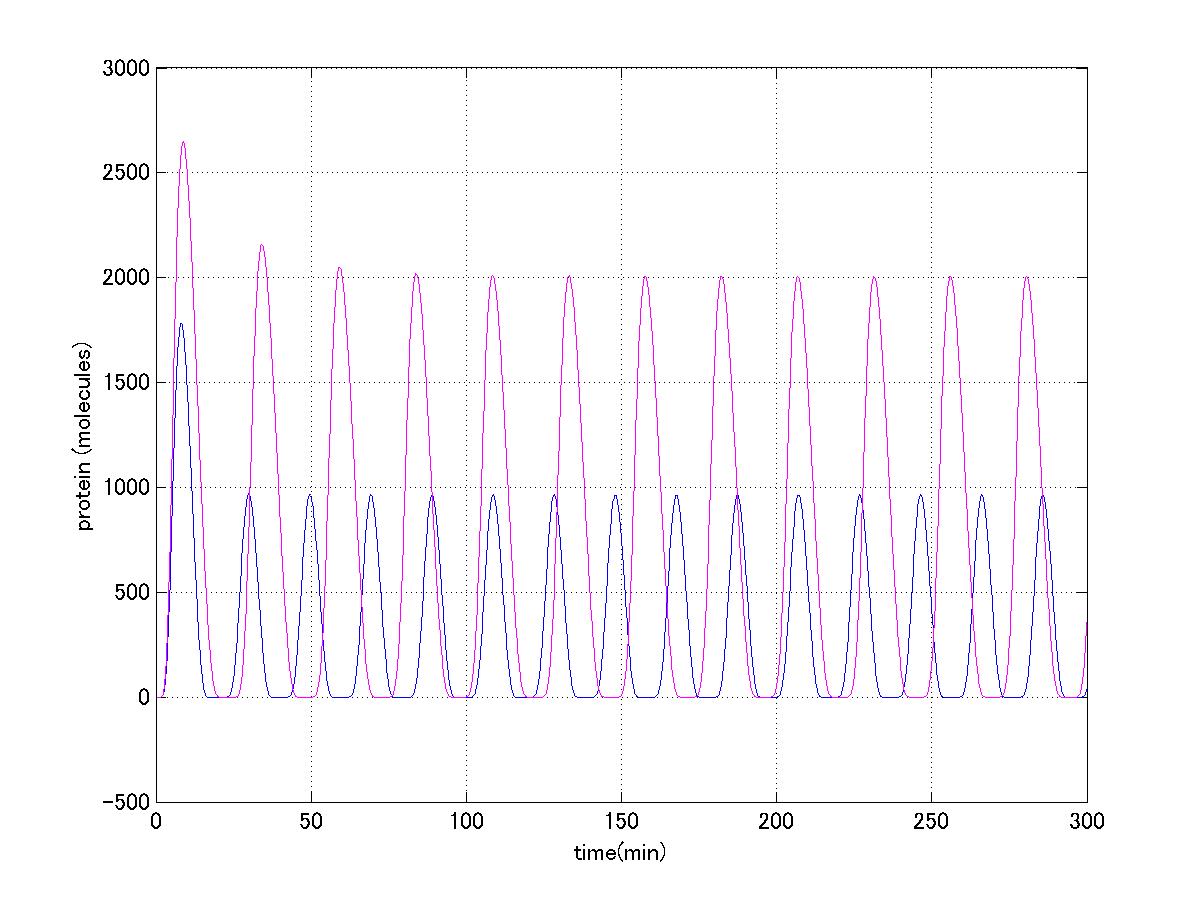

Supplement: Additional File 2 — AraC dimer oscillation time-course JPEG-format file of the two models at each arabinose and IPTG concentration. The reporter-less model is shown in blue and the reporter-containing model (Nd = 50) in red. [file 1752-0509-8-S4-S4-S2.zip › 1752-0509-8-S3-S5-S2/AraC_time_course_arabinose0.063096%_IPTG0.79433mM.jpg]

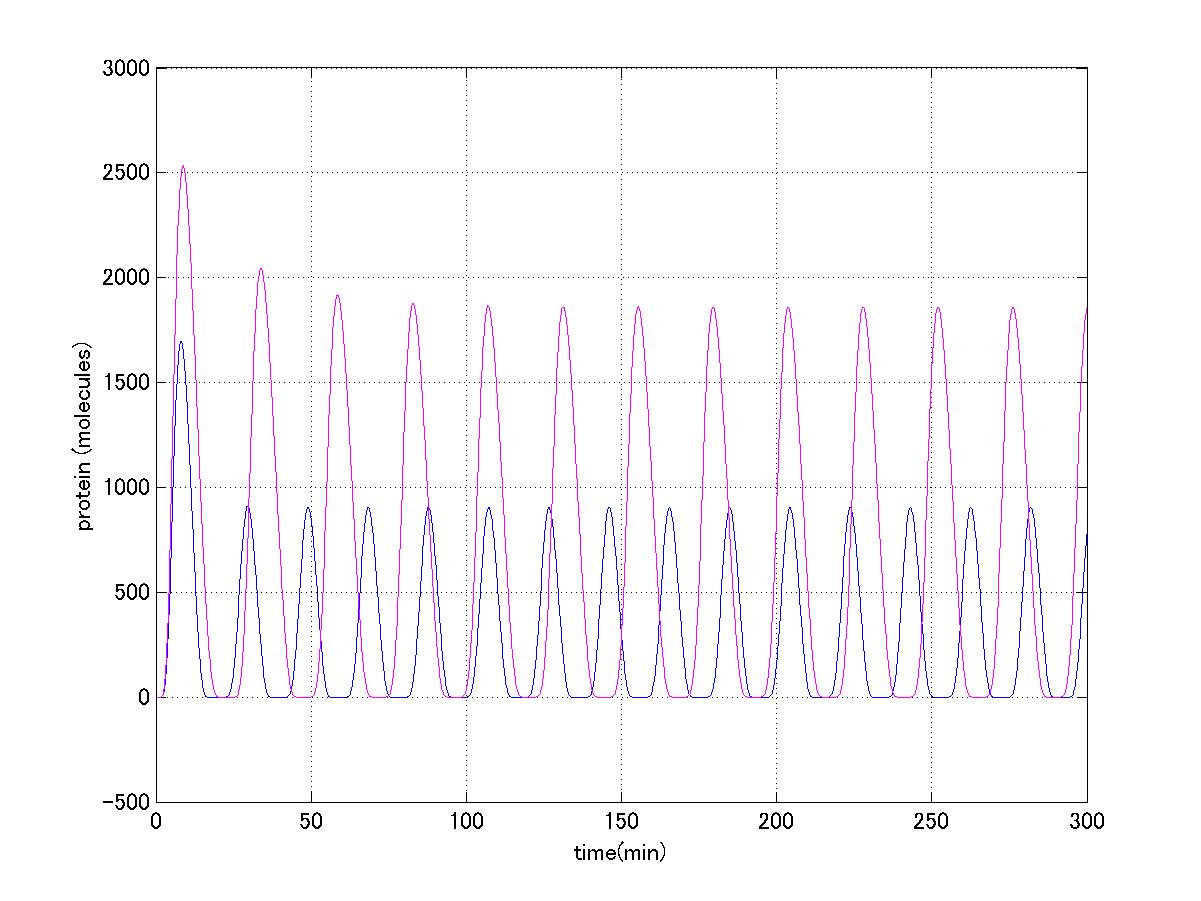

Supplement: Additional File 2 — AraC dimer oscillation time-course JPEG-format file of the two models at each arabinose and IPTG concentration. The reporter-less model is shown in blue and the reporter-containing model (Nd = 50) in red. [file 1752-0509-8-S4-S4-S2.zip › 1752-0509-8-S3-S5-S2/AraC_time_course_arabinose0.063096%_IPTG1mM.jpg]

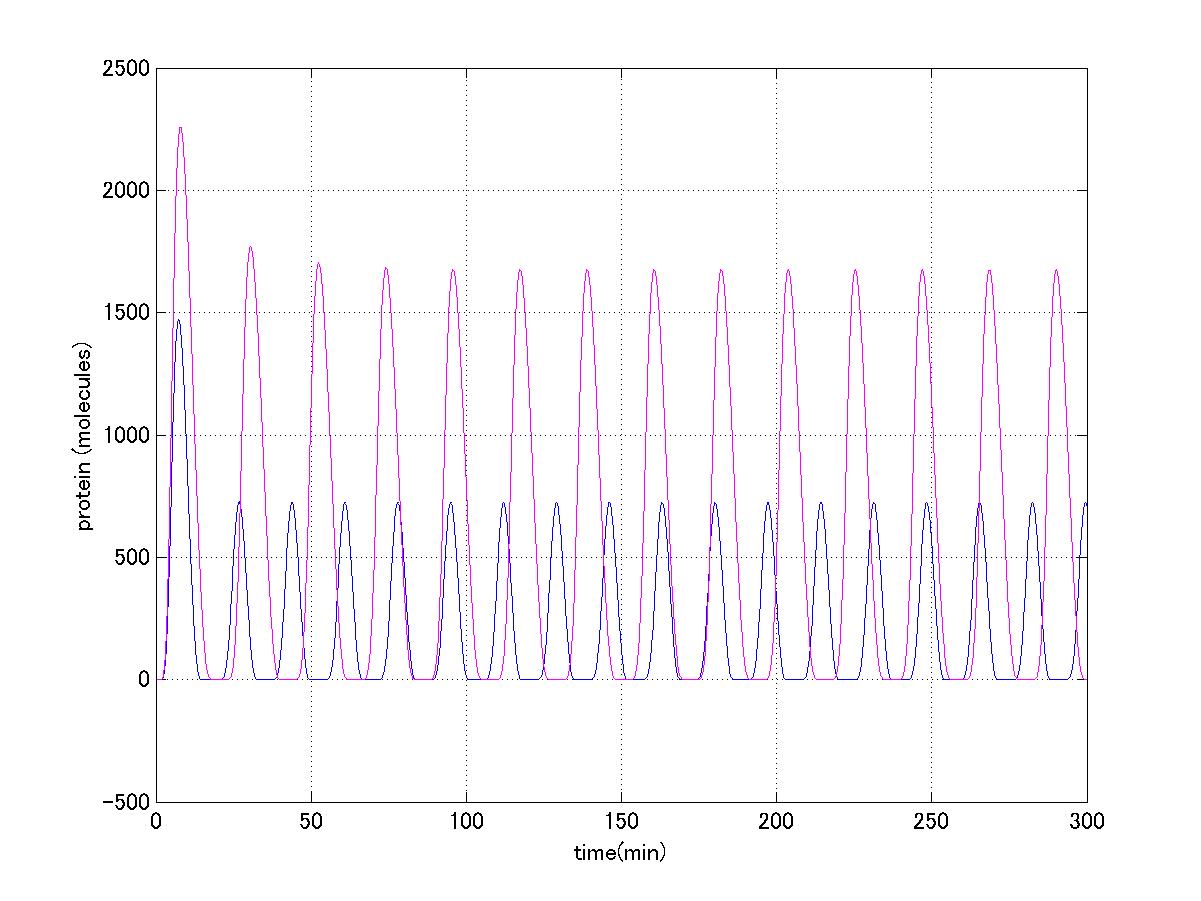

Supplement: Additional File 2 — AraC dimer oscillation time-course JPEG-format file of the two models at each arabinose and IPTG concentration. The reporter-less model is shown in blue and the reporter-containing model (Nd = 50) in red. [file 1752-0509-8-S4-S4-S2.zip › 1752-0509-8-S3-S5-S2/AraC_time_course_arabinose0.079433%_IPTG0.12589mM.jpg]

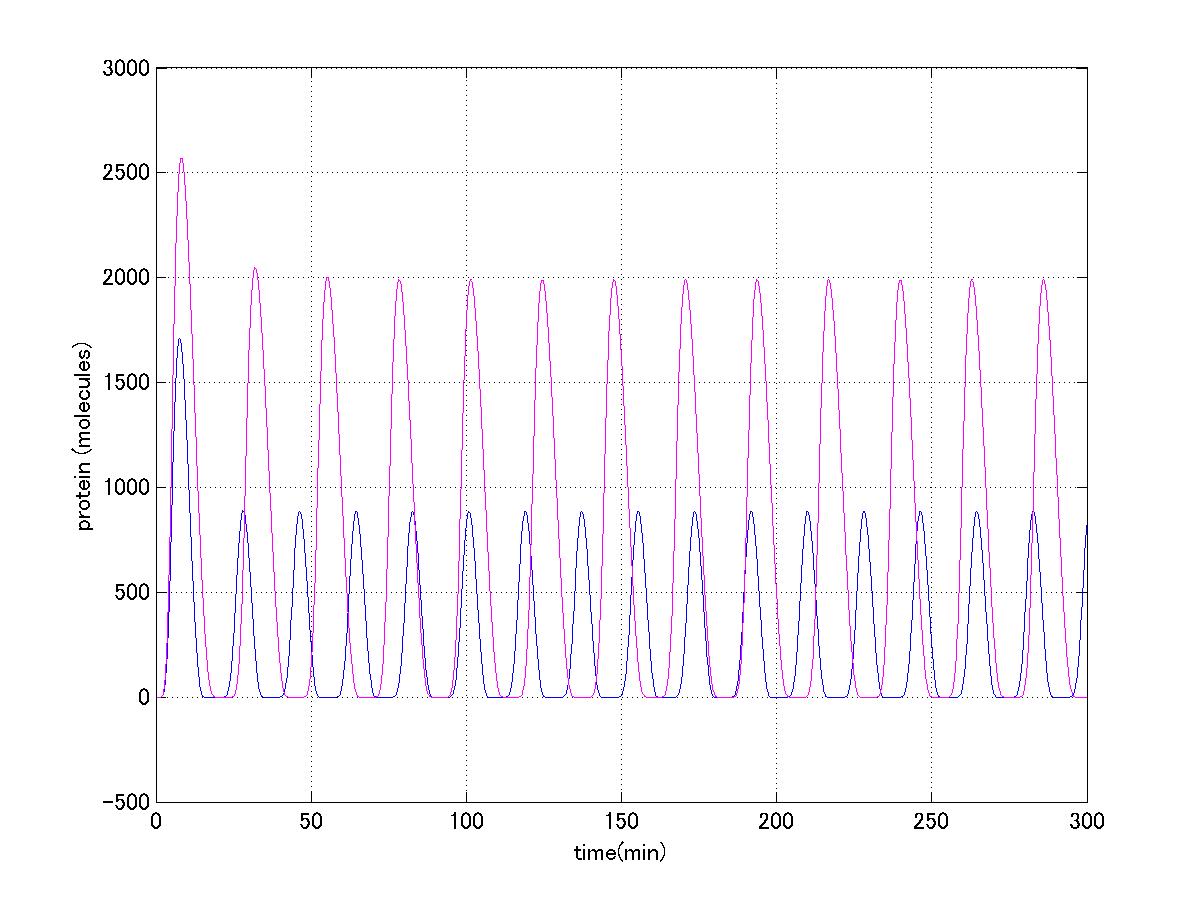

Supplement: Additional File 2 — AraC dimer oscillation time-course JPEG-format file of the two models at each arabinose and IPTG concentration. The reporter-less model is shown in blue and the reporter-containing model (Nd = 50) in red. [file 1752-0509-8-S4-S4-S2.zip › 1752-0509-8-S3-S5-S2/AraC_time_course_arabinose0.079433%_IPTG0.15849mM.jpg]

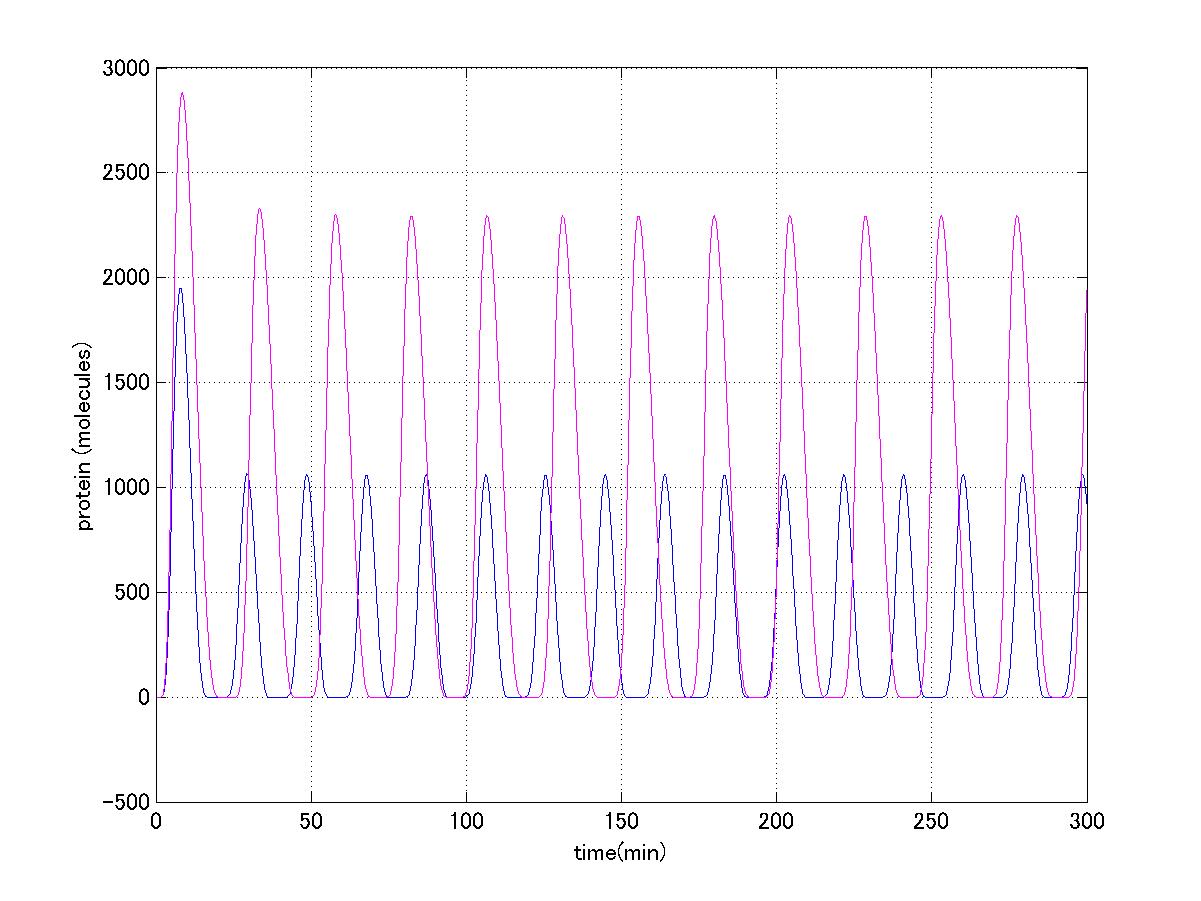

Supplement: Additional File 2 — AraC dimer oscillation time-course JPEG-format file of the two models at each arabinose and IPTG concentration. The reporter-less model is shown in blue and the reporter-containing model (Nd = 50) in red. [file 1752-0509-8-S4-S4-S2.zip › 1752-0509-8-S3-S5-S2/AraC_time_course_arabinose0.079433%_IPTG0.19953mM.jpg]

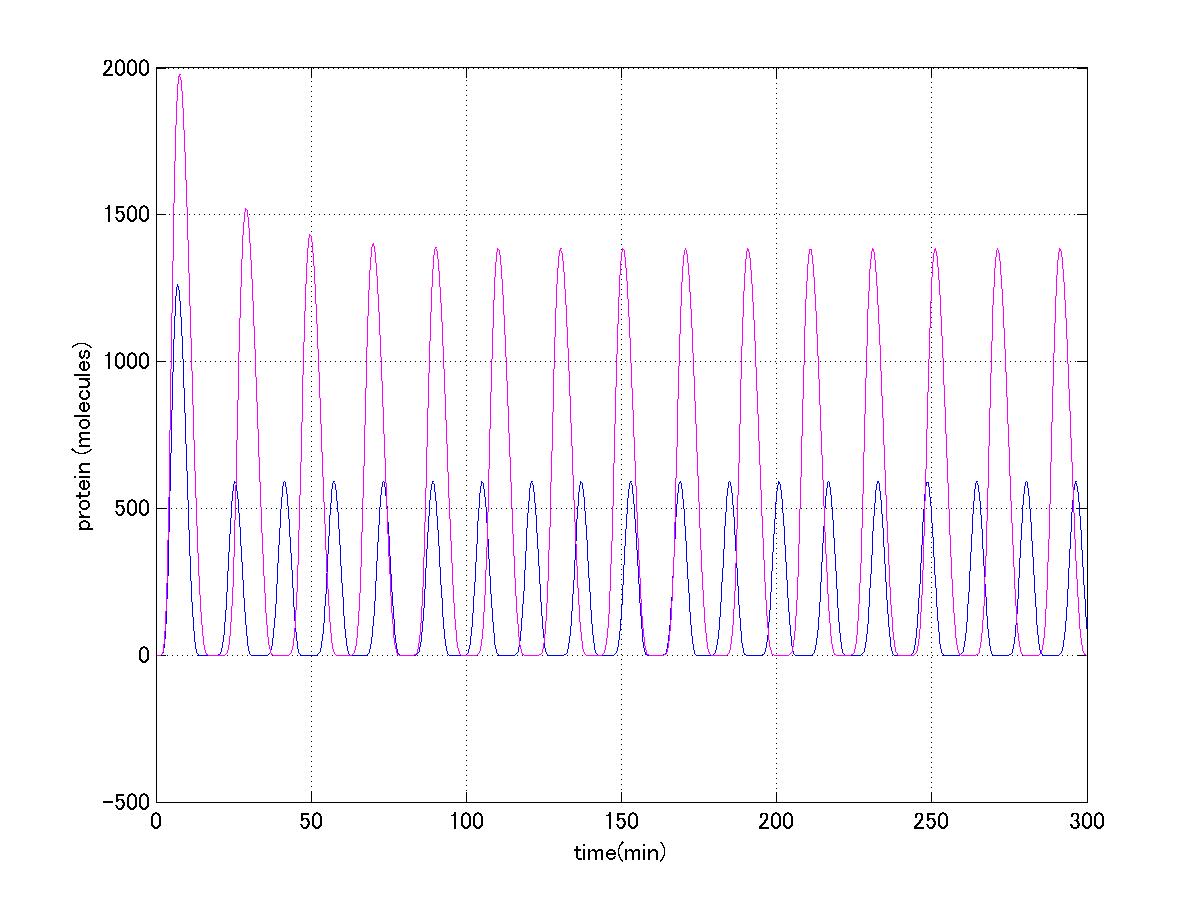

Supplement: Additional File 2 — AraC dimer oscillation time-course JPEG-format file of the two models at each arabinose and IPTG concentration. The reporter-less model is shown in blue and the reporter-containing model (Nd = 50) in red. [file 1752-0509-8-S4-S4-S2.zip › 1752-0509-8-S3-S5-S2/AraC_time_course_arabinose0.079433%_IPTG0.1mM.jpg]

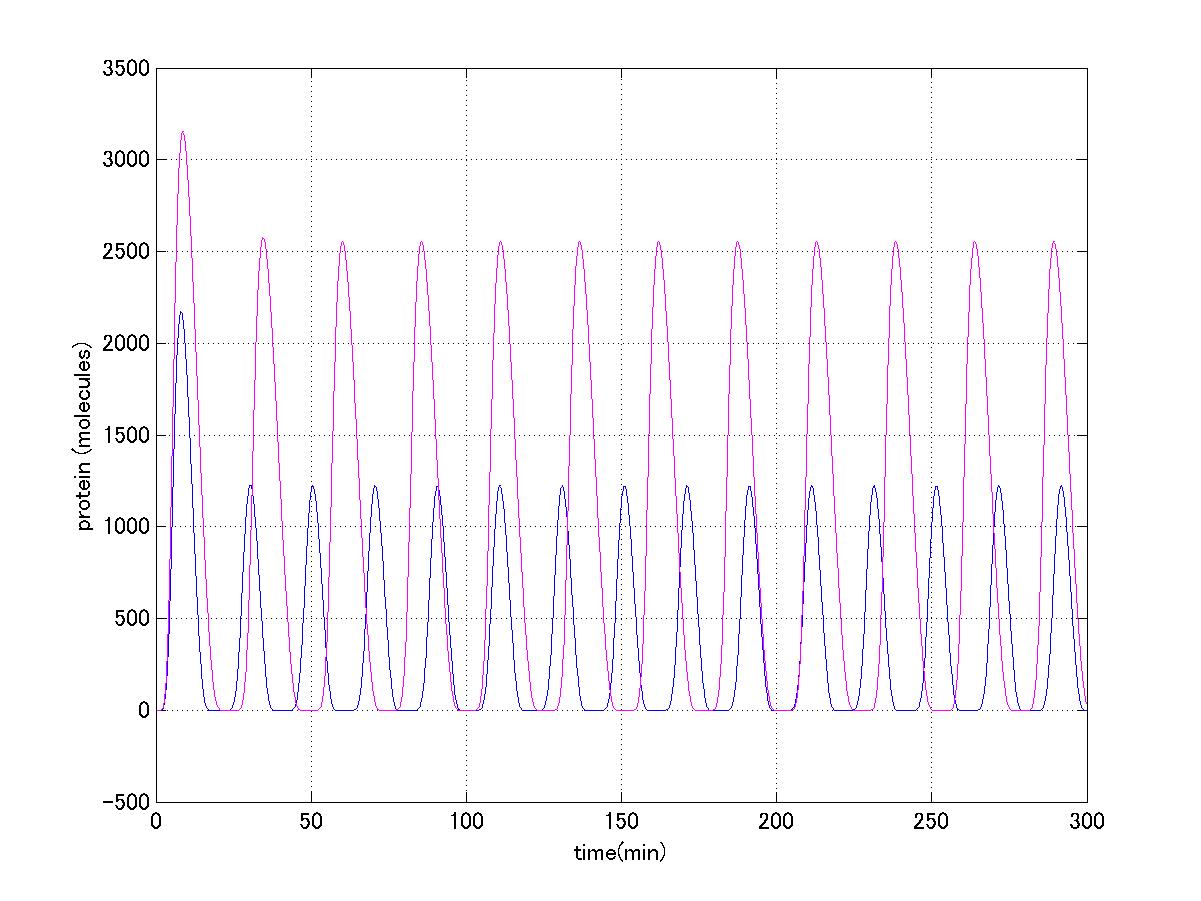

Supplement: Additional File 2 — AraC dimer oscillation time-course JPEG-format file of the two models at each arabinose and IPTG concentration. The reporter-less model is shown in blue and the reporter-containing model (Nd = 50) in red. [file 1752-0509-8-S4-S4-S2.zip › 1752-0509-8-S3-S5-S2/AraC_time_course_arabinose0.079433%_IPTG0.25119mM.jpg]

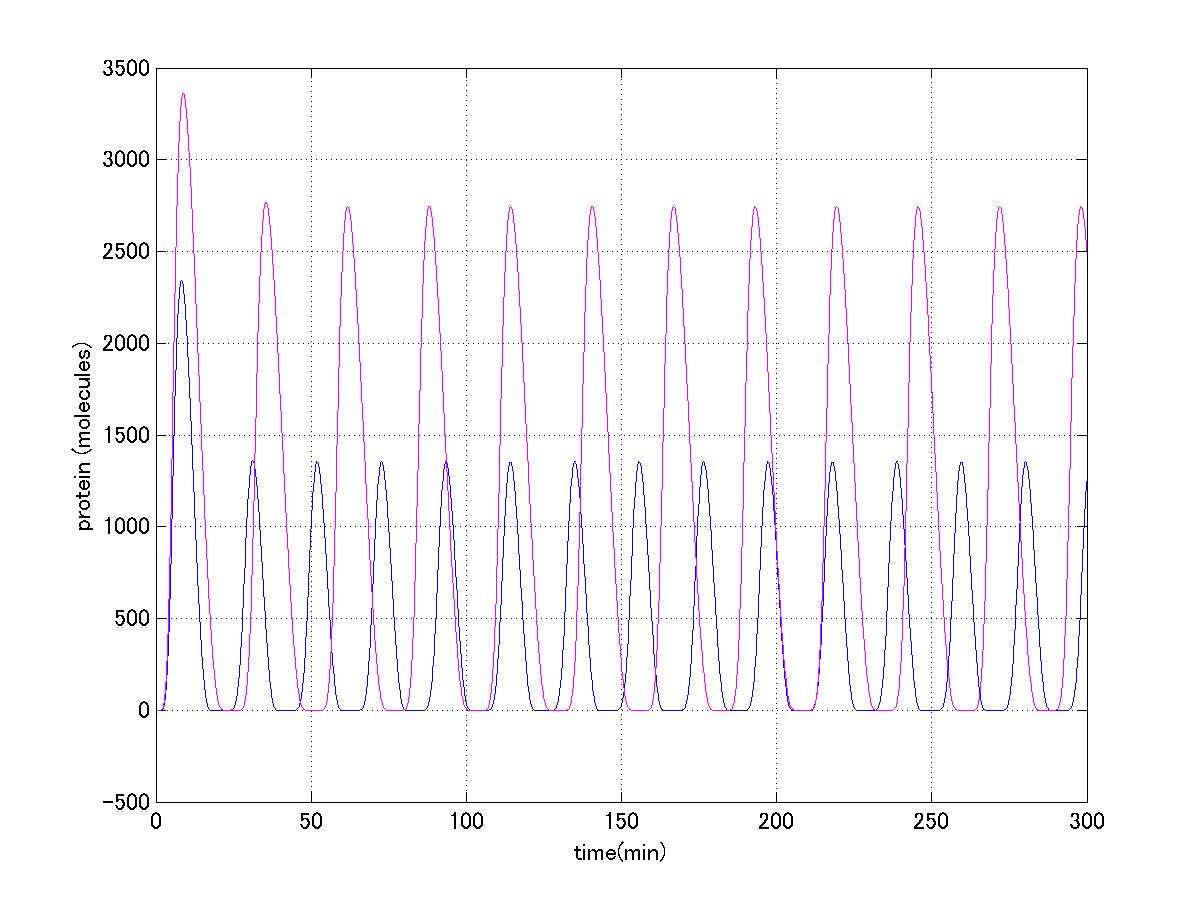

Supplement: Additional File 2 — AraC dimer oscillation time-course JPEG-format file of the two models at each arabinose and IPTG concentration. The reporter-less model is shown in blue and the reporter-containing model (Nd = 50) in red. [file 1752-0509-8-S4-S4-S2.zip › 1752-0509-8-S3-S5-S2/AraC_time_course_arabinose0.079433%_IPTG0.31623mM.jpg]

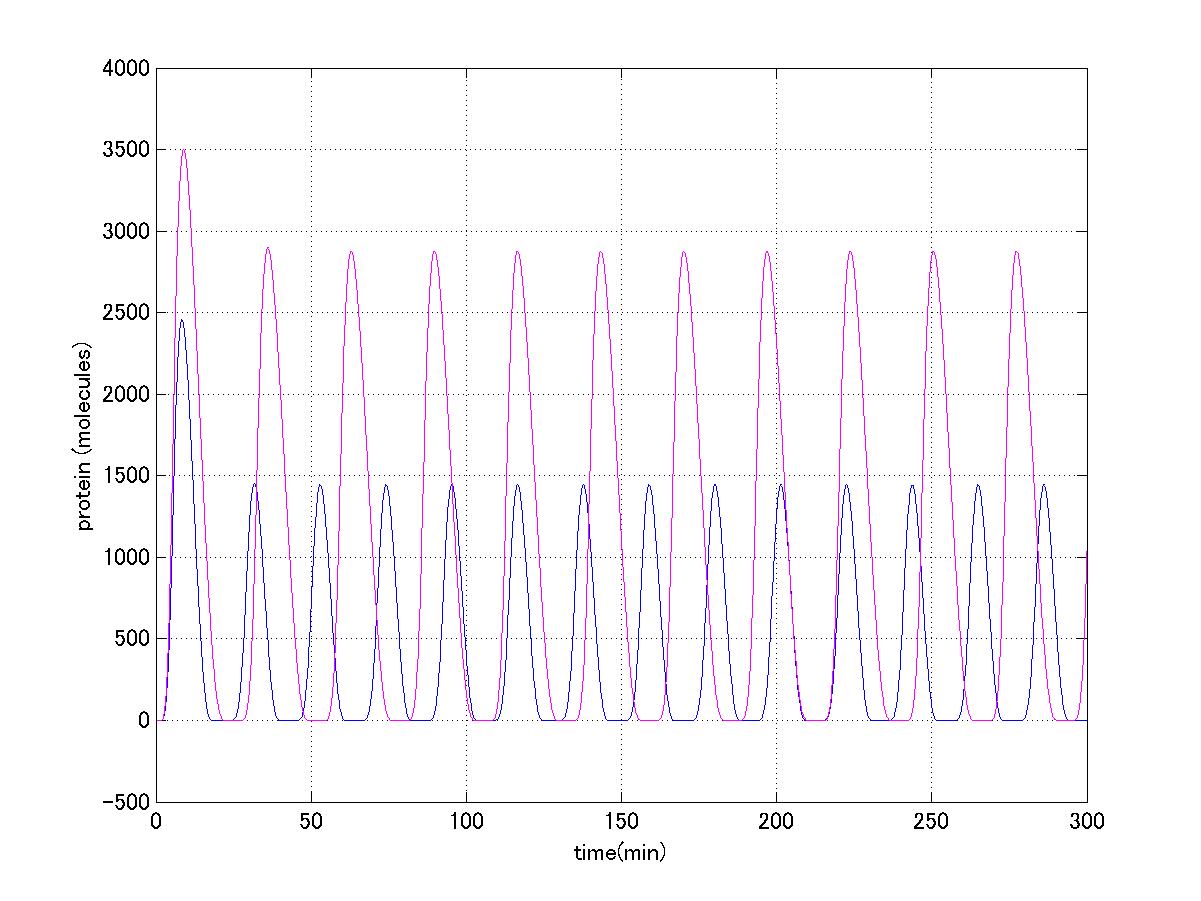

Supplement: Additional File 2 — AraC dimer oscillation time-course JPEG-format file of the two models at each arabinose and IPTG concentration. The reporter-less model is shown in blue and the reporter-containing model (Nd = 50) in red. [file 1752-0509-8-S4-S4-S2.zip › 1752-0509-8-S3-S5-S2/AraC_time_course_arabinose0.079433%_IPTG0.39811mM.jpg]

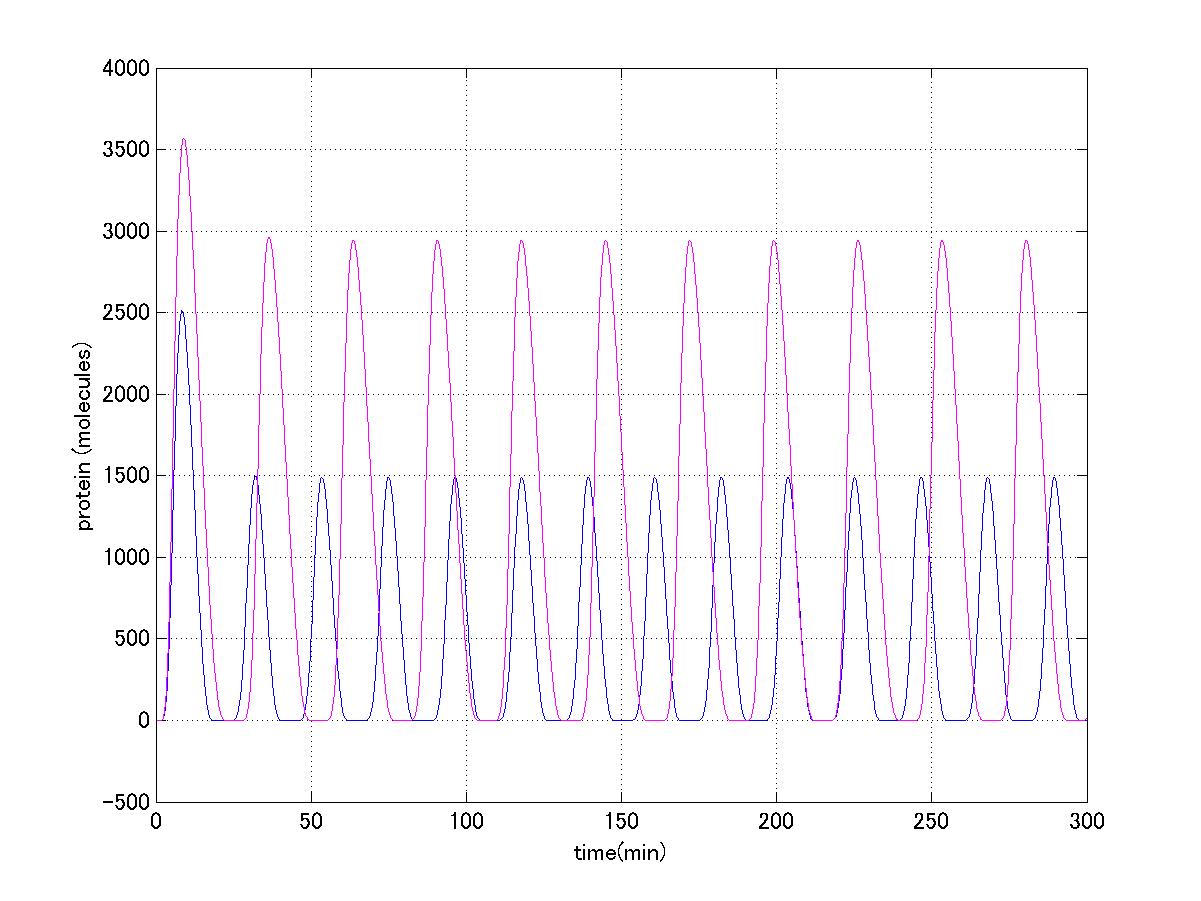

Supplement: Additional File 2 — AraC dimer oscillation time-course JPEG-format file of the two models at each arabinose and IPTG concentration. The reporter-less model is shown in blue and the reporter-containing model (Nd = 50) in red. [file 1752-0509-8-S4-S4-S2.zip › 1752-0509-8-S3-S5-S2/AraC_time_course_arabinose0.079433%_IPTG0.50119mM.jpg]

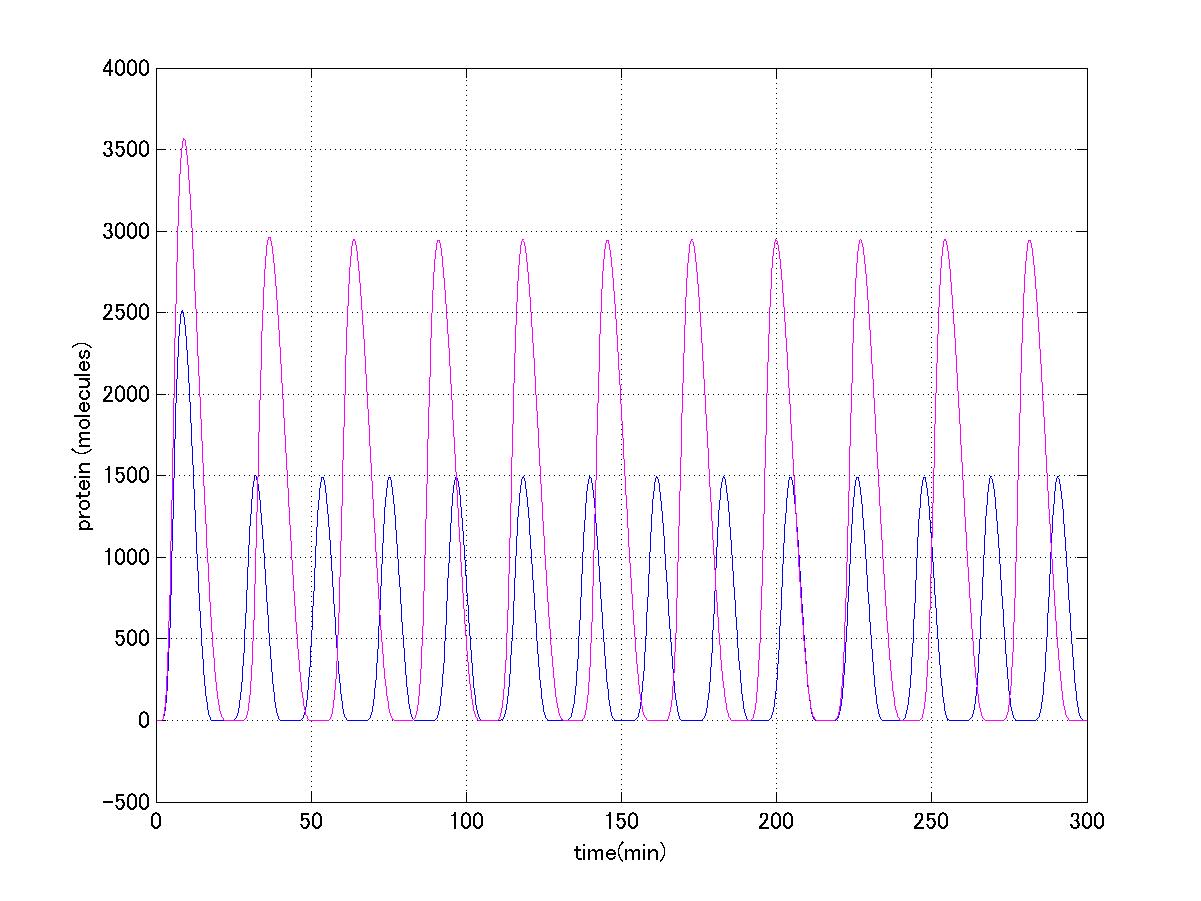

Supplement: Additional File 2 — AraC dimer oscillation time-course JPEG-format file of the two models at each arabinose and IPTG concentration. The reporter-less model is shown in blue and the reporter-containing model (Nd = 50) in red. [file 1752-0509-8-S4-S4-S2.zip › 1752-0509-8-S3-S5-S2/AraC_time_course_arabinose0.079433%_IPTG0.63096mM.jpg]

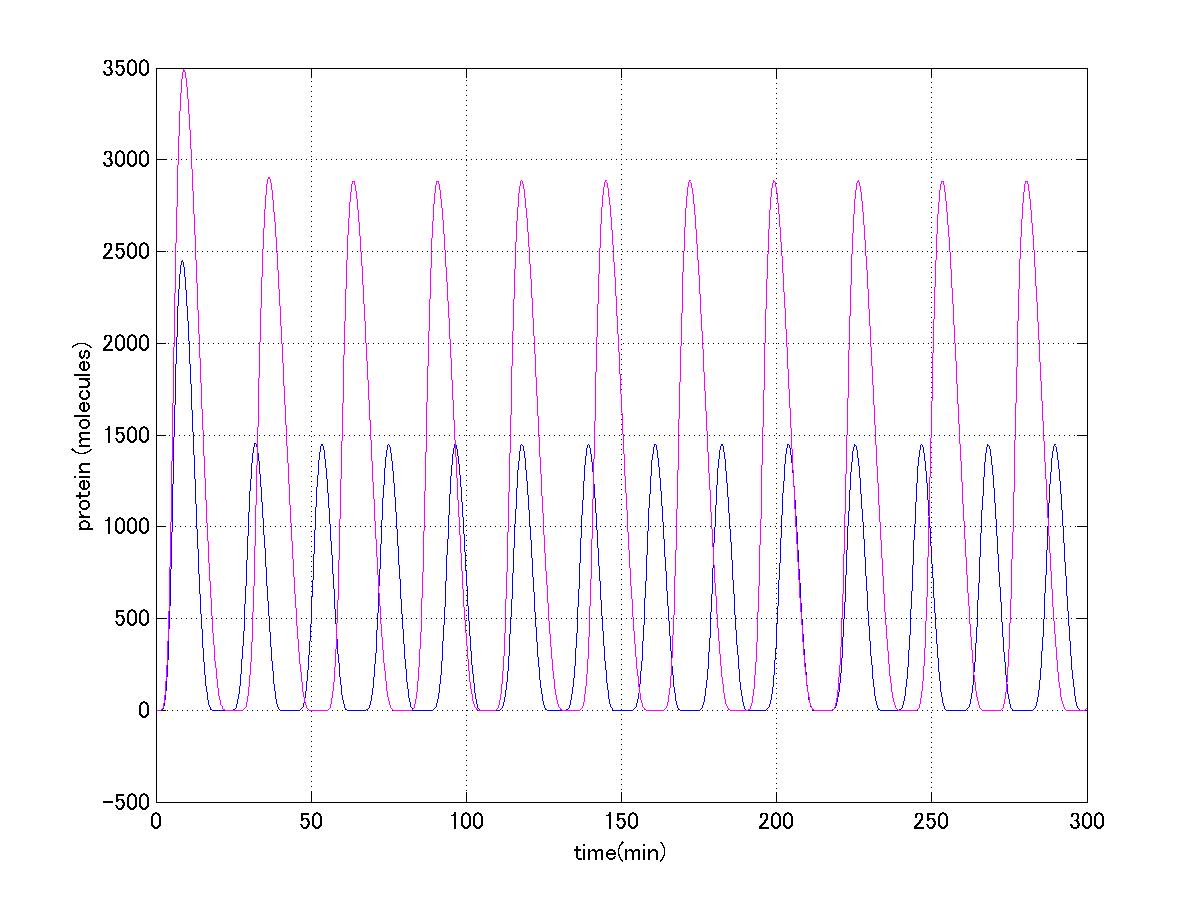

Supplement: Additional File 2 — AraC dimer oscillation time-course JPEG-format file of the two models at each arabinose and IPTG concentration. The reporter-less model is shown in blue and the reporter-containing model (Nd = 50) in red. [file 1752-0509-8-S4-S4-S2.zip › 1752-0509-8-S3-S5-S2/AraC_time_course_arabinose0.079433%_IPTG0.79433mM.jpg]

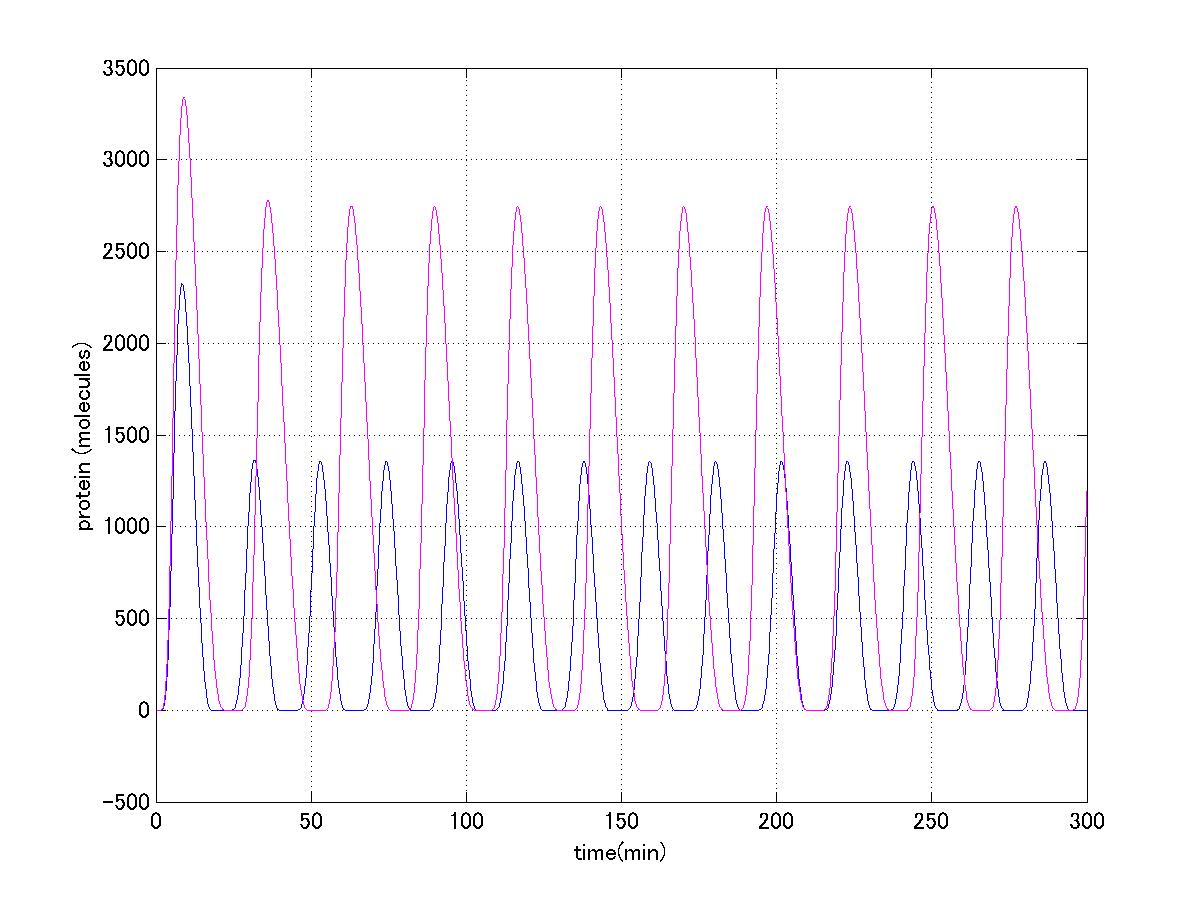

Supplement: Additional File 2 — AraC dimer oscillation time-course JPEG-format file of the two models at each arabinose and IPTG concentration. The reporter-less model is shown in blue and the reporter-containing model (Nd = 50) in red. [file 1752-0509-8-S4-S4-S2.zip › 1752-0509-8-S3-S5-S2/AraC_time_course_arabinose0.079433%_IPTG1mM.jpg]

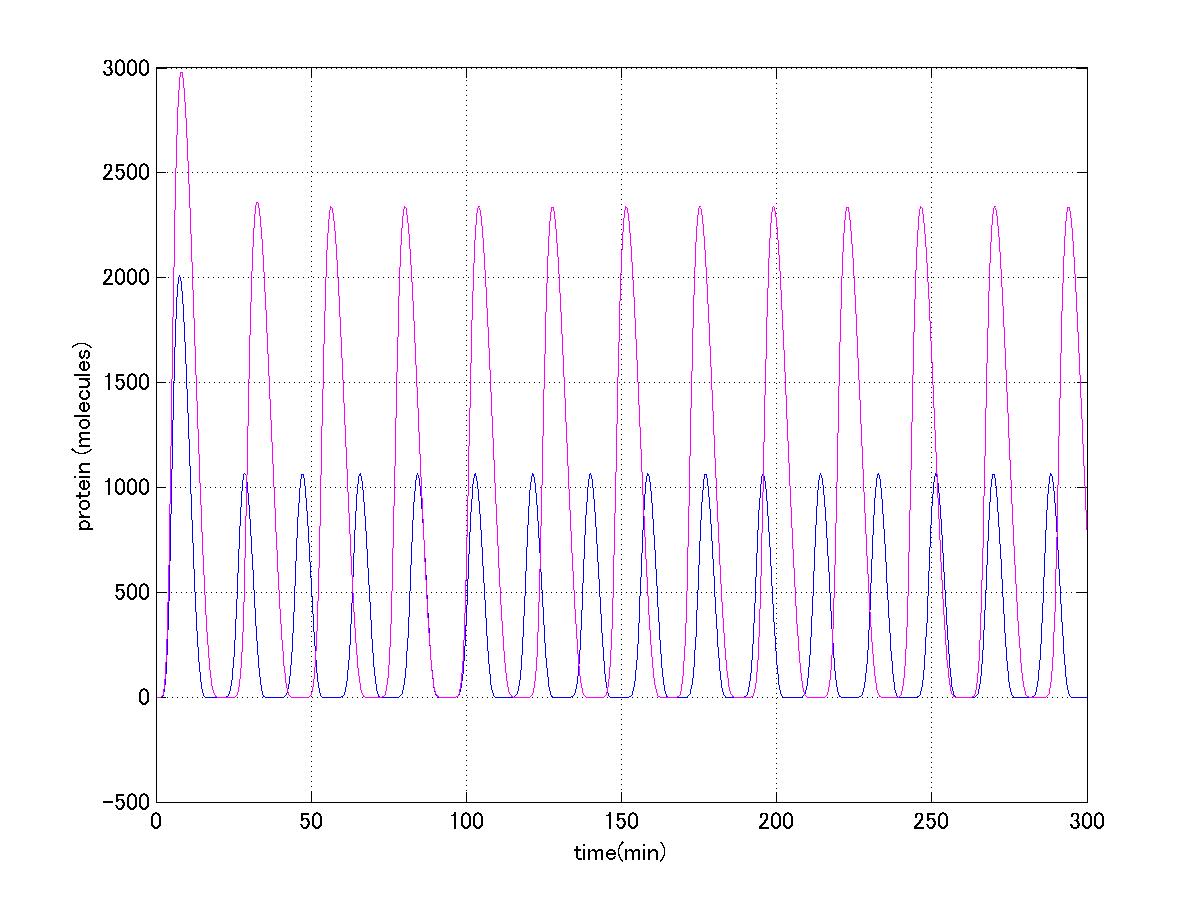

Supplement: Additional File 2 — AraC dimer oscillation time-course JPEG-format file of the two models at each arabinose and IPTG concentration. The reporter-less model is shown in blue and the reporter-containing model (Nd = 50) in red. [file 1752-0509-8-S4-S4-S2.zip › 1752-0509-8-S3-S5-S2/AraC_time_course_arabinose0.1%_IPTG0.12589mM.jpg]

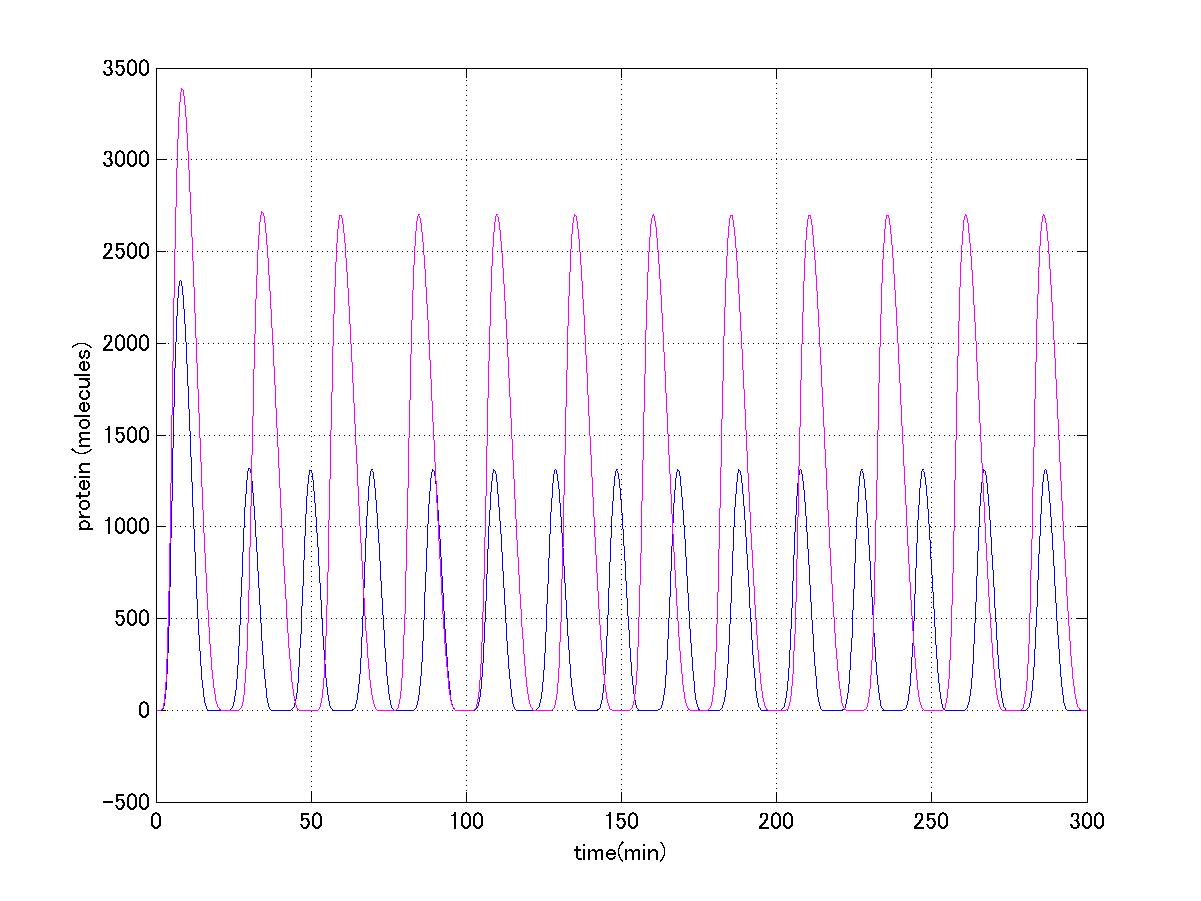

Supplement: Additional File 2 — AraC dimer oscillation time-course JPEG-format file of the two models at each arabinose and IPTG concentration. The reporter-less model is shown in blue and the reporter-containing model (Nd = 50) in red. [file 1752-0509-8-S4-S4-S2.zip › 1752-0509-8-S3-S5-S2/AraC_time_course_arabinose0.1%_IPTG0.15849mM.jpg]

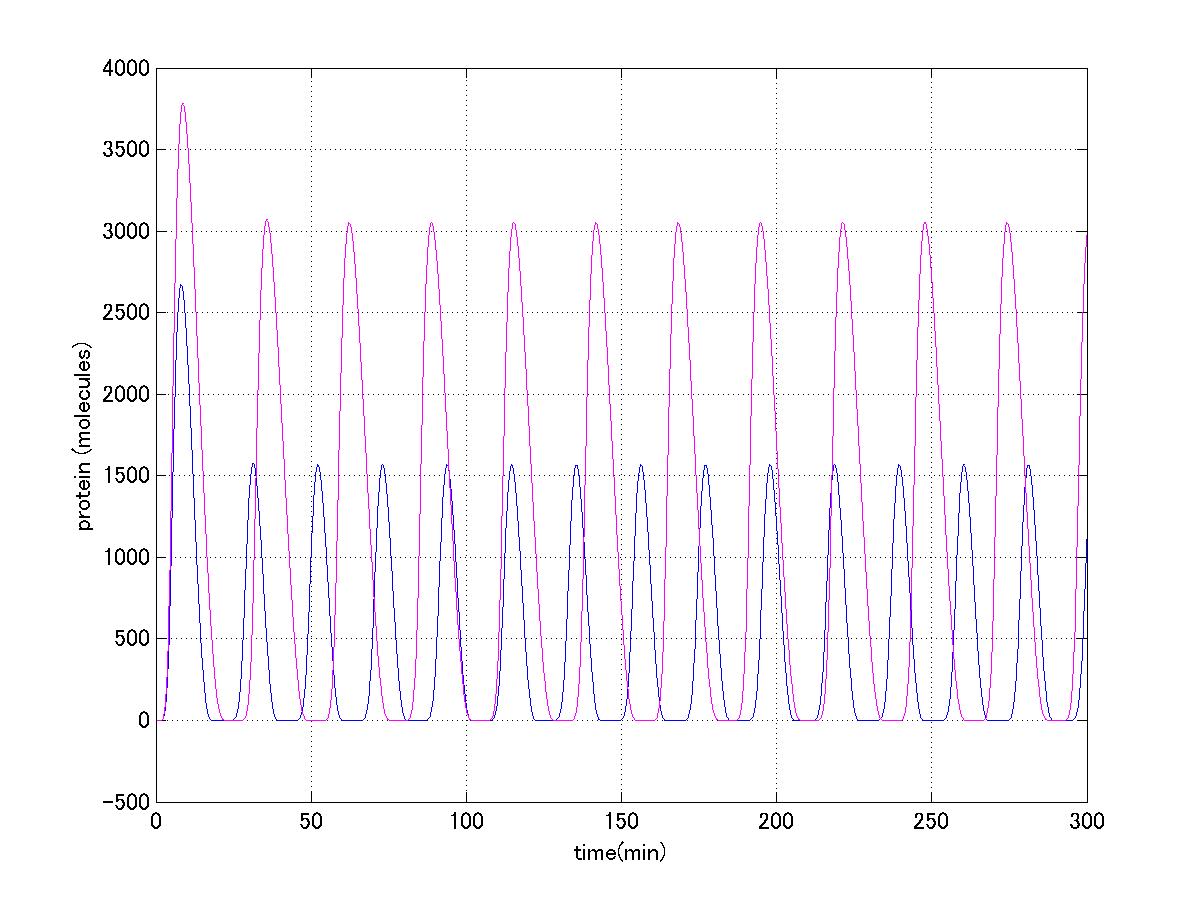

Supplement: Additional File 2 — AraC dimer oscillation time-course JPEG-format file of the two models at each arabinose and IPTG concentration. The reporter-less model is shown in blue and the reporter-containing model (Nd = 50) in red. [file 1752-0509-8-S4-S4-S2.zip › 1752-0509-8-S3-S5-S2/AraC_time_course_arabinose0.1%_IPTG0.19953mM.jpg]

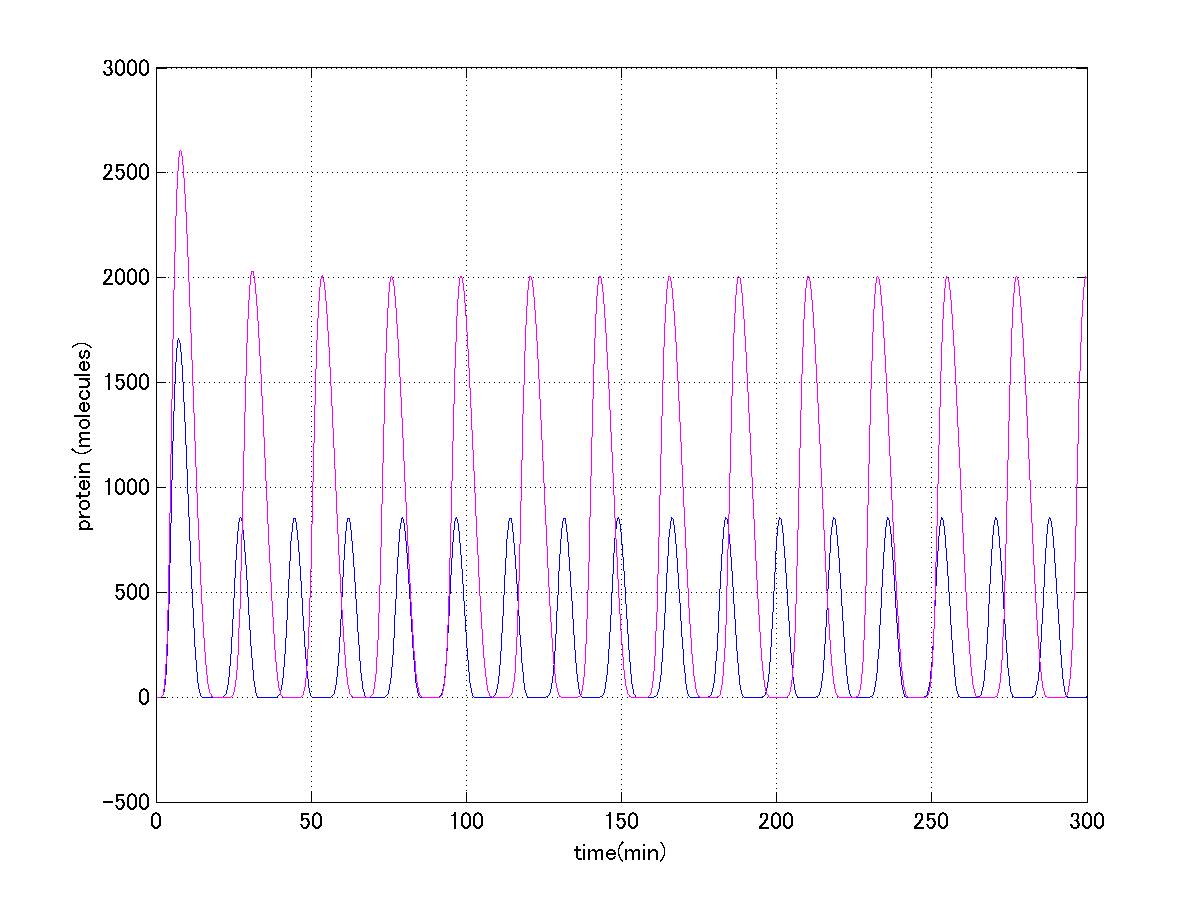

Supplement: Additional File 2 — AraC dimer oscillation time-course JPEG-format file of the two models at each arabinose and IPTG concentration. The reporter-less model is shown in blue and the reporter-containing model (Nd = 50) in red. [file 1752-0509-8-S4-S4-S2.zip › 1752-0509-8-S3-S5-S2/AraC_time_course_arabinose0.1%_IPTG0.1mM.jpg]

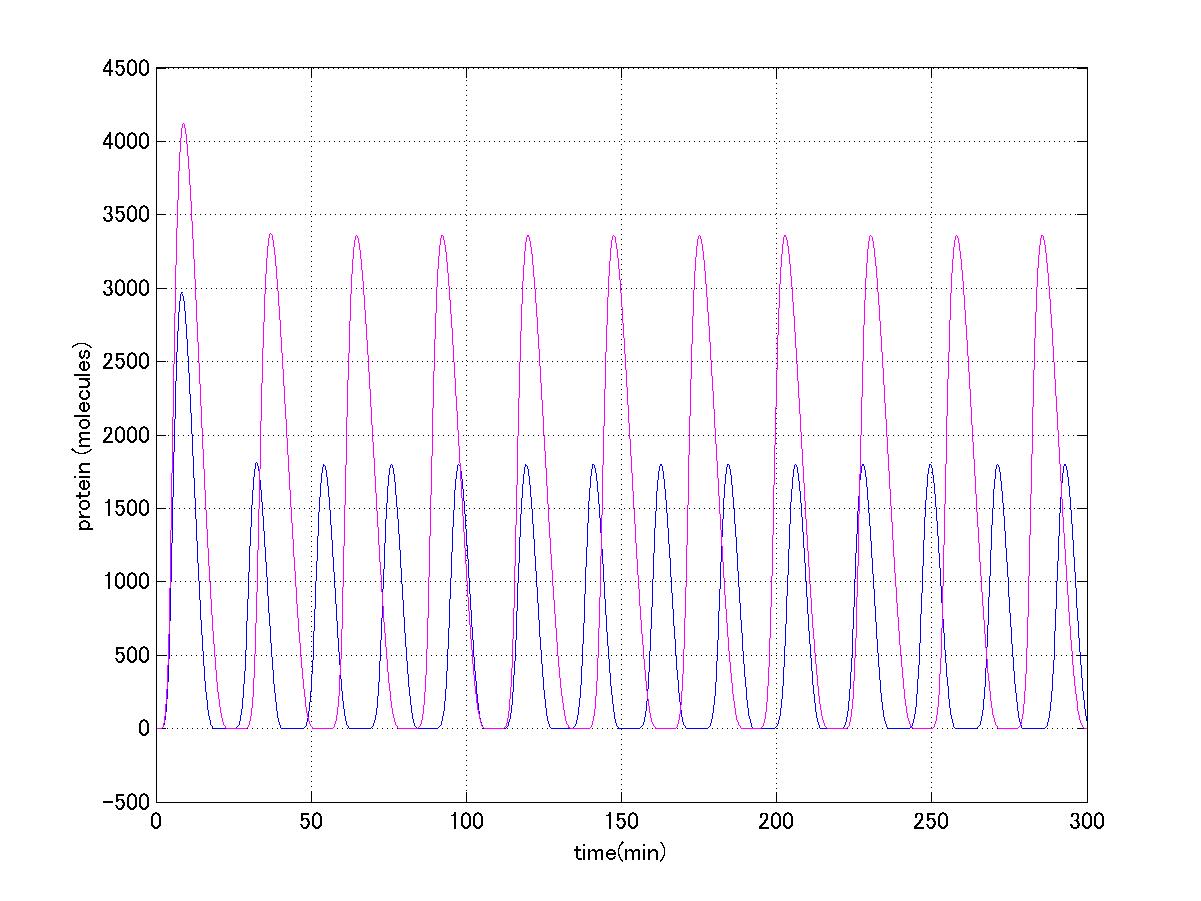

Supplement: Additional File 2 — AraC dimer oscillation time-course JPEG-format file of the two models at each arabinose and IPTG concentration. The reporter-less model is shown in blue and the reporter-containing model (Nd = 50) in red. [file 1752-0509-8-S4-S4-S2.zip › 1752-0509-8-S3-S5-S2/AraC_time_course_arabinose0.1%_IPTG0.25119mM.jpg]

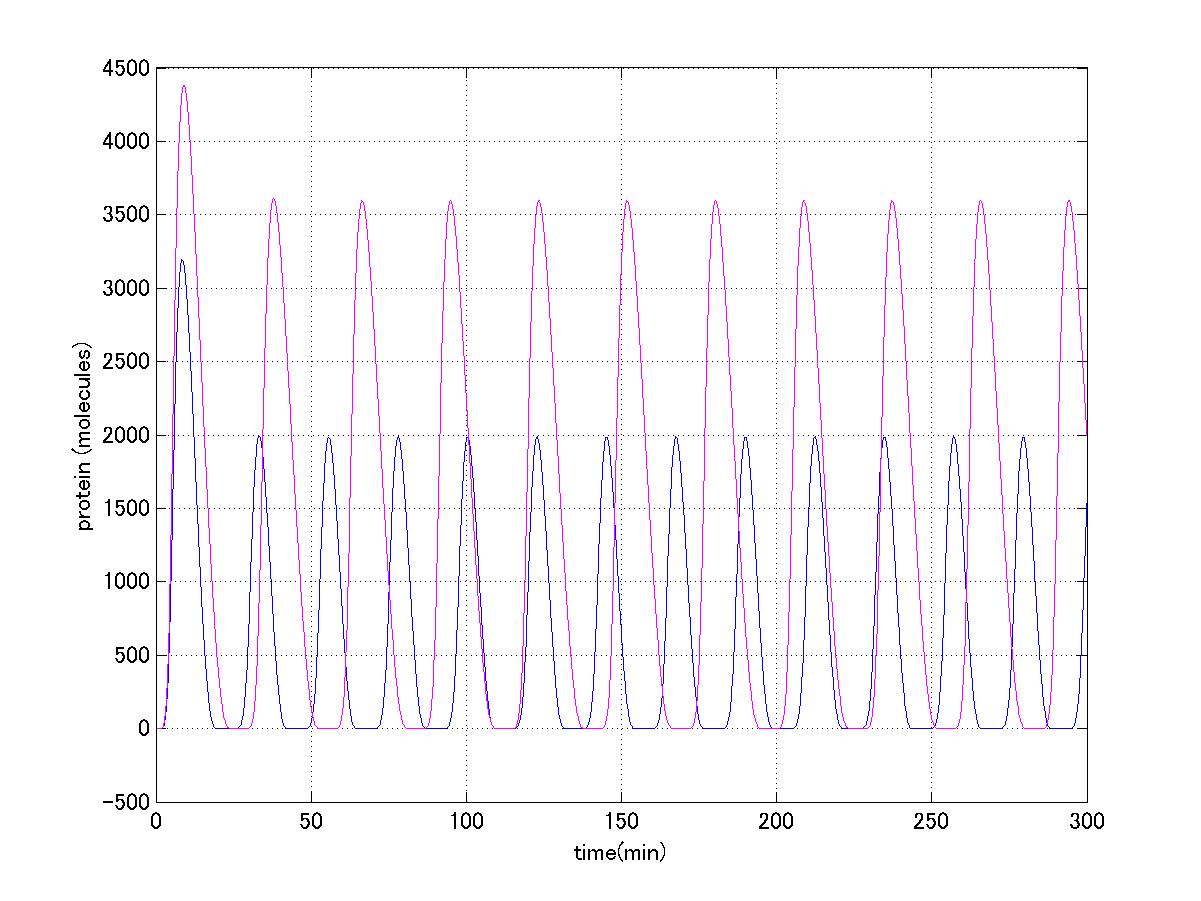

Supplement: Additional File 2 — AraC dimer oscillation time-course JPEG-format file of the two models at each arabinose and IPTG concentration. The reporter-less model is shown in blue and the reporter-containing model (Nd = 50) in red. [file 1752-0509-8-S4-S4-S2.zip › 1752-0509-8-S3-S5-S2/AraC_time_course_arabinose0.1%_IPTG0.31623mM.jpg]

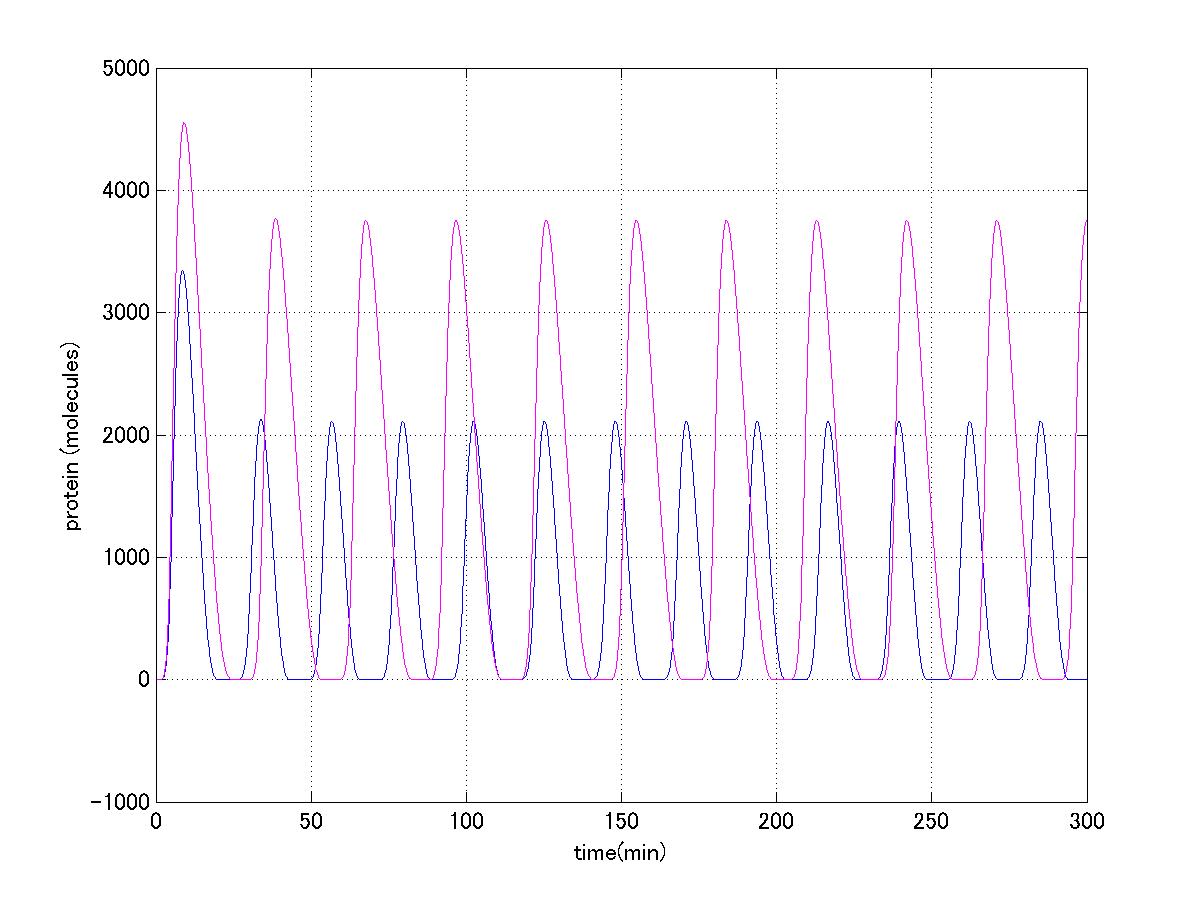

Supplement: Additional File 2 — AraC dimer oscillation time-course JPEG-format file of the two models at each arabinose and IPTG concentration. The reporter-less model is shown in blue and the reporter-containing model (Nd = 50) in red. [file 1752-0509-8-S4-S4-S2.zip › 1752-0509-8-S3-S5-S2/AraC_time_course_arabinose0.1%_IPTG0.39811mM.jpg]

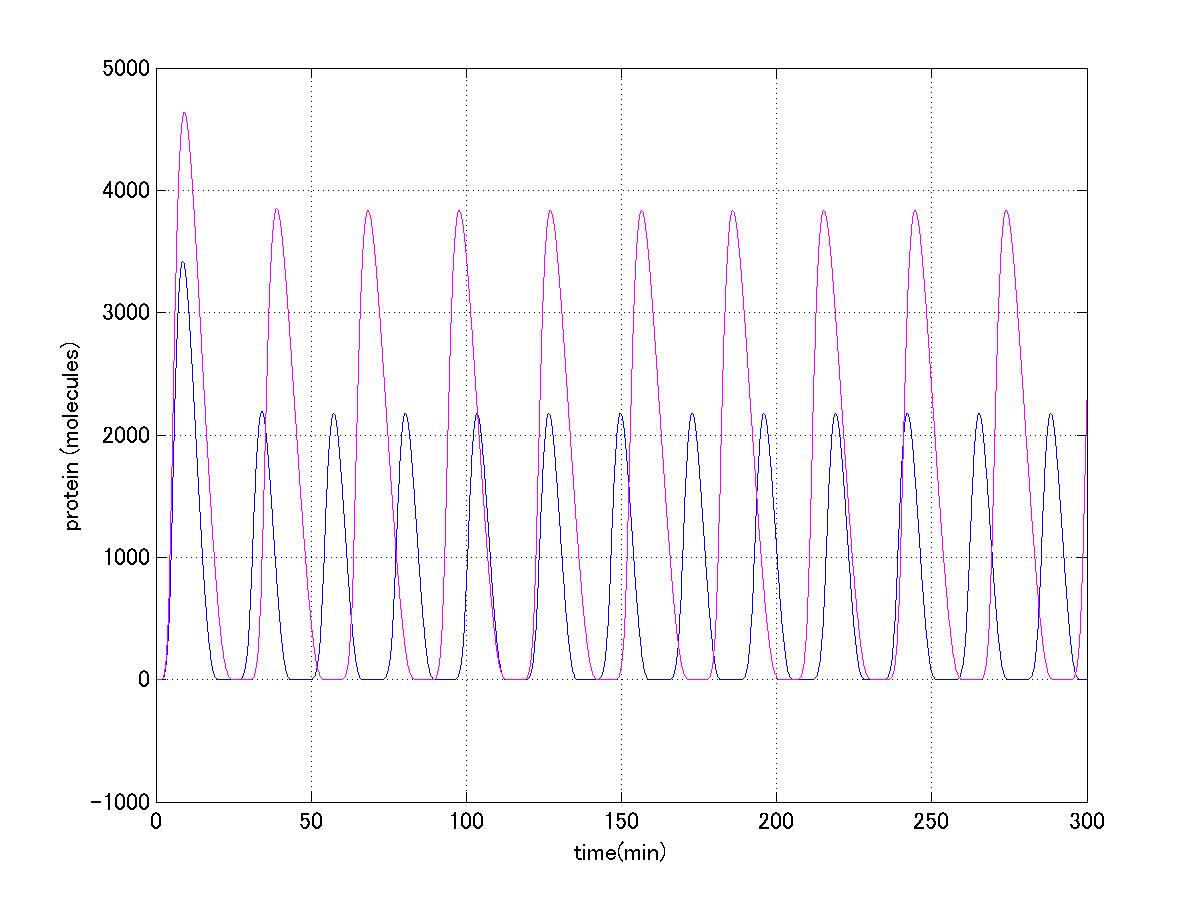

Supplement: Additional File 2 — AraC dimer oscillation time-course JPEG-format file of the two models at each arabinose and IPTG concentration. The reporter-less model is shown in blue and the reporter-containing model (Nd = 50) in red. [file 1752-0509-8-S4-S4-S2.zip › 1752-0509-8-S3-S5-S2/AraC_time_course_arabinose0.1%_IPTG0.50119mM.jpg]

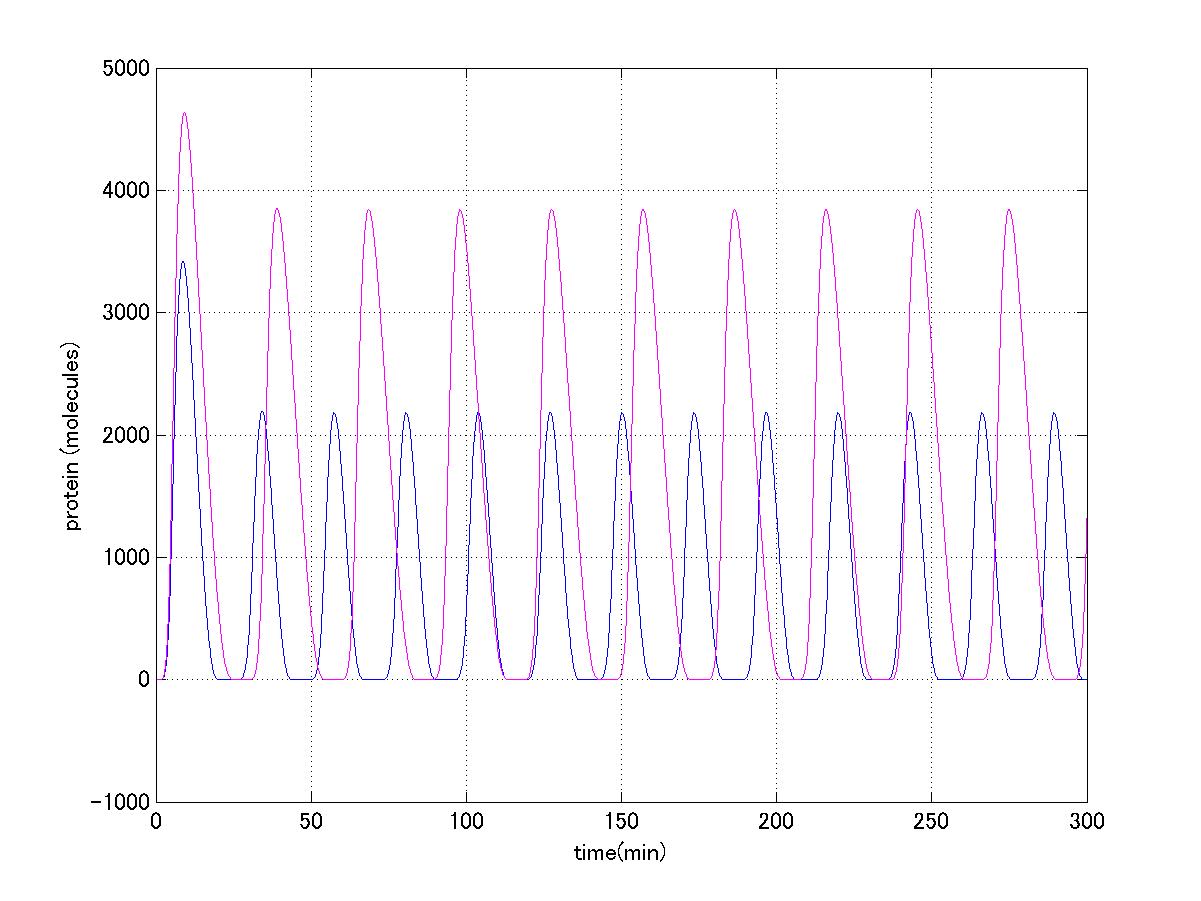

Supplement: Additional File 2 — AraC dimer oscillation time-course JPEG-format file of the two models at each arabinose and IPTG concentration. The reporter-less model is shown in blue and the reporter-containing model (Nd = 50) in red. [file 1752-0509-8-S4-S4-S2.zip › 1752-0509-8-S3-S5-S2/AraC_time_course_arabinose0.1%_IPTG0.63096mM.jpg]

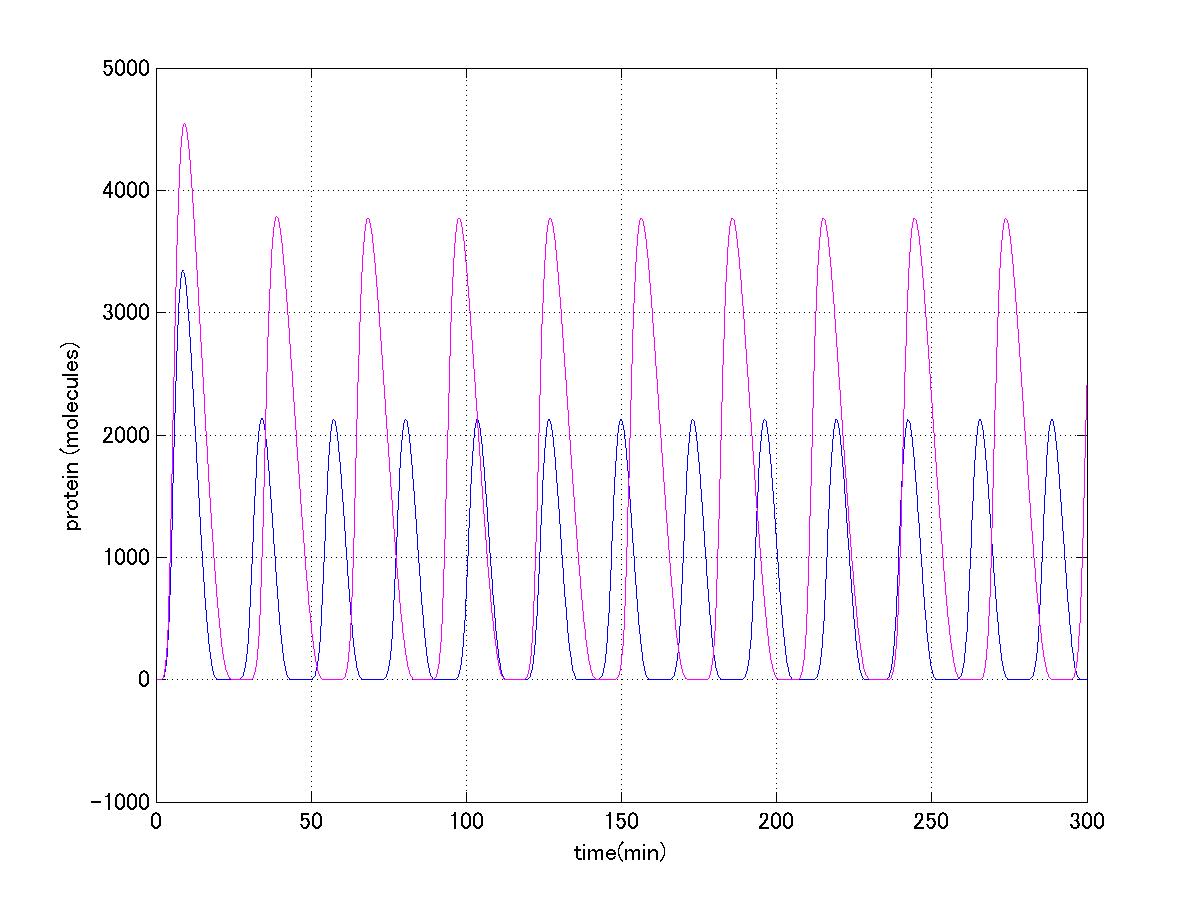

Supplement: Additional File 2 — AraC dimer oscillation time-course JPEG-format file of the two models at each arabinose and IPTG concentration. The reporter-less model is shown in blue and the reporter-containing model (Nd = 50) in red. [file 1752-0509-8-S4-S4-S2.zip › 1752-0509-8-S3-S5-S2/AraC_time_course_arabinose0.1%_IPTG0.79433mM.jpg]

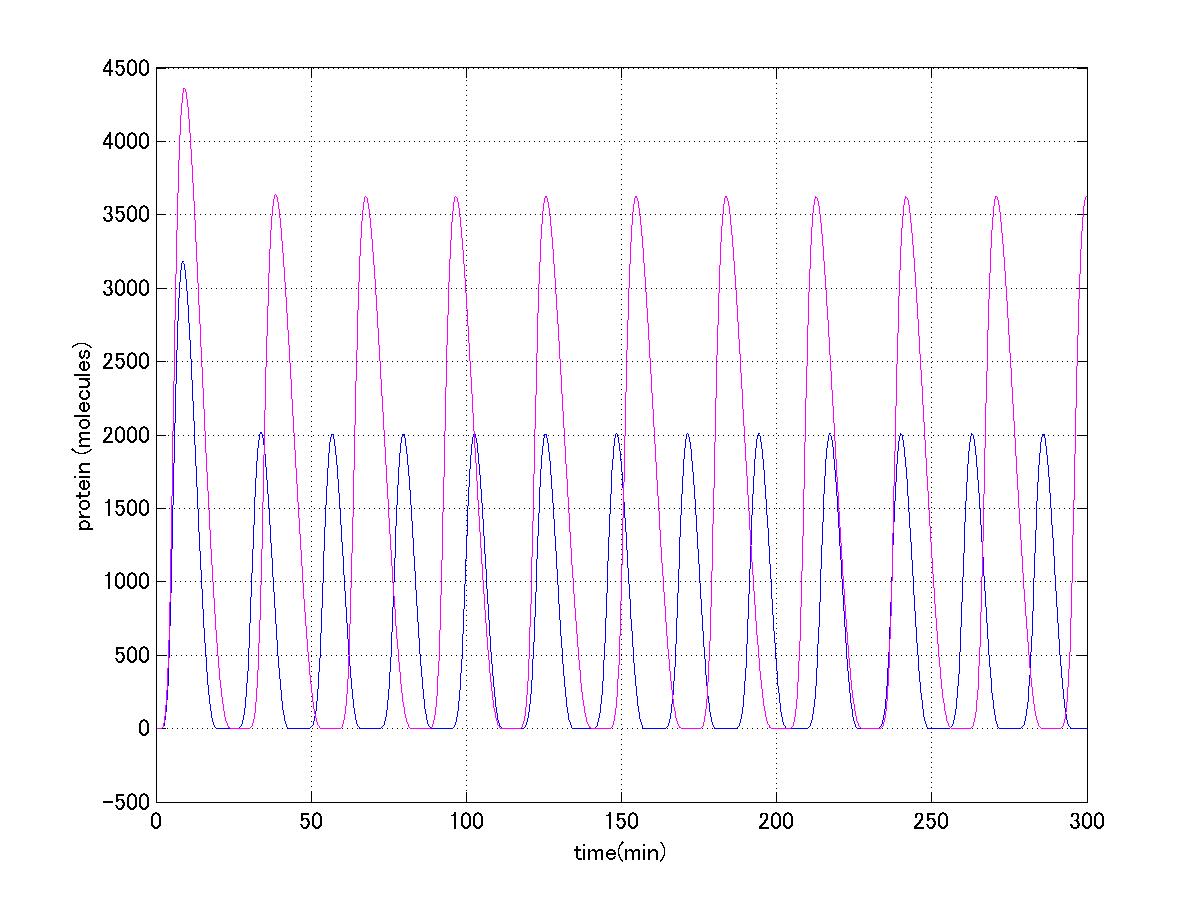

Supplement: Additional File 2 — AraC dimer oscillation time-course JPEG-format file of the two models at each arabinose and IPTG concentration. The reporter-less model is shown in blue and the reporter-containing model (Nd = 50) in red. [file 1752-0509-8-S4-S4-S2.zip › 1752-0509-8-S3-S5-S2/AraC_time_course_arabinose0.1%_IPTG1mM.jpg]
