# Supplementary material for: Effects of downstream genes on synthetic genetic circuits
Source: BMC Syst Biol. 2014 Dec 8;8(Suppl 4):S4. doi: 10.1186/1752-0509-8-S4-S4 (PMC4290693; doi:10.1186/1752-0509-8-S4-S4)
Supplement: Additional File 3 — Parameters used for the simulations. [file 1752-0509-8-S4-S4-S3.pdf]

Additional File 3. Parameters used for the simulations

Parameter name	Parameter value	Description	Units
ba, br, bd	0.36	transcription rate	min^{-1}
α	20	coefficient of active promoter	
k_{-a}, k_{-r}	1.8	protein–promoter unbinding rate	min^{-1}
t_a, t_r, t_d	90	translation rate	min^{-1}
d_a, d_r, d_d	0.54	mRNA degradation rate	min^{-1}
k_{fa}, k_{fr}, k_{fd}	0.9	folding rate	min^{-1}
k_{da}, k_{dr}, k_t	0.018	multimerization rate	$min^{-1}molecules^{-1}$
k_{-da}, k_{-dr}, k_t	0.00018	multimer dissociation rate	min^{-1}
k_l	0.36	loop forming rate	min^{-1}
k_{ul}	0.18	loop dissociation rate	min^{-1}
γ	1080	maximum degradation rate	$molecules \cdot min^{-1}$
c_e	0.1	dissociation constant	molecules
λ	2.5	coefficient of degradation rate	
ε	0.2	coefficient of degradation rate	
N_a	50	copy number of activator gene	
N_r	25	copy number of repressor gene	
N_d	50	copy number of downstream gene	
C_r^{max}	0.2	maximum affinity of LacI for the promoter	$molecules^{-1}$
C_r^{min}	0.01	minimum affinity of LacI for the promoter	$molecules^{-1}$

[IPTG]	0–10	IPTG concentration	mM
k_{r1}	0.035	correction constant of IPTG	mM
b_1	2	Hill coefficient of inhibition by LacI	
C_a^{max}	1	maximum affinity of AraC for the promoter	molecules^{-1}
C_a^{min}	0	minimum affinity of AraC for the promoter	molecules^{-1}
[ara]	0–2	arabinose concentration	% (w/v)
k_{a1}	2.5	correction constant of arabinose	% (w/v)
c_1	2	Hill coefficient of activation by AraC	
b_2	2	Hill coefficient of inhibition by LacI	
